# Intermediate hepatitis C virus (HCV) endemicity and its genotype distribution in Myanmar: A systematic review and meta-analysis

**DOI:** 10.1371/journal.pone.0307872

**Published:** 2024-09-19

**Authors:** Zayar Phyo, Ko Ko, Serge Ouoba, Aya Sugiyama, Ulugbek Khudayberdievich Mirzaev, Golda Ataa Akuffo, Chanroth Chhoung, Tomoyuki Akita, Junko Tanaka

**Affiliations:** 1 Department of Epidemiology, Infectious Disease Control and Prevention, Graduate School of Biomedical and Health Science, Hiroshima University, Hiroshima, Japan; 2 Project Research Center for Epidemiology and Prevention of viral hepatitis and hepatocellular carcinoma, Hiroshima University, Hiroshima, Japan; 3 Unité de Recherche Clinique de Nanoro (URCN), Institut de Recherche en Science de la Santé (IRSS), Nanoro, Burkina Faso; 4 Department of Hepatology, Scientific Research Institute of Virology, Tashkent, Uzbekistan; Centers for Disease Control and Prevention, UNITED STATES OF AMERICA

## Abstract

**Background:**

Comprehensive details on Hepatitis C virus (HCV) infection in Myanmar are lacking. This study determined the prevalence of HCV antibodies and ribonucleic acid (RNA) and the distribution of HCV genotypes across different populations in Myanmar from 1990 to 2023.

**Material and methods:**

A systematic search in PubMed, Web of Science, Scopus, and local journals identified studies reporting on HCV antibodies, RNA, and genotypes, excluding clinical research related to liver disease prognosis. Screening and data extraction was done by two authors and study populations were categorized into low-risk, high-risk, liver disease patients, and refugees outside the country. The pooled prevalence was performed by Dersimonian and Laird method using the R program. The publication bias was shown by funnel plot, the Egger test was used to assess the symmetry of the plot, and the heterogeneity was examined by the Cochran Q test and I^2^ index.

**Results:**

Out of 135 reports screened for eligibility, 35 reports comprising 51 studies were included in which 33 studies provided data on HCV seroprevalence in 685,403 individuals, 8 studies reported HCV RNA prevalence in 25,018 individuals, and 10 studies examined HCV genotypes in 1,845 individuals. The pooled seroprevalence of HCV among low-risk, high-risk, liver disease patients and refugees were 2.18%, 37.07%, 33.84%, and 2.52% respectively. HCV RNA-positive rates in these groups were 1.40%, 5.25%, 24.96%, and 0.84% respectively. Seroprevalence studies showed publication bias (Egger test, p = 0.0001), while RNA studies did not (Egger test, p = 0.8392). HCV genotype 3 was predominant in all sub-groups in Myanmar.

**Conclusion:**

Our study shows Myanmar has intermediate HCV endemicity with lowest HCV prevalence of 2.18% in low-risk groups and highest prevalence of 37.07% in high- risk groups. However, the findings highlight the need for further epidemiological studies to understand actual disease burden and implement effective countermeasures to achieve the WHO’s goal of HCV elimination by 2030.

## Background

Hepatitis C virus (HCV) is an enveloped positive-strand RNA virus belonging to the genus *Hepacivirus* in the family *Flaviviridae*. It causes liver inflammation, which can range from mild to severe, including cirrhosis and liver cancer. HCV is a bloodborne virus transmitted through exposure to infected blood, which can occur via unsafe injection practices, unsafe healthcare, unscreened blood transfusions, injection drug use, and sexual practices that involve contact with contaminated blood [1]. Globally, 58 million people are infected by HCV infection with approximately 1.5 million new infections each year [1]. According to the World Health Organization (WHO), only 20% of those infected with HCV have been diagnosed, leaving a significant number undiagnosed cases because of limited access to affordable testing [2]. The imperceptible barriers were still present in line with the elimination of viral hepatitis as a global action, rising as a public health concern. In 2020, the WHO Southeast Asia region had an estimated 10 million people living with chronic hepatitis C, resulting in 40,938 deaths annually and less than 1% of diagnosed viremic patients had started treatment [3].

Myanmar, a developing country in Southeast Asia, is rich in natural resources and home to a diverse population of 54.78 million people from various ethnic groups. Before 2017, there was no national program or strategic plan in Myanmar providing free-of-charge treatment for HCV. Although HCV antibodies and HCV RNA testing were available in private hospitals and laboratories, they are costly [4]. With the introduction of direct-acting antivirals (DAAs) which have a high success rate (>90% sustained virologic response: SVR) and fewer side effects, the Myanmar government initiated the free testing and antiviral treatment in seven public hospitals in 2017 [5]. This made Myanmar the first country in Southeast Asia region providing free HCV treatment. Various local and international non-governmental organizations have also joined efforts to eliminate HCV in Myanmar [6]. The Myanmar Liver Foundation (MLF) has been offering anti-HCV treatment to patients with chronic liver diseases through their Than Sitt Charity Clinics since 2017 [7]. Médecins Sans Frontières (MSF) has been providing anti-HCV treatment specifically to individuals co-infected with HIV in Myanmar since the same year [8]. Additionally, the Burnet Institute of Myanmar has been delivering HCV treatment to both the general population and people who inject drugs (PWID) since 2019 [6].

The epidemiological patterns of HCV infection in Myanmar are not well-documented. Between 1996 and 2017, reported prevalence rates of HCV antibodies varied widely, from the lowest 0.50% among blood donors (from an unpublished study) to the highest 79.19% [9] among PWID. A nationwide study in 2015 estimated the prevalence of HCV antibodies at 2.65% [10], suggesting that approximately 1.4 million people had been exposed to HCV. Given that about 30% of those infected naturally eliminate the virus within six months without treatment [1], the total number of chronic HCV infections was estimated to be around 0.9 million. These chronic infections can progress to severe complications such as liver cirrhosis and hepatocellular carcinoma (HCC) [11]. Since 2018, no new studies have been conducted on the burden of HCV infection in Myanmar. The external validity and representativeness of the findings remain uncertain as most of the existing studies employed the convenience sampling methods [12]. Furthermore, the lack of detailed information on HCV infection characteristics within specific populations and geographic regions poses a significant challenge for the effective implementation of targeted control strategies [13]. Therefore, this study aims to provide the baseline epidemiological data on the disease burden of HCV infection in Myanmar and to determine the prevalence of HCV antibodies and HCV RNA and to assess the distribution of HCV genotypes across various populations from 1990 to 2023.

## Materials and method

### Study design and guidelines

This systematic review was conducted following the 2020 Preferred Reporting Items for Systematic Reviews and Meta-Analysis (PRISMA) guidelines [14] (S1 Appendix). Though we did not prospectively register our systematic review in the registry databases such as International Prospective Register of Systematic Reviews (PROSPERO), International Platform of Registered Systematic Review and Meta-analysis Protocols (INPLASY) etc, there was no ongoing similar studies in those databases. Before the review, we searched databases such as PubMed, Web of Science, and Scopus for published studies on this topic and found none.

#### Data source and study selection

Searches in PubMed, Web of Science, and Scopus were conducted to identify the relevant records (S1 Table). Our review included studies conducted from January 1990 to May 2023 that reported on the prevalence of HCV antibodies, HCV RNA, and/or HCV genotypes, specifically including exact sample sizes and the number of positive cases. There were no restrictions on the type of study, and the subjects could be Myanmar citizens living inside or outside the country. However, clinical research or studies focusing on the prognosis of liver diseases in HCV-infected patients were excluded. Other factors such as gender, age, work nature, and place of living did not affect the eligibility. Only reports in English were considered, and manual searches were conducted in The Myanmar Health Sciences Research Journal, Google Scholar, reference lists of eligible reports, and government reports. The last search was conducted on May 31, 2023, and duplicate records were removed using the Rayyan–Intelligent Systematic Review tool. Two independent investigators (ZP and KK) screened the records based on titles and abstracts, and relevant reports were retained for full-text review. In cases of disagreements, a third investigator (JT) made the final decision.

#### Data extraction

All relevant raw data, including authors’ names, year of publication, study design, sampling method, study period, characteristics of study subjects (age, sex, location, comorbidities), sample size, number of participants positive for HCV antibodies/HCV RNA/HCV genotype, and type of biological assay, were extracted from each recruited study using Microsoft Excel 2010. The data were counter-checked by a second author. In this study, a "report" refers to any document mentioning HCV prevalence/genotype data, while a "study" pertains to the measurement of HCV antibodies/HCV RNA/HCV genotype in a specific population. Consequently, one report may describe multiple studies, and data were extracted separately for each study. In cases where study subjects were found to overlap in two or more different studies, only one publication was included.

#### Quality assessment of the included studies

The Joanna Briggs Institute checklist for prevalence studies was used to assess the quality of the included studies [15] (S2 Table). The review process unified the minimum acceptable information for each variable. The minimum sample size for prevalence estimation was set at 312 subjects, calculated based on an assumed seroprevalence of 2.65% [10], a precision of 1.8%, and a confidence level of 95%. The sampling procedure of each study was examined in detail, and studies employing probabilistic sampling were considered representative of the target population. The validity of seroprevalence and genotype data relied on whether the diagnostics were based on standard procedures and methods.

### Statistical analysis

The study populations were categorized into four groups based on the characteristics of subjects: (1) low-risk group, (2) high-risk group, (3) liver disease patients, and (4) refugees/immigrants. The low-risk group included the general population, blood donors, and hepatitis B virus (HBV) vaccine non-responders. The high-risk group contained people living with human immunodeficiency virus (PLHIV), PWID, patients who received blood transfusions, and haemodialysis patients. The other two groups were simply categorized as liver disease patients and refugees/immigrants. The statistical analysis was conducted using the ’meta’ and ’metafor’ packages in R programming. The Freeman-Tukey double arcsine method was used to transform the data to accommodate small proportions. The meta-analysis was performed using the Dersimonian and Laird method, which utilizes a random-effects model, and the results were presented in a forest plot. For the low-risk group, which included three unpublished papers with a total sample size of 72,680, a sensitivity analysis was performed by excluding these unpublished papers. Confidence intervals (CI) for the proportions of individual studies were calculated using the Clopper-Pearson method. The Cochran Q test was employed to evaluate heterogeneity and then its degree was quantified by the I^2^ index. Heterogeneity was considered significant if the p-value of the Cochran Q test was less than 0.05. The high, medium, or low level of heterogeneity was identified by the I^2^ index thresholds of 75%, 50%, and 25% respectively [16]. A funnel plot of the transformed proportion against the sample size was generated to assess publication bias [17]. The symmetry of the plot was evaluated using the Egger test, with significance determined at a threshold of p < 0.1.

### Inclusivity in global research

Additional information regarding the ethical, cultural, and scientific considerations specific to inclusivity in global research is included in the Supporting Information (S1 Checklist).

### Ethical approval and consent to participate

Not applicable.

## Results

### Search results

**Fig 1:**
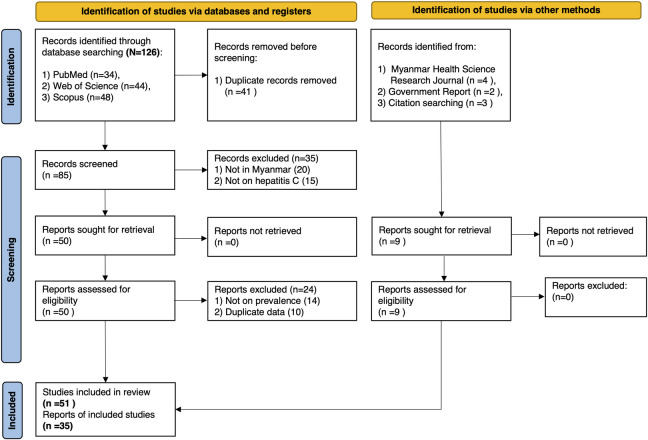
PRISMA showing a selection of articles for meta-analysis of HCV in Myanmar. This figure shows the selection process of the articles for the estimation of HCV prevalence in Myanmar. Each arrow indicates the step-by-step process for recruiting the articles and the detailed reason for the exclusion of articles were described with their respective exact number. This figure was built following the guideline for 2020 Preferred Reporting Items for Systematic Reviews and Meta-Analysis (PRISMA).

A total of 126 articles were identified through the database search. Nine articles from the Myanmar Health Sciences Research Journal and government reports were manually added. After removing the duplicate and irrelevant articles, 85 articles were screened by their abstracts resulting in the exclusion of 35 articles based on the strict exclusion criteria. The remaining 50 articles were accessed for full text review and carefully evaluated for eligibility. Ultimately, 35 articles met the criteria and were included in the analysis to estimate the prevalence of HCV antibodies, HCV RNA and the distribution of HCV genotype in Myanmar (Fig 1). Some studies indicated the exact number of males and females or the place from which the samples were taken and also stated the age-specific number of samples and number of positive participants and corresponding prevalence, but most of the studies had lack of such information and it was quite difficult to divide the study subjects by their sex, age, and place of living. In terms of time series for included reports, 5, 9, and 13 reports were conducted during 1990–2000, 2001–2010, and 2011–2020 respectively but 8 reports did not describe the study year [18–24] (Table 1).

**Table 1:** Characteristics of included studies reporting the prevalence of HCV and genotype distribution in Myanmar between 1990 and 2023.

First author, year of publication	Study design	Sampling method	Data collection period	Study subjects	Mean age or range (in years)	Sample size	Number of HCV-Ab positive	Number of HCV RNA- positive	Number of amplified samples	Assay for HCV-Ab/RNA testing
J Mellor, 1996 [18]	C	Convenient	NR	Blood donors	NR	8	-	-	8	-
KP Kyi, 1998 [25]	CS	Consecutive	1991	HCC	20–89	40	14	-	-	ELISA
M Khin, 2000^*†*^	CS	Convenience	NR	General population	1–74	569	16	-	-	NR
O Shigeru, 2000a^^^ [26]	CS	NR	1996–1998	Thalassemia	7±3.5	15	7	-	-	ELISA
O Shigeru, 2000b^^^ [26]	CS	NR	1996–1998	Blood receipts	20±9	18	10	-	-	ELISA
O Shigeru, 2000c^^^ [26]	CS	NR	1996–1998	Liver diseases*	3–60	74	32	-	-	ELISA
O Shigeru, 2000d^^^ [26]	CS	NR	1996–1998	HCC	47±12	28	8	-	-	ELISA
N Win, 2000 [27]	CS	NR	1996	HCC	23–70	16	7	-	-	ELISA
N Kazuhiko, 2001a^^^ [28]	CS	Convenience	1998–2000	Blood donors	7–80	213	-	4	-	PCR
N Kazuhiko, 2001bd^^^ [28]	CS	Convenience	1998–2000	Liver diseases*	7–80	165	-	42	24	PCR
N Kazuhiko, 2001c^^^ [28]	CS	Convenience	1998–2000	HCC	7–80	25	-	6	-	PCR
T Kazuhisa, 2002a^^^ [20]	CS	NR	NR	Liver diseases*	NR	29	14	-	-	IRMA
T Kazuhisa, 2002b^^^ [20]	CS	NR	NR	HCC	NR	20	4	-	-	IRMA
KP Kyi, 2002a^^^ [19]	CS	Consecutive	NR	General population	NR	362	9	-	-	Immunoblot
KP Kyi, 2002b^^^ [19]	CS	Convenience	NR	Liver diseases*	NR	379	95	-	-	Immunoblot
M Khin, 2003^*†*^	CS	Consecutive	2000–2004	Blood donors	NR	154,161	4,008	-	-	NR
T Shinji, 2004 [20]	RC	Convenient	2000	Blood donors	18–51	201	-	-	110	-
AA Lwin, 2007ab^^^ [21]	CS	Convenience	NR	General population	1–75	1,333	154	-	145	PA
A Denburg, 2007 [29]	C	Consecutive	2006	Refugees	NR	68	0	-	-	NR
A Thu, 2008 [9]	CS	Convenience	2007–2008	PWID	29.49±6.3	298	236	-	-	Rapid test
NJ Chaves, 2009 [30]	RC	Consecutive	2004–2008	Refugees	16–86	145	-	4	-	PCR
M Khin, 2010 [31]	CS	Consecutive	2005–2007	Blood donors	18–60	65,237	621	-	-	Rapid test
A Srunthron, 2010abc^^^ [32]	CS	Consecutive	2007–2009	Immigrants	15–60	1,594	27	15	15	ELISA (HCV-Ab) PCR (HCV RNA)
MM Htun, 2010ab^^^ [22]	CS	Consecutive	NR	HBV vaccine non-responders	NR	23	0	1	-	PA (HCV-Ab) PCR (HCV RNA)
YH Zhou, 2011 [33]	CS	Convenience	2005–2007	PWID	31.8±9.8	318	153	-	-	ELISA
GA Paxton, 2012 [34]	CS	Consecutive	2007–2009	Refugees	0.5–87	519	10	-	-	NR
TM Hayden, 2014ab^^^ [35]	CS	Convenience	2002–2007	Refugees	15–95	1,076	-	4	4	PCR
MOH Myanmar, 2015^*†*^	CS	Consecutive	2015	Blood donors	15–80	379,088	1,895	-	-	Rapid test
C Naing, 2015 [23]	RC	Convenient	NR	Chronic hepatitis C	47.1±11.6	362	-	-	350	-
AA Lwin, 2017 [10]	CS	Random	2015	General population	48 ± 14.6	5,547	147	-	-	ICT
AA Lwin, 2018 [36]	CS	NR	2016	Haemodialysis patients	20–40	111	17	-	-	ICT
NS Aye, 2018 [37]	RC	NR	2015–2017	PWID	24–34	531	376	-	-	Rapid test
CC Ngo, 2018 [38]	RC	Consecutive	2013–2014	Refugees	30–70	51	5	-	-	NR
HS Juon, 2019 [39]	CS	Convenience	2009–2015	Immigrants	37.3 ± 13	927	45	-	-	NR
M Ye, 2019ab^^^ [40]	CS	NR	2014	PWID	16–74	100	40	-	25	ELISA
M Chen, 2019 [41]	CS	Convenient	2014	PWID	22–33	7	-	-	7	-
Myanmar IBBS, 2019 [42]	CS	RDS	2017–2018	PWID	34.62 ± 9.25	6,061	3,394	-	-	Rapid test
KT Nyunt, 2019 [24]	CS	NR	NR	General population	35.3	178	1	-	-	ICT
TS Win, 2020 [43]	CS	NR	2016	PLHIV	35 (30–42)	15	150	-	-	ELISA
NTT Kyaw, 2022 [44]	C	Consecutive	2005–2016	PLHIV	1–65	27,722	2,265	-	-	OraQuick
K Urban, 2023 [45]	CS	Consecutive	2010–2017	Refugees	NR	18,059	511	-	-	NR
TM Swe, 2023abc^^^ [8]	C	Consecutive	2016–2017	PLHIV	15–80	21,777	1,417	1,143	640	OraQuick (HCV-Ab) PCR (HCV RNA)

*HCV-Ab*, hepatitis C virus antibodies; *HCV RNA*; hepatitis C virus ribonucleic acid; *MOH*, Ministry of Health; *IBBS*, integrated biological and behavioral survey; *CS*, cross-sectional; *C*, cohort; *RC*, retrospective cohort; *NR*, not reported; *RDS*, respondent driven sampling; *HBV*, hepatitis B virus; *HCV*, hepatitis C virus; *HCC*, hepatocellular carcinoma; *PWID*, people who inject drugs; *PLHIV*, people living with human immunodeficiency virus; *ELISA*, enzyme-linked immunosorbent assay; *PCR*, polymerase chain reaction; *ICT*, immunochromatography; *IRMA*, immunoradiometric assay; *CLIA*, chemiluminescence immunoassay; *PA*, particle agglutination.

*Patients with liver diseases other than hepatocellular carcinoma

^*†*^ Unpublished observations

^^^Studies issued from the same report.

### Study characteristics

Out of a total of 135 reports screened for eligibility, 35 reports comprising 51 studies were included in which 33 studies provided data on HCV seroprevalence in 685,403 individuals, 8 studies reported HCV RNA prevalence in 25,018 individuals, and 10 studies examined HCV genotypes in 1,857 individuals. The studies included in the analysis were conducted from 1991 to 2018 (Table 1). In HCV antibodies and RNA prevalence studies, the cross-sectional design was primarily used in 34 out of 41 studies (82.9%), while only one study [10] (2.4%) employed a probabilistic sampling method. In terms of sample size, 53.7% (22/41) of the studies included adequate sample sizes (>312). HCV antibodies and HCV RNA were determined through biological assays which were reported in 83.0% (34/41) of the studies (S1 Table).

Regarding the estimation of HCV seroprevalence, 28 out of 33 studies conducted cross-sectional studies, two studies utilized retrospective cohort designs in which the prevalence was estimated from laboratory records, and the remaining three studies employed cohort studies where HCV seroprevalence was determined as part of the overall study (Table 1). For the HCV RNA prevalence study, six out of eight studies performed cross-sectional studies, one study adopted a retrospective cohort, and one study employed a cohort study (Table 1). Out of the ten studies conducted for the HCV genotype study, five utilized cross-sectional study designs, three conducted a retrospective cohort approach, and two employed a cohort study methodology (Table 1). All included reports covered a study period ranging from 1991 to 2018, with the majority of studies conducted after 2010.

In terms of participant characteristics in the HCV seroprevalence estimate, nine studies focused on low-risk groups, including the general population, blood donors, and individuals who did not respond to HBV vaccines, encompassing a total of 60,648 participants. Eleven studies targeted high-risk groups, such as PWID, PLHIV, patients who received blood transfusions, and individuals undergoing hemodialysis, with a total of 57,101 participants. Seven studies focused on liver disease patients, specifically HCC, liver cirrhosis, and acute/chronic hepatitis, including 586 patients. Finally, six studies targeted refugees/immigrants, comprising a total of 21,218 individuals (Table 1).

In the HCV RNA prevalence study, two studies focused on low-risk groups, including blood donors and individuals who did not respond to HBV vaccines, with a total of 236 subjects. One study targeted a high-risk group of PLHIV, involving 21,777 individuals. Two studies focused on liver disease patients, including one study on HCC and one study on other liver diseases, encompassing 190 patients. Additionally, three studies specifically targeted refugees/immigrants, comprising a total of 2,815 individuals (Table 1).

In the study on HCV genotypes, three studies examined low-risk groups, which included the general population and blood donors, with a combined sample size of 363 subjects. Another three studies focused on high-risk groups such as PWID and PLHIV, involving 911 individuals. Two studies concentrated on patients with liver disease, encompassing a total of 552 patients. Furthermore, two studies specifically targeted refugees, with a total of 19 participants (Table 1).

### Seroprevalence of HCV antibodies

#### Seroprevalence in the low-risk group

**Fig 2:**
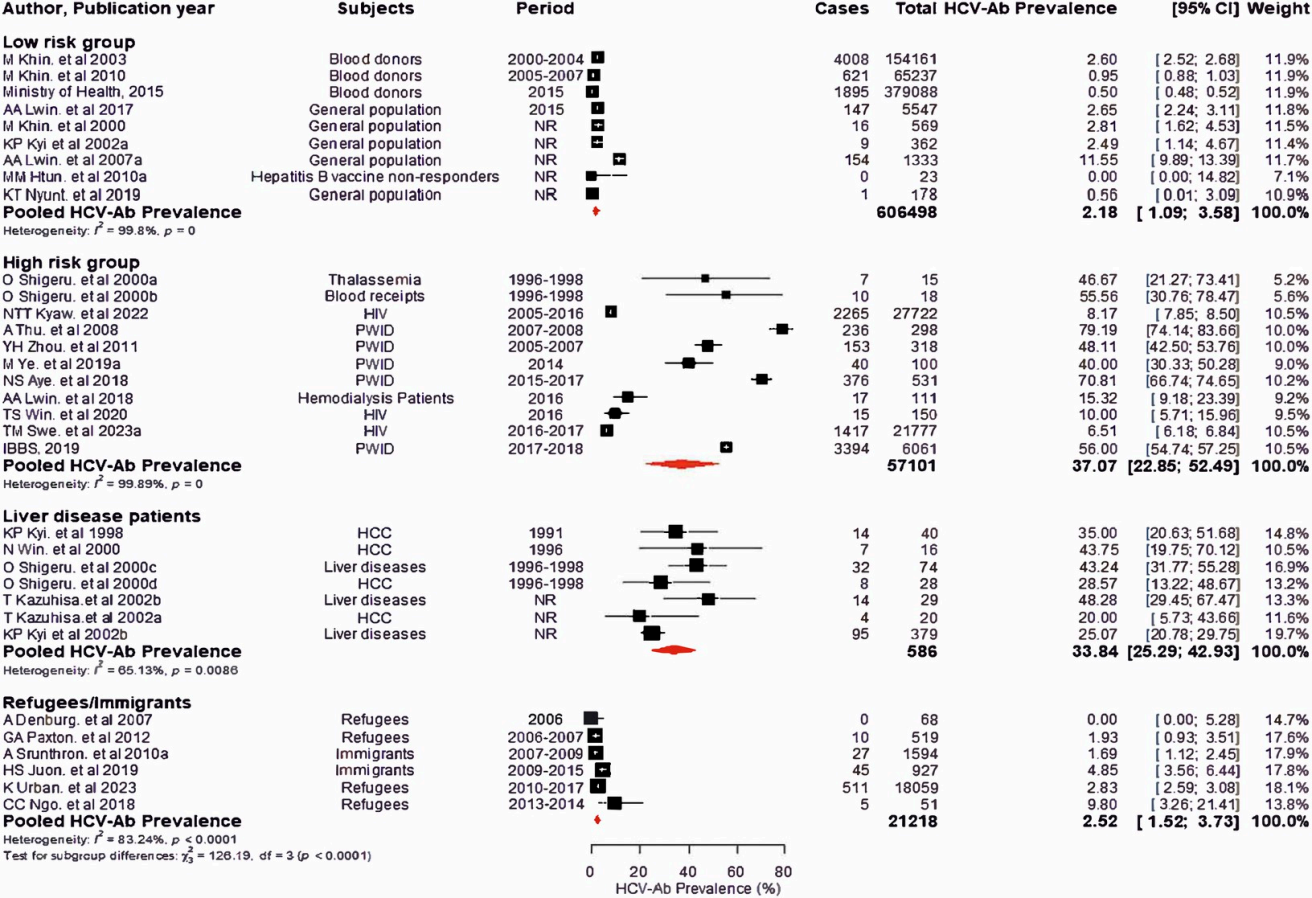
Forest plot of the pooled HCV seroprevalence in Myanmar, using random effect. This figure shows the funnel plot of 33 studies and the pooled HCV seroprevalence in sub-groups: low-risk group, high-risk group, liver disease patients, and refugees/immigrants. The diamond block represents for pooled prevalence and the rectangular block represents prevalence for each study.

Low-risk populations were addressed in 9 studies and the total number of subjects screened was 606,498 of which 6,851 subjects were positive. Five studies focused on the general population, three on blood donors, and one on HBV vaccine non-responders. The seroprevalence ranged between 0 and 11.55% and the pooled seroprevalence of HCV was estimated at 2.18% (95%CI: 1.09–3.58). However, significant heterogeneity was found in this group (I^2^ = 99.8%, p = 0) (Fig 2). A sensitivity analysis indicated that the prevalence of HCV antibodies increased to 2.41% (95% CI: 0.51%-5.4%) after excluding 3 unpublished reports (n = 72,680). However, this difference was not statistically significant (the result not shown).

#### Seroprevalence in the high-risk group

Individuals in the high-risk group were investigated in eleven studies: five on PWID, three on PLHIV, two on patients who received blood transfusion, and one on hemodialysis patients. A total of 57,101 high-risk individuals were studied, and 7,930 were HCV antibodies positive. Seroprevalence varied from 6.51% to 79.19%, and the pooled seroprevalence was 37.07% (95%CI: 22.85–52.49). Heterogeneity was also significant in this group (I^2^ = 99.89%, p = 0) (Fig 2).

#### Seroprevalence in the liver disease patients

Liver disease patients were investigated in seven studies: four on HCC patients and three on other liver disease patients. Out of a total of 586 patients, 174 were HCV antibodies positive. Seroprevalence varied from 20.00% to 48.28%, and the pooled seroprevalence was 33.84% (95% CI: 25.29–42.93). Heterogeneity was also significant in this group (I^2^ = 65.13%, p = 0.0086) (Fig 2).

#### Seroprevalence in the immigrants/refugees

Myanmar citizen immigrants/refugees who are living outside the country were investigated in six studies. Among a total of 21,218 individuals, 598 were HCV antibodies positive. Seroprevalence varied from 0 to 9.80% and the pooled seroprevalence was 2.52% (95%CI:1.52–3.73). Heterogeneity was also significant in this group (I^2^ = 83.24%, p<0.0001) (Fig 2).

#### Seroprevalence by study subjects

The highest HCV antibodies positive rate was found in PWID (58.06%, 95% CI: 56.94–59.18), individuals receiving blood transfusion (51.53%, 95% CI: 34.55–68.52), and liver disease patients (28.80%, 95% CI: 25.19–32.42). The seroprevalence of HCV among the general population was 2.82% (95% CI: 2.46–3.18, I^2^ = 96.57%, p < 0.0001), and that among blood donors was 0.68% (95% CI: 0.66–0.70, I^2^ = 99.92%, p<0.00001) (Table 2).

**Table 2:** HCV seroprevalence in sub-groups.

Categories	Number of studies	Total population	Pooled seroprevalence	95% CI	I^2^ index (%)	Cochran Q test p-value
***Study subjects***						
General population	5	7,989	2.82	2.46–3.18	96.57	<0.00001
Blood donors	3	598,486	0.68	0.66–0.70	99.92	<0.00001
HBV vaccine non-responders	1	23	0.00	0.00–0.00	NA	NA
PWID	5	7,308	58.06	56.94–59.18	97.49	<0.00001
PLHIV	3	49,649	7.36	7.13–7.59	96.12	<0.00001
Patients who received blood transfusion	2	33	51.53	34.55–68.52	NA	NA
Haemodialysis patients	1	111	15.32	8.62–22.02	NA	NA
Liver disease patients*	7	586	28.80	25.19–32.42	63.87	0.01
Refugees/immigrants	6	21,218	2.72	2.50–2.94	79.70	0.0002
***Data collection period*** ^***†***^						
2000–2005	1	154,161	2.60	2.52–2.70	NA	NA
2006–2010	1	65,237	0.95	0.90–1.03	NA	NA
2011–2015	2	384,635	0.51	0.48–0.53	99.99	<0.00001

*HBV*, hepatitis B virus; *PWID*, people who inject drugs; *PLHIV*, people living with human immunodeficiency virus; *NA*, not applicable.

* Patients with liver diseases including hepatocellular carcinoma

^*†*^ Analyses performed considering only low-risk groups (blood donors and general population).

#### Seroprevalence by the study period of data collection

Only low-risk groups were considered for HCV seroprevalence estimate by the study period to see the pure trend of its prevalence. One unpublished study was conducted between 2000–2005, resulting in a seroprevalence of 2.60% (95%CI: 2.52–2.70). The seroprevalence decreased to 0.95% (95%CI: 0.90–1.03, one study [31]) during 2006–2010 and to 0.51% (95%CI: 0.48–0.53, I^2^ = 99.99%, p<0.00001) during 2011–2015 (two studies including one unpublished study [10]) (Table 2). This decreasing trend was significant (p for difference = 0.03).

### HCV RNA prevalence

**Fig 3:**
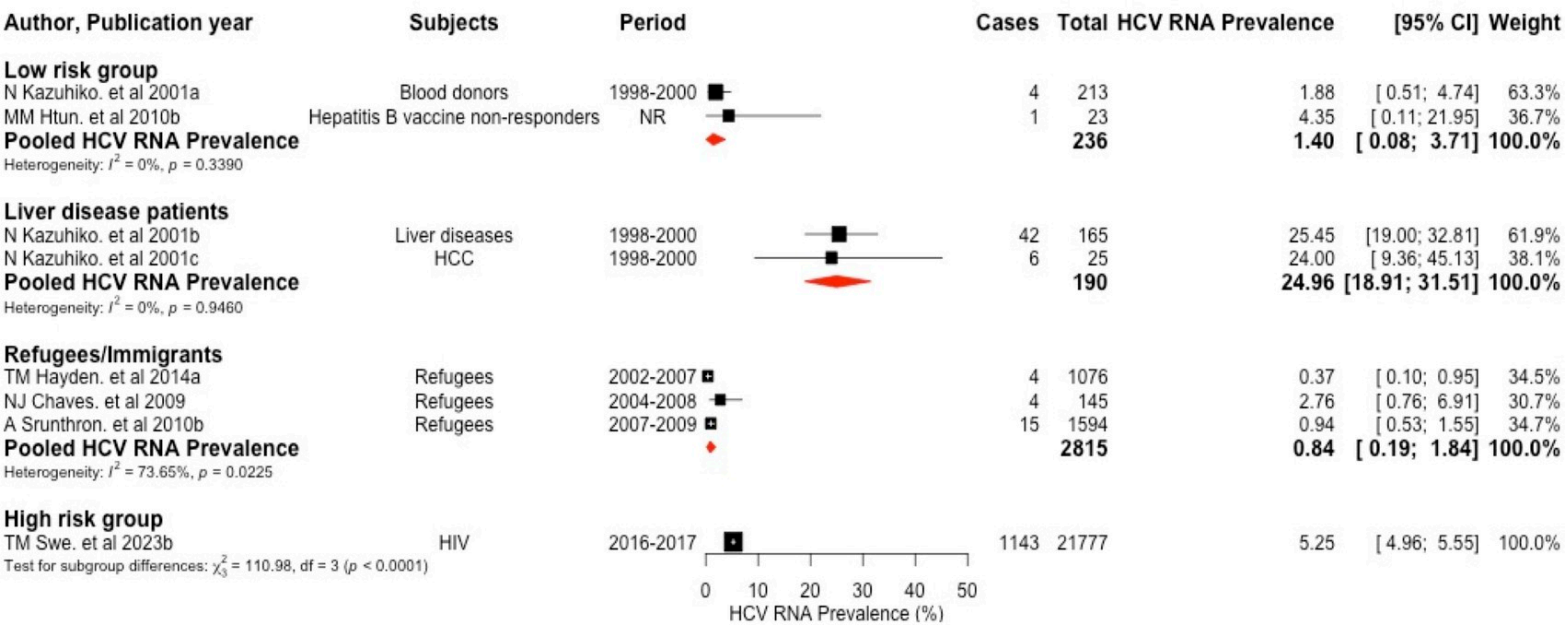
Forest plot of the pooled HCV RNA prevalence in Myanmar, using random effect. This figure shows the funnel plot of 7 studies and the pooled HCV RNA prevalence in sub-groups: low-risk group, liver disease patients, and refugees/immigrants. The diamond block represents for pooled prevalence and the rectangular block represents prevalence for each study.

Eight studies [8, 22, 28, 30, 32, 35] measured HCV RNA, and the positive rate ranged from 0.37% in refugees to 25.45% in liver disease patients. The pooled prevalence of HCV RNA was estimated at 1.40% (95%CI: 0.08–3.71, I^2^ = 0%, p = 0.3390) in low-risk group, 5.25% (95%CI: 4.96–5.55) in high-risk group, 24.96% (95%CI: 18.91–31.51, I^2^ = 0%, p = 0.0115) in patients with liver diseases and 0.84% (95%CI: 0.19–1.84, I^2^ = 73.65%, p = 0.0225) in refugees/immigrants (Fig 3).

### HCV genotypes

**Fig 4:**
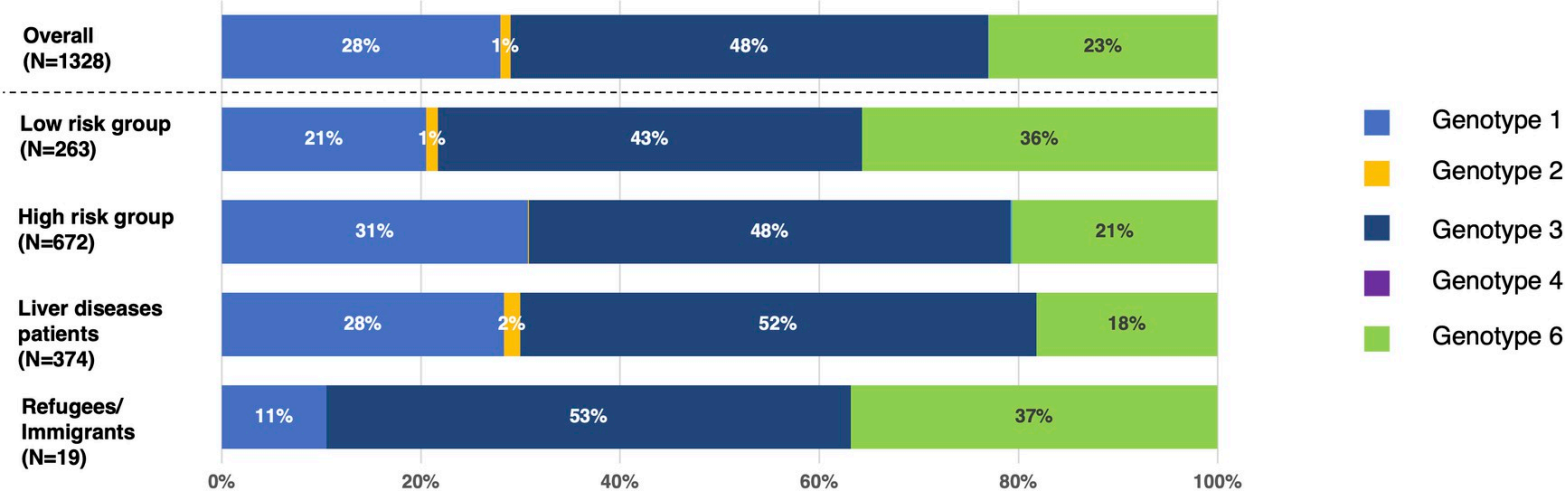
Distribution of HCV genotypes in Myanmar. This figure shows the HCV genotype distribution overall and by group of population in Myanmar. Each color represents each genotype: light blue for Genotype 1, orange for Genotype 2, dark blue for Genotype 3, and light green for Genotype 6.

**Fig 5:**
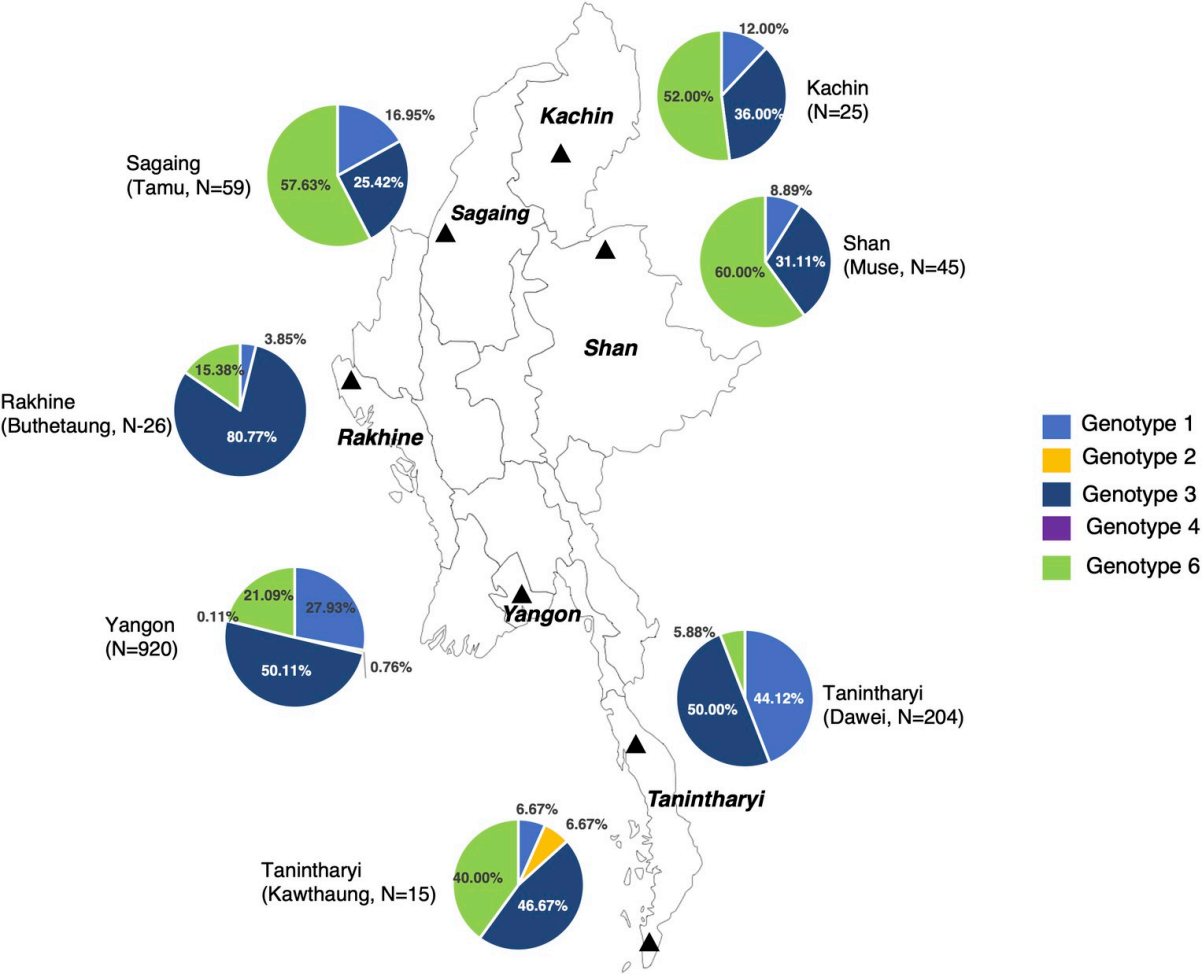
HCV genotypes distribution by study place in Myanmar (N = 1,294). This figure shows the analysis of HCV genotype distribution by region in Myanmar. Each pie chart represents each study place. Each color inside the pie chart represents each genotype: light blue for Genotype 1, orange for Genotype 2, dark blue for Genotype 3, and light green for Genotype 6.

The genotyping of the HCV was performed in 10 studies [8, 18, 21, 23, 28, 32, 35, 40, 41, 46]. Overall, HCV genotype 3 was most predominant (48%) followed by genotypes 1 (28%), 6 (23%), 2 (1%), and 4 (0.27%). By sub-group analysis, genotype 3 was predominant in all sub-groups of low-risk, high-risk, liver disease patients and refugees/immigrants (43%, 48%, 52%, and 53% respectively). The HCV genotype distribution pattern was similar between low-risk groups and refugees/immigrants: genotype 3 (43% vs. 53%) followed by 6 (36% vs. 37%) and 1 (21% vs. 11%). The HCV genotype distribution was the similar in high-risk groups and liver disease patients: genotype 3 (48% vs 52%) followed by 1 (31% vs. 28%) and 6 (21% vs. 18%). HCV genotype 2 was exclusively found in low-risk and liver disease patients (1% and 2% respectively) and only one case of genotype 4 was reported in a high-risk group (PLHIV) (Fig 4). The analysis of HCV genotype distribution by region showed that genotype 6 was predominant in the northern part of Myanmar whilst genotype 3 was predominant in the west and southern parts of Myanmar (Fig 5).

### Publication bias

**Fig 6:**
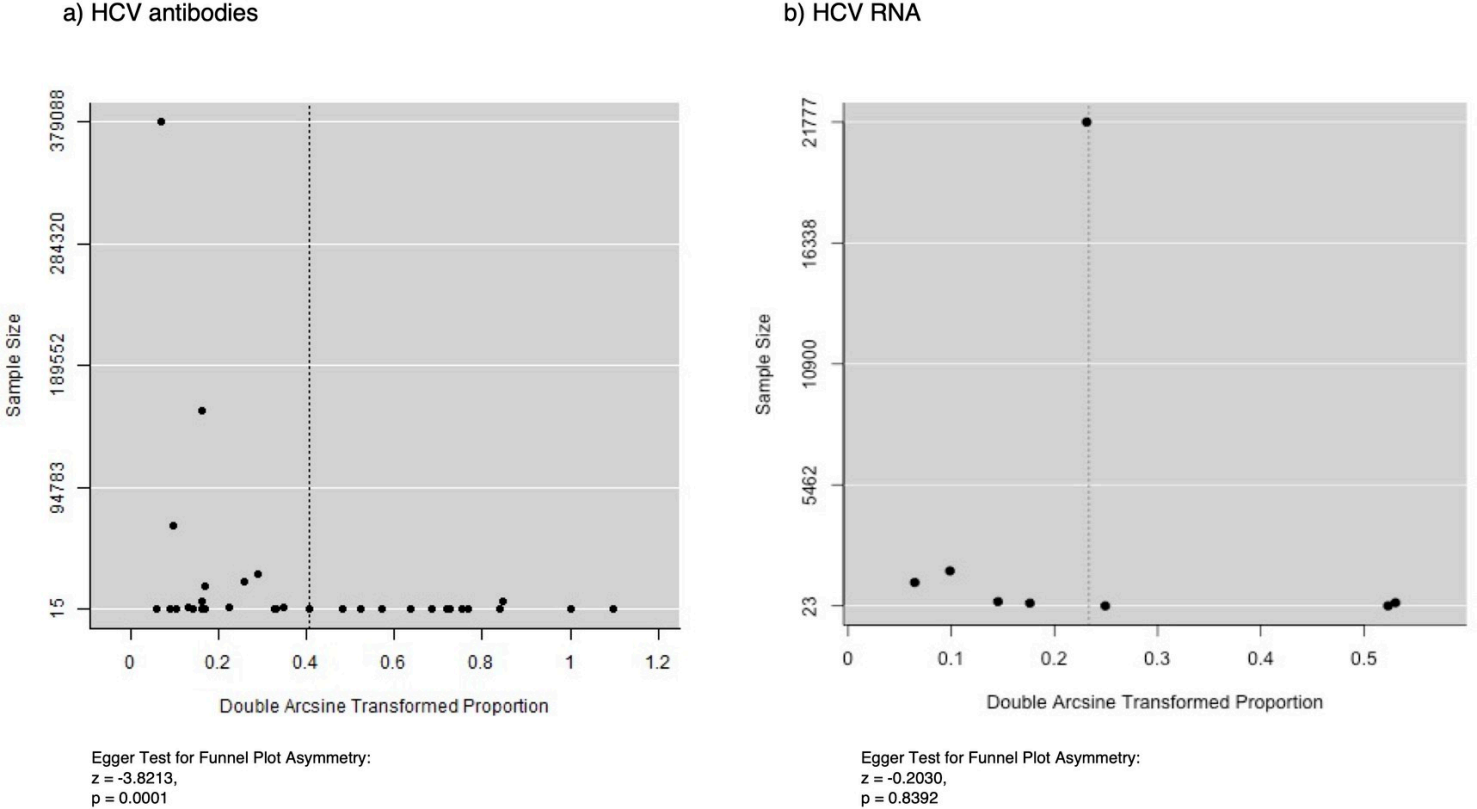
Funnel plot of HCV prevalence a) HCV antibodies and b) HCV RNA. Fig 6A and 6B show the forest plot for the assessment of publication bias among the recruited studies for estimating pooled HCV antibodies and HCV RNA prevalence. The dotted line represents the resultant pooled prevalence, and each black circle represents the prevalence of each study. The prevalence was plotted against the sample size.

Using the graphical assessment of the funnel plot, the publication bias was found in HCV seroprevalence studies, but there was no publication bias in HCV RNA prevalence studies. In the Egger tests for funnel plot asymmetry, the p-values were 0.0001 and 0.8392 respectively (Fig 6A and 6B).

## Discussion

This systematic review revealed Myanmar has the intermediate endemicity of HCV seroprevalence among low-risk groups including the general population. The analysis of HCV seroprevalence by sub-groups offered a more comprehensive understanding of the disease burden within each group in Myanmar. The disease spectrum differs by the characteristic of each group, and it is encountered to consider adopting effective countermeasures against HCV in Myanmar.

This meta-analysis revealed publication bias in HCV seroprevalence studies but not in HCV RNA prevalence studies. The presence of publication bias indicates a likelihood of overestimating seroprevalence rates due to the selective publication of studies with higher rates. Therefore, the pooled prevalence was not determined as a whole, and the participants were categorized into four groups: low-risk groups, high-risk groups, liver disease patients, and immigrants/refugees computed the pooled prevalence in each group using a random effect. This meta-analysis included the Burmese immigrants or refugees residing in other countries and it is believed that this group of the population reflects the disease burden of HCV among the general population in Myanmar.

HCV seroprevalence in low-risk groups and immigrants/refugees were 2.18% and 2.52% respectively. Despite the sensitivity analysis showing a slight increase in HCV antibody prevalence after removing the unpublished reports in low-risk groups, the change was not statistically significant. However, caution should be exercised when interpreting the pooled prevalence estimates. A significantly high level of heterogeneity was noticed in both groups (I^2^: 99.8% vs. 83.24%). Considering the prevalence of 2.18% in the general population, 1.18 million individuals were exposed to HCV infection across the country as of 2023 Myanmar’s population data [47]. In Myanmar, a nationwide study on the prevalence of HBV and HCV was conducted in 18 study sites in 2015 and reported that the prevalence of HBV and HCV was 6.5% and 2.7% respectively [10]. The pooled HCV seroprevalence among low-risk groups and immigrants/refugees in this meta-analysis is consistent with a 2015 nationwide study. The studies conducted from 2000 through 2017 among the abovementioned population groups likely do not reflect the current epidemiological situation of HCV among the general population in Myanmar.

The pooled HCV seroprevalence of 37.07% was found in high-risk groups which comprised of frequent blood recipients (Thalassemia etc.), hemodialysis patients, PWID, and PLHIV, all of whom were highly prone to HCV infection. A significant high level of heterogeneity was observed in this group with reported I^2^ at 99.8%. The reported studies were conducted from 1996 through 2018. The high HCV seroprevalence rates among PWID, ranging from 40% to 79%, highlight the critical importance of implementing targeted interventions although harm reduction strategies that have been introduced since 2005–2006 in Myanmar. Moreover, the HIV/HCV coinfection rate ranged from 5% to 22.8% and the risk was high in the PWID population of Myanmar (Odds Ratio: 20.1, 95% CI: 13.7 to 29.5) [48, 49] which highlights the interplay between these two infections and the need for integrated prevention and treatment approaches in PWID.

The pooled HCV seroprevalence among liver disease patients was calculated to be 33.84% with a reported medium heterogeneity of 65.13%. All the studies on liver disease patients were conducted between 1991 and 1998 and there was no report or studies after 2002. Most of the studies used convenient sampling methods for participant recruitment and the under or overestimation of HCV seroprevalence among this group cannot be ruled out. After two decades, the attribution of HBV and HCV etiology to liver disease including HCC might be changed and it needs to be evaluated again.

The decreasing trend of HCV seroprevalence among the low-risk group was found by a period of data collection. It can be explained by the installation of blood donor screening in 2000, the promotion of infection control including disposable syringe usage in health care facilities in the early 2000 and raising the awareness of viral hepatitis in 2011 with local and international non-governmental organizations’ involvement in viral hepatitis screening and health education.

HCV RNA positive rate was supposed to be low in low-risk and immigrants/refugee groups (1.4% vs 0.84%) assuming that approximately 50% of them were naturally resolved and the number of viremic cases was estimated at 0.7 million individuals in Myanmar. The high HCV RNA-positive rate was found in liver disease patients (24.96%) but its attribution to hepatocellular carcinoma is unknown.

There were no incidence studies on HCV infection in Myanmar and only a few of the included prevalence studies were conducted mostly in an urban area with representative samples of the target population and there were no studies or reports on HCV disease burden after the 2015 nationwide study. Furthermore, the coronavirus disease 2019 (COVID-19) pandemic together with the unstable political situation in 2021 delayed or interrupted the healthcare system in Myanmar which is threatening the infectious diseases control, countermeasures, and the road to elimination of viral hepatitis in Myanmar. A well-structured basic epidemiological study to examine the disease burden of HCV in Myanmar is recommended.

In Myanmar, the predominant HCV genotype was 3 (48%) followed by 1 (28%) and 6 (23%) overall. This information is significant in guiding HCV treatment strategies, as genotypes can impact disease progress, treatment response, and duration. For instance, genotype 3 is associated with a higher risk of developing severe liver injuries, such as cirrhosis and HCC [50]. The obvious geographical difference in genotype distribution was found across the country. In the northern region of Myanmar, which shares a border with China’s Yunnan province, a high prevalence of HCV genotype 6 was observed in the PWID population. Likewise, PWID in Yunnan province, HCV genotype 6 was found to be the predominant genotype. This finding suggests that there may be transmission of HCV among PWID populations occurring through the border regions between Myanmar and Yunnan province [51]. However, HCV genotype 3 was predominant in the southern part of Myanmar, and the southern east of Myanmar is bordered by Thailand where genotype 3 is reportedly predominant [52]. HCV genotype 3 was also predominant in the western part of Myanmar, which shares borders with India and Bangladesh. These neighboring countries also have a high prevalence of genotype 3 [53, 54]. Understanding the genotype distribution and its geographical difference revealed the potential route of transmission from which effective prevention and control strategies should be developed. The genotypic diversity observed in Myanmar serves as a valuable contribution to the overall understanding of HCV genotypes in the Southeast Asia region.

This study has several limitations. The significant heterogeneity among the included studies may introduce variations in the estimated prevalence rates. This heterogeneity can be attributed to differences in sampling methods, individual characteristics of the study population, involvement of small sample sizes in some studies, and sampling biases in each recruited study so that the over or underestimation of the prevalence cannot be excluded. Most of the included studies did not provide the study setting clearly so that the urban and rural areas cannot be categorized, but most studies were assumed to be conducted in urban settings so the study in rural settings is limited in Myanmar and the actual disease burden among rural population is unknown. Moreover, the study on HCV among not only key populations such as men who have sex with men (MSM), female sex workers (FSW), and people in prisons but also the health care professionals (HCP) and pregnant women are still unknown.

In conclusion, this systematic review and meta-analysis indicated that Myanmar has intermediate endemicity of HCV seroprevalence in the low-risk group with a decreasing trend over the past 20 years due to measures such as blood donor screening, infection control practices, and increased awareness through education programs. However, the findings highlight the need for further epidemiological studies to understand the actual disease burden and implement effective countermeasures, including screening, linkage to care, and accessibility to treatment, to achieve the WHO’s goal of HCV elimination by 2030.

## Supporting information

S1 Checklist Inclusivity in global research questionnaire. (DOCX)

S1 Appendix PRISMA checklist. (DOCX)

S1 Table Database search. (DOCX)

S2 Table Quality appraisal of included studies using the Joanna Briggs Institute checklist for prevalence studies. (DOCX)

## List of abbreviations

HCV: Hepatitis C virus
DAA: Direct-acting antivirus
RNA: Ribonucleic acid
WHO: World Health Organization
PRISMA: Preferred Reporting Items for Systematic Reviews and Meta-Analysis
PROSPERO: International Prospective Register of Systematic Reviews
INPLASY: International Platform of Registered Systematic Review and Meta-analysis Protocols
CI: Confidence Interval
MMA: Myanmar Medical Association
MLF: Myanmar Liver Foundation
MSF: Medecins Sans Frontieres
NHCP: National Hepatitis Control Program
SVR: Sustained virological response
PLHIV: People living with human immunodeficiency virus
PWID: People who inject drugs
HBV: Hepatitis B Virus
HCC: Hepatocellular carcinoma
HIV: Human immunodeficiency virus
MSM: Men having sex with men
FSW: Female sex workers
HCP: Health care professional
