# Supplementary material for: Intermediate hepatitis C virus (HCV) endemicity and its genotype distribution in Myanmar: A systematic review and meta-analysis
Source: PLoS One. 2024 Sep 19;19(9):e0307872. doi: 10.1371/journal.pone.0307872 (PMC11412534; doi:10.1371/journal.pone.0307872)
Supplement: S1 Table — (DOCX) [file pone.0307872.s003.docx]

**Table S1: Database search**

| **No** | **Database** | **Search query** | **Number of results** |
| --- | --- | --- | --- |
| **1** | **PubMed** | ("HEPATITIS C" OR "ANTI-HCV" OR “HCV-Ab” OR "HCV" OR “HCV GENOTYPE”) AND (“PREVALENCE” OR “SEROPREVALENCE”) AND ("MYANMAR" OR "BURMA") | 34 |
| **2** | **Web of Science** | (TS=HEPATITIS C OR TS=ANTI-HCV OR TS=HCV-Ab OR TS=HCV OR TS=HCV GENOTYPE) AND (TS=PREVALENCE OR TS=SEROPREVALENCE) AND (TS=MYANMAR OR TS=BURMA) | 44 |
| **3** | **Scopus** | (ALL (Hepatitis AND C) OR ALL (HCV) OR ALL (hep AND c) AND ALL (seroprevalence) OR ALL (prevalence) AND TITLE-ABS-KEY (Myanmar OR Burma)) | 48 |
|  |  |  |  |
