# Supplementary material for: Intermediate hepatitis C virus (HCV) endemicity and its genotype distribution in Myanmar: A systematic review and meta-analysis
Source: PLoS One. 2024 Sep 19;19(9):e0307872. doi: 10.1371/journal.pone.0307872 (PMC11412534; doi:10.1371/journal.pone.0307872)
Supplement: S2 Table — (DOCX) [file pone.0307872.s004.docx]

**Table S2: Quality appraisal of included studies using the Joanna Briggs Institute checklist for prevalence studies**

| **Author, Year of publication** | **Q1. Was the sample frame appropriate to address the target population?** | **Q2. Were study participants sampled in an appropriate way?** | **Q3. Was the sample size adequate?** | **Q4. Were the study subjects and the setting described in detail?** | **Q5. Was the data analysis conducted with sufficient coverage of the identified sample?** | **Q6. Were valid methods used for the identification of the condition?** | **Q7. Was the condition measured in a standard, reliable way for all participants?** | **Q8. Was there appropriate statistical analysis?** | **Q9. Was the response rate adequate, and if not, was the low response rate managed appropriately?** |
| --- | --- | --- | --- | --- | --- | --- | --- | --- | --- |
| KP Kyi, 1998 | Yes | Yes | No | Yes | Yes | Yes | Yes | Yes | Yes |
| M Khin, 2000 | Yes | Yes | Yes | Yes | Yes | Unclear | Unclear | Yes | Yes |
| O Shigeru, 2000a | Yes | Unclear | No | Yes | Yes | Yes | Yes | Yes | Yes |
| O Shigeru, 2000b | Yes | Unclear | No | Yes | Yes | Yes | Yes | Yes | Yes |
| O Shigeru, 2000c | Yes | Unclear | No | Yes | Yes | Yes | Yes | Yes | Yes |
| O Shigeru, 2000d | Yes | Unclear | No | Yes | Yes | Yes | Yes | Yes | Yes |
| N Win, 2000 | Yes | Unclear | No | Yes | Yes | Yes | Yes | Yes | Yes |
| N Kazuhiko, 2001a | Yes | Yes | Yes | Yes | Yes | Yes | Yes | Yes | Yes |
| N Kazuhiko, 2001b | Yes | Yes | Yes | Yes | Yes | Yes | Yes | Yes | Yes |
| N Kazuhiko, 2001c | Yes | Yes | Yes | Yes | Yes | Yes | Yes | Yes | Yes |
| T Kazuhisa, 2002 | Unclear | Unclear | No | Yes | Yes | Yes | Yes | Yes | Yes |
| T Kazuhisa, 2002 | Unclear | Unclear | No | Yes | Yes | Yes | Yes | Yes | Yes |
| KP Kyi, 2002a | Yes | Yes | Yes | Yes | Yes | Yes | Yes | Yes | Yes |
| KP Kyi, 2002b | Yes | Unclear | Yes | Yes | Yes | Yes | Yes | Yes | Yes |
| M Khin, 2003 | Unclear | Unclear | Yes | Yes | Yes | Unclear | Unclear | Yes | Yes |
| AA Lwin, 2007 | Yes | Yes | Yes | Yes | Yes | Yes | Yes | Yes | Yes |
| A Denburg, 2007 | Yes | Yes | No | Yes | Yes | Unclear | Unclear | Yes | Yes |
| A Thu, 2008 | Yes | Yes | No | Yes | Yes | Yes | Yes | Yes | Yes |
| NJ Chaves, 2009 | Yes | Yes | No | Yes | Yes | Yes | Yes | Yes | Yes |
| M Khin, 2010 | Yes | Yes | Yes | Yes | Yes | Yes | Yes | Yes | Yes |
| A Srunthron, 2010a | Yes | Yes | Yes | Yes | Yes | Yes | Yes | Yes | Yes |
| **Author, Year of publication** | **Q1. Was the sample frame appropriate to address the target population?** | **Q2. Were study participants sampled in an appropriate way?** | **Q3. Was the sample size adequate?** | **Q4. Were the study subjects and the setting described in detail?** | **Q5. Was the data analysis conducted with sufficient coverage of the identified sample?** | **Q6. Were valid methods used for the identification of the condition?** | **Q7. Was the condition measured in a standard, reliable way for all participants?** | **Q8. Was there appropriate statistical analysis?** | **Q9. Was the response rate adequate, and if not, was the low response rate managed appropriately?** |
|  |  |  |  |  |  |  |  |  |  |
| A Srunthron, 2010b | Yes | Yes | Yes | Yes | Yes | Yes | Yes | Yes | Yes |
| MM Htun, 2010a | Unclear | Yes | No | Yes | Yes | Yes | Yes | Yes | Yes |
| MM Htun, 2010b | Unclear | Yes | No | Yes | Yes | Yes | Yes | Yes | Yes |
| YH Zhou, 2011 | Yes | Yes | No | Yes | Yes | Yes | Yes | Yes | Yes |
| GA Paxton, 2012 | Yes | Yes | Yes | Yes | Yes | Unclear | Unclear | Yes | Yes |
| TM Hayden, 2014 | Yes | Yes | Yes | Yes | Yes | Yes | Yes | Yes | Yes |
| MOH, 2015 | Yes | Yes | Yes | Yes | Yes | Yes | Yes | Yes | Yes |
| AA Lwin, 2017 | Yes | Yes | Yes | Yes | Yes | Yes | Yes | Yes | Yes |
| AA Lwin, 2018 | Unclear | Unclear | No | Yes | Yes | Yes | Yes | Yes | Yes |
| NS Aye, 2018 | Yes | Unclear | Yes | Yes | Yes | Yes | Yes | Yes | Yes |
| CC Ngo, 2018 | Yes | Yes | No | Yes | Yes | Unclear | Unclear | Yes | Yes |
| HS Juon, 2019 | Yes | Yes | Yes | Yes | Yes | Unclear | Unclear | Yes | Yes |
| M Ye, 2019 | Yes | Unclear | No | Yes | Yes | Yes | Yes | Yes | Yes |
| Myanmar IBBS, 2019 | Yes | Yes | Yes | Yes | Yes | Yes | Yes | Yes | Yes |
| KT Nyunt, 2019 | Unclear | Unclear | No | Yes | Yes | Yes | Yes | Yes | Yes |
| TS Win, 2020 | Yes | Unclear | No | Yes | Yes | Yes | Yes | Yes | Yes |
| NTT Kyaw, 2022 | Yes | Yes | Yes | Yes | Yes | Yes | Yes | Yes | Yes |
| K Urban, 2023 | Yes | Yes | Yes | Yes | Yes | Unclear | Unclear | Yes | Yes |
| TM Swe, 2023a | Yes | Yes | Yes | Yes | Yes | Yes | Yes | Yes | Yes |
| TM Swe, 2023b | Yes | Yes | Yes | Yes | Yes | Yes | Yes | Yes | Yes |
