# Supplementary material for: Intermediate hepatitis C virus (HCV) endemicity and its genotype distribution in Myanmar: A systematic review and meta-analysis
Source: PLoS One. 2024 Sep 19;19(9):e0307872. doi: 10.1371/journal.pone.0307872 (PMC11412534; doi:10.1371/journal.pone.0307872)
Supplement: Attachment — Submitted filename: PONE-D-24-10345 Revised Dr Zeukoo.pdf [file pone.0307872.s005.pdf]

PLOS ONE

Intermediate hepatitis C virus (HCV) endemicity and its genotype distribution in Myanmar: A systematic review and meta-analysis

--Manuscript Draft--

Manuscript Number:	PONE-D-24-10345
Article Type:	Research Article
Full Title:	Intermediate hepatitis C virus (HCV) endemicity and its genotype distribution in Myanmar: A systematic review and meta-analysis
Short Title:	Hepatitis C in Myanmar
Corresponding Author:	Junko Tanaka, Ph.D.<br>Hiroshima University Faculty of Medicine Graduate School of Biomedical and Health Sciences: Hiroshima Daigaku Igakubu Daigakuin Ishiyaku Hokengaku Kenkyuka<br>Hiroshima, JAPAN
Keywords:	Seroprevalence; Hepatitis; HCV; HCV-Ab; HCV RNA; HCV genotype; Myanmar; Burma; Systematic review; Meta-analysis.
Abstract:	Background : This study estimated HCV antibodies and ribonucleic acid (RNA) prevalence and genotype distribution in various populations inMyanmar between 1990 and 2023.<br>Methods: A systematic search in PubMed, Web of Science, Scopus, and local journals, identified studies on HCV prevalence or genotypes for inclusion. All relevant data were extracted, and study populations were categorized into low-risk, high-risk, liver disease patients, and refugees outside the country. The pooled prevalence was performed by Dersimonian and Laird method using the R program. The publication bias was shown by funnel plot, the Egger test was used to assess the symmetry of the plot, and the heterogeneity was examined by the Cochran Q test and I2 index.<br>Results: Out of a total of 135 reports screened for eligibility, 35 reports comprising 51 studies were included in which 33 studies provided data on HCV seroprevalence in 685,403 individuals, 8 studies reported HCV RNA prevalence in 25,018 individuals, and 10 studies examined HCV genotypes in 1,845 individuals. The pooled seroprevalence of HCV among low-risk, high-risk, liver disease patients and refugees were 2.18%, 37.07%, 33.84%, and 2.52% respectively. HCV RNA-positive rates in low-risk, high-risk, liver disease patients and refugees were 1.40%, 5.25%, 24.96%, and 0.84% respectively. Seroprevalence studies showed publication bias (Egger test, p=0.0001), RNA studies did not (Egger test, p=0.8392). HCV genotype 3 was predominant in all sub-groups in Myanmar.<br>Conclusion: This systematic review and meta-analysis indicated that Myanmar has intermediate endemicity of HCV seroprevalence in the low-risk group with a decreasing trend over the past 20 years due to measures such as blood donor screening, infection control practices, and increased awareness through education programs. However, the findings highlight the need for further epidemiological studies to understand the actual disease burden and implement effective countermeasures to achieve the WHO's goal of HCV elimination by 2030.
Order of Authors:	Zayar Phyo
	Ko Ko
	Serge Ouoba
	Aya Sugiyama
	Ulugbek Khudayberdievich Mirzaev
	Golda Ataa Akuffo
	Chanroth Chhoung
	Tomoyuki Akita
	Junko Tanaka, Ph.D.
Additional Information:	

Powered by Editorial Manager® and ProduXion Manager® from Aries Systems Corporation

Question	Response
**Financial Disclosure**<br><br>Enter a financial disclosure statement that describes the sources of funding for the work included in this submission. Review the submission guidelines for detailed requirements. View published research articles from *PLOS ONE* for specific examples.<br><br>This statement is required for submission and **will appear in the published article** if the submission is accepted. Please make sure it is accurate.<br><br>**Funded studies**<br>Enter a statement with the following details:<br>• Initials of the authors who received each award<br>• Grant numbers awarded to each author<br>• The full name of each funder<br>• URL of each funder website<br>• Did the sponsors or funders play any role in the study design, data collection and analysis, decision to publish, or preparation of the manuscript?<br><br>Did you receive funding for this work?	Yes
Please add funding details.<br>as follow-up to "**Financial Disclosure**<br><br>Enter a financial disclosure statement that describes the sources of funding for the work included in this submission. Review the submission guidelines for detailed requirements. View published research articles from *PLOS ONE* for specific examples.<br><br>This statement is required for submission and **will appear in the published article** if the submission is accepted. Please make sure it is accurate.	This work was supported by Japan’s Ministry of Health, Labour and Welfare (MHLW) Policy Research for Hepatitis Measures Program Grant Number JPMH22HC1001.

**Funded studies**<br>Enter a statement with the following details:<br>• Initials of the authors who received each award<br>• Grant numbers awarded to each author<br>• The full name of each funder<br>• URL of each funder website<br>• Did the sponsors or funders play any role in the study design, data collection and analysis, decision to publish, or preparation of the manuscript?<br><br>Did you receive funding for this work?"	
Please select the country of your main research funder (please select carefully as in some cases this is used in fee calculation).<br>as follow-up to "**Financial Disclosure**<br><br>Enter a financial disclosure statement that describes the sources of funding for the work included in this submission. Review the submission guidelines for detailed requirements. View published research articles from *PLOS ONE* for specific examples.<br><br>This statement is required for submission and **will appear in the published article** if the submission is accepted. Please make sure it is accurate.<br><br>**Funded studies**<br>Enter a statement with the following details:<br>• Initials of the authors who received each award<br>• Grant numbers awarded to each author<br>• The full name of each funder<br>• URL of each funder website<br>• Did the sponsors or funders play any role in the study design, data collection and analysis, decision to publish, or preparation of the manuscript?<br><br>Did you receive funding for this work?"	JAPAN - JP
**Competing Interests**	The authors declare no conflict of interest.

Use the instructions below to enter a competing interest statement for this submission. On behalf of all authors, disclose any competing interests that could be perceived to bias this work—acknowledging all financial support and any other relevant financial or non-financial competing interests.<br><br>This statement is required for submission and **will appear in the published article** if the submission is accepted. Please make sure it is accurate and that any funding sources listed in your Funding Information later in the submission form are also declared in your Financial Disclosure statement.<br><br>View published research articles from *PLOS ONE* for specific examples.<br><br>**NO authors have competing interests**<br><br>Enter: *The authors have declared that no competing interests exist.*<br><br>**Authors with competing interests**<br><br>Enter competing interest details beginning with this statement:<br><br>*I have read the journal's policy and the authors of this manuscript have the following competing interests: [insert competing interests here]*<br><br>* typeset	
**Ethics Statement**<br><br>Enter an ethics statement for this submission. This statement is required if the study involved:<br><br>• Human participants<br>• Human specimens or tissue<br>• Vertebrate animals or cephalopods<br>• Vertebrate embryos or tissues	Not applicable.

Powered by Editorial Manager® and ProduXion Manager® from Aries Systems Corporation

• Field research<br><br>Write "N/A" if the submission does not require an ethics statement.<br><br>General guidance is provided below. Consult the submission guidelines for detailed instructions. **Make sure that all information entered here is included in the Methods section of the manuscript.**	

**Format for specific study types**<br><br>**Human Subject Research (involving human participants and/or tissue)**<br>• Give the name of the institutional review board or ethics committee that approved the study<br>• Include the approval number and/or a statement indicating approval of this research<br>• Indicate the form of consent obtained (written/oral) or the reason that consent was not obtained (e.g. the data were analyzed anonymously)<br><br>**Animal Research (involving vertebrate animals, embryos or tissues)**<br>• Provide the name of the Institutional Animal Care and Use Committee (IACUC) or other relevant ethics board that reviewed the study protocol, and indicate whether they approved this research or granted a formal waiver of ethical approval<br>• Include an approval number if one was obtained<br>• If the study involved *non-human primates*, add *additional details* about animal welfare and steps taken to ameliorate suffering<br>• If anesthesia, euthanasia, or any kind of animal sacrifice is part of the study, include briefly which substances and/or methods were applied<br><br>**Field Research**<br><br>Include the following details if this study involves the collection of plant, animal, or other materials from a natural setting:<br>• Field permit number<br>• Name of the institution or relevant body that granted permission	
**Data Availability**<br><br>Authors are required to make all data underlying the findings described fully available, without restriction, and from the time of publication. PLOS allows rare exceptions to address legal and ethical concerns. See the PLOS Data Policy and FAQ for detailed information.	Yes - all data are fully available without restriction

A Data Availability Statement describing where the data can be found is required at submission. Your answers to this question constitute the Data Availability Statement and **will be published in the article**, if accepted.<br><br>**Important:** Stating 'data available on request from the author' is not sufficient. If your data are only available upon request, select 'No' for the first question and explain your exceptional situation in the text box.<br><br>Do the authors confirm that all data underlying the findings described in their manuscript are fully available without restriction?	
**Describe where the data may be found in full sentences. If you are copying our sample text, replace any instances of XXX with the appropriate details.**<br><br>• If the data are **held or will be held in a public repository**, include URLs, accession numbers or DOIs. If this information will only be available after acceptance, indicate this by ticking the box below. For example: *All XXX files are available from the XXX database (accession number(s) XXX, XXX.).*<br>• If the data are all contained **within the manuscript and/or Supporting Information files**, enter the following: *All relevant data are within the manuscript and its Supporting Information files.*<br>• If neither of these applies but you are able to provide **details of access elsewhere**, with or without limitations, please do so. For example:<br><br>*Data cannot be shared publicly because of [XXX]. Data are available from the XXX Institutional Data Access / Ethics Committee (contact via XXX) for researchers who meet the criteria for access to confidential data.*<br><br>*The data underlying the results presented in the study are available from (include the name of the third party*	All relevant data are within the manuscript and its Supporting Information files.

and contact information or URL).<br>• This text is appropriate if the data are owned by a third party and authors do not have permission to share the data.<br><br>* typeset	
Additional data availability information:	

Manuscript

Click here to access/download;Manuscript;20240313 Manuscript and Tables_HCV_PLOS ONE_V2.docx

Intermediate hepatitis C virus (HCV) endemicity and its genotype distribution in Myanmar: A systematic review and meta-analysis

Zayar Phyo[1,2], Ko Ko[1,2], Serge Ouoba[1,2,3], Aya Sugiyama[1,2], Ulugbek Khudayberdievich Mirzaev[1,2,4], Golda Ataa Akuffo[1,2], Chanroth Chhoung[1,2], Tomoyuki Akita[1,2], Junko Tanaka[1,2*]

[1]Department of Epidemiology, Infectious Disease Control and Prevention, Graduate School of Biomedical and Health Science, Hiroshima University, Hiroshima, Japan

[2]Project Research Center for Epidemiology and Prevention of viral hepatitis and hepatocellular carcinoma, Hiroshima University

[3]Unité de Recherche Clinique de Nanoro (URCN), Institut de Recherche en Science de la Santé (IRSS), Nanoro, Burkina Faso

[4]Department of Hepatology, Scientific Research Institute of Virology, Tashkent, Uzbekistan

Short title: Hepatitis C in Myanmar

*Corresponding Author

Address for correspondence and reprint requests:

Junko Tanaka, PhD

Department of Epidemiology, Infectious Disease Control and Prevention, Graduate School of Biomedical and Health Sciences Hiroshima University, Hiroshima, Japan

1-2-3, Kasumi, Minami-ku, Hiroshima, 734-8551, Japan

Tel: +81-82-257-5160

Fax: +81-82-257-5164

Email: jun-tanaka@hiroshima-u.ac.jp

Abstract

Background: This study estimated HCV antibodies and ribonucleic acid (RNA) prevalence and genotype distribution in various populations in Myanmar between 1990 and 2023.

Methods: A systematic search in PubMed, Web of Science, Scopus, and local journals, identified studies on HCV prevalence or genotypes for inclusion. All relevant data were extracted, and study populations were categorized into low-risk, high-risk, liver disease patients, and refugees outside the country. The pooled prevalence was performed by Dersimonian and Laird method using the R program. The publication bias was shown by funnel plot, the Egger test was used to assess the symmetry of the plot, and the heterogeneity was examined by the Cochran Q test and I^2 index.

Results: Out of a total of 135 reports screened for eligibility, 35 reports comprising 51 studies were included in which 33 studies provided data on HCV seroprevalence in 685,403 individuals, 8 studies reported HCV RNA prevalence in 25,018 individuals, and 10 studies examined HCV genotypes in 1,845 individuals. The pooled seroprevalence of HCV among low-risk, high-risk, liver disease patients and refugees were 2.18%, 37.07%, 33.84%, and 2.52% respectively. HCV RNA-positive rates in low-risk, high-risk, liver disease patients and refugees were 1.40%, 5.25%, 24.96%, and 0.84% respectively. Seroprevalence studies showed publication bias (Egger test, p=0.0001), RNA studies did not (Egger test, p=0.8392). HCV genotype 3 was predominant in all sub-groups in Myanmar.

Conclusion: This systematic review and meta-analysis indicated that Myanmar has intermediate endemicity of HCV seroprevalence in the low-risk group with a decreasing trend over the past 20 years due to measures such as blood donor screening, infection control practices, and increased awareness through education programs. However, the findings highlight the need for further epidemiological studies to understand the actual disease burden and implement effective countermeasures to achieve the WHO's goal of HCV elimination by 2030.

Keywords: Seroprevalence, Hepatitis, HCV, HCV-Ab, HCV RNA, HCV genotype, Myanmar, Burma, Systematic review, Meta-analysis.

Background

Globally, HCV infection affects 58 million people and annually new infections are estimated to be around 1.5 million(1). World Health Organization (WHO) reported that 20% of HCV-infected people had been diagnosed and a huge proportion of infected people remained undiagnosed because of lack of access to affordable testing(2). The imperceptible barriers were still present in line with the elimination of viral hepatitis as a global action, rising as a public health concern. In 2020, the WHO Southeast Asia region had an estimated 10 million people living with chronic hepatitis C with an attributable 40,938 deaths annually and only less than 1% of viremic diagnosed patients had been initiated treatment(3).

In Myanmar, liver-related research was started in 1972 and the liver research unit was established in 1973. The blood donor screening for HCV antibodies was started in 2000 and the awareness on prevention of viral hepatitis was enhanced in 2011. In 2013 and 2014, the Gastro-Intestinal and Liver Society of Myanmar Medical Association (MMA), a non-profit local organization developed the Viral Hepatitis Clinical Management Guidelines. After the establishment of the National Hepatitis Control Program (NHCP) in November 2014, the prevention, diagnosis, and treatment of viral hepatitis have been enhanced in Myanmar following the WHO's 2014 resolution strategies. Later the NHCP adopted the National Strategic Plan (2016-2020)(4) adhering to the world's vision to halt the transmission of viral hepatitis and ensuring everyone living with viral hepatitis has access to safe, affordable, and effective care and treatment. National Simplified Treatment Guideline for HCV infection was released in February 2017(5) and then direct-acting antivirals (DAAs) were used for the treatment of HCV infection.

Before 2017, no national program or strategic plan was providing free-of-charge treatment of HCV although the testing of HCV antibodies and HCV RNA was expensively available in private hospitals and laboratories. With the widespread availability of direct-acting antivirals (DAAs) which have reported fewer side effects with a high success rate (>90% sustained virologic response: SVR), the Government of Myanmar initiated the testing and the free-of-charge antiviral treatment for HCV in seven public hospitals in 2017 which has been denoted as a very first country providing free-of-charge HCV treatment in South-East Asia region. In Myanmar, various local and international non-governmental organizations have taken part in the efforts to eliminate HCV(6). One prominent organization involved in this endeavor is the Myanmar Liver Foundation (MLF), which has been offering anti-HCV treatment to patients with chronic liver diseases since 2017 through their Than Sitt Charity Clinics. Additionally, Medecins Sans Frontieres (MSF) has been providing anti-HCV treatment specifically to individuals

who are co-infected with HIV since 2017. Another organization actively engaged in combating HCV is the Burnet Institute of Myanmar. Since 2019, they have been delivering HCV treatment not only to the general population but also to people who inject drugs (PWID)(6).

In Myanmar, an estimated nationwide prevalence of HCV antibodies was 2.65%(7) in 2015 suggesting that 1.4 million people are exposed to HCV infection. Considering that 30% of infected persons spontaneously clear the virus within 6 months of infection without treatment(1), the total number of chronic HCV infections was estimated to be 0.9 million. From 1996 through 2017, some studies reported the prevalence of HCV antibodies ranging from the lowest of 0.50% among blood donors (unpublished study) to the highest of 79.19%(8) among PWID. Most of the studies are conducted in a convenient sampling strategy so that the external validation and representativeness of the target population are questionable.

Moreover, no studies related to the burden of HCV infection in Myanmar were found after 2018. Additionally, the ongoing political situation since 2021 disrupted the existing healthcare system and interrupted all the strategic plans that had been developed before. Hence, a comprehensive analysis of data spanning over three decades is needed to identify the situation of HCV infection in Myanmar and its potential impact on public health. Furthermore, by examining the distribution of HCV genotypes, the genetic diversity of the virus circulating among different populations can be elucidated. To provide the baseline epidemiological data on the disease burden of HCV infection in specific populations and the detailed characteristics in Myanmar, this study aimed to estimate the prevalence of HCV antibodies and HCV RNA and the distribution of HCV genotypes in various populations in Myanmar between 1990 and 2023.

Method

Study design and guidelines

This systematic review was conducted following the 2020 Preferred Reporting Items for Systematic Reviews and Meta-Analysis (PRISMA) guidelines(9) (Additional file 1).

Data source and study selection

To identify relevant records, we conducted searches in PubMed, Web of Science, and Scopus. The eligible studies included in our review were conducted between January 1990 and May 2023, reporting the prevalence of HCV antibodies, HCV RNA, and/or HCV genotype with exact sample sizes and the number

of positive cases. There were no restrictions on the type of study, and the subjects could be Myanmar citizens living inside or outside the country. However, clinical research or studies focusing on the prognosis of liver diseases in HCV-infected patients were excluded. Other factors such as gender, age, work nature, and place of living did not affect the eligibility. Only reports in English were considered, and manual searches were conducted in The Myanmar Health Sciences Research Journal, Google Scholar, reference lists of eligible reports, and government reports. The last search was conducted on May 31, 2023, and duplicate records were removed using the Rayyan – Intelligent Systematic Review tool. Two independent investigators (ZP and KK) screened the records based on titles and abstracts, and relevant reports were retained for full-text review. In cases of disagreements, a third investigator (JT) made the final decision.

Data extraction

All relevant raw data, including authors' names, year of publication, study design, sampling method, study period, characteristics of study subjects (age, sex, location, comorbidities), sample size, number of participants positive for HCV antibodies/HCV RNA/HCV genotype, and type of biological assay, were extracted from each recruited study using Microsoft Excel 2010. The data were counter-checked by a second author. In this study, a "report" refers to any document mentioning HCV prevalence/genotype data, while a "study" pertains to the measurement of HCV antibodies/HCV RNA/HCV genotype in a specific population. Consequently, one report may describe multiple studies, and data were extracted separately for each study. In cases where study subjects were found to overlap in two or more different studies, only one publication was included.

Quality Assessment of the Included Studies

The methodological quality of the prevalence studies was assessed using the Joanna Briggs Institute checklist for prevalence studies(10) (Additional file 2: Table S2). The review process unified the minimum acceptable information for each variable. The minimum sample size for prevalence estimation was set at 312 subjects, calculated based on an assumed seroprevalence of 2.65%(7), a precision of 1.8%, and a confidence level of 95%. The sampling procedure of each study was examined in detail, and studies employing probabilistic sampling were considered representative of the target population. The validity of seroprevalence and genotype data relied on whether the diagnostics were based on standard procedures and methods.

Figure 1: PRISMA showing selection of articles for meta-analysis of HCV in Myanmar

Statistical Analysis

The study populations were categorized into four groups based on the characteristics of subjects: (1) low-risk group, (2) high-risk group, (3) liver disease patients, and (4) refugees/immigrants. The low-risk group included the general population, blood donors, and hepatitis B virus (HBV) vaccine non-responders. The high-risk group contained people living with human immunodeficiency virus (PLHIV), PWID, patients who received blood transfusions, and hemodialysis patients. The other two groups were simply categorized as liver disease patients and refugees/immigrants. The statistical analysis was conducted using the 'meta' and 'metafor' packages in R programming. To accommodate small proportions, the data were transformed using the Freeman-Tukey double arcsine method. The meta-analysis was performed using the Dersimonian and Laird method, which utilizes a random-effects model, and the results were presented in a forest plot. Confidence intervals (CI) for the proportions of individual studies were calculated using the Clopper-Pearson method. To evaluate heterogeneity, the Cochran Q test was employed, and the degree of heterogeneity was quantified using the I^2 index. Heterogeneity was considered significant if the p-value of the Cochran Q test was less than 0.05. The high, medium, or low level of heterogeneity was identified by the I^2 index thresholds of 75%, 50%, and 25% respectively(11). To assess publication bias, a funnel plot of the transformed proportion against the sample size was generated(12). The symmetry of the plot was evaluated using the Egger test, with significance determined at a threshold of $p < 0.1$.

Results

Search results

A total of 126 articles were identified through the database search. Nine articles from the Myanmar Health Sciences Research Journal and government reports were manually added. After removing the duplication and irrelevant articles, 85 articles were screened by their abstracts and 35 articles were excluded under strict exclusion criteria. And then, 50 articles were accessed for full text and carefully reviewed for its eligibility. Finally, 35 articles met the eligible criteria and were inc[illegible]d in estimating the prevalence of HCV antibodies and HCV RNA and HCV genotype distribution in Myanmar (Figure 1). Some studies indicated the exact number of males and females or the place from which the samples were taken and also stated the age-specific number of samples and number of positive participants and corresponding prevalence, but most of the studies had lack of such information and it was quite difficult to divide the study subjects by their sex, age, and place of living. In terms of time series

for included reports, 5, 9, and 13 reports were conducted during 1990-2000, 2001-2010, and 2011-2020 respectively but 8 reports did not describe the study year(13-19) (Table 1).

Table 1: Characteristics of included studies reporting the prevalence of HCV and genotype distribution in Myanmar between 1990 and 2023

First author, year of publication	Study design	Sampling method	Data collection period	Study subjects	Mean age or range (in years)	Sample size	Number of HCV-Ab positive	Number of HCV RNA-positive	Number of amplified samples	Assay for HCV-Ab/RNA testing
J Mellor, 1996(13)	C	Convenient	NR	Blood donors	NR	8	-	-	8	-
KP Kyi, 1998(37)	CS	Consecutive	1991	HCC	20-89	40	14	-	-	ELISA
M Khin, 2000†	CS	Convenience	NR	General population	1-74	569	16	-	-	NR
O Shigeru, 2000a(38)	CS	NR	1996-1998	Thalassemia	7±3.5	15	7	-	-	ELISA
O Shigeru, 2000b(38)	CS	NR	1996-1998	Blood receipts	20±9	18	10	-	-	ELISA
O Shigeru, 2000c(38)	CS	NR	1996-1998	Liver diseases*	3-60	74	32	-	-	ELISA
O Shigeru, 2000d(38)	CS	NR	1996-1998	HCC	47±12	28	8	-	-	ELISA
N Win, 2000(39)	CS	NR	1996	HCC	23-70	16	7	-	-	ELISA
N Kazuhiko, 2001a(21)	CS	Convenience	1998-2000	Blood donors	7-80	213	-	4	-	PCR
N Kazuhiko, 2001bd(21)	CS	Convenience	1998-2000	Liver diseases*	7-80	165	-	42	24	PCR
N Kazuhiko, 2001c(21)	CS	Convenience	1998-2000	HCC	7-80	25	-	6	-	PCR

First author, year of publication	Study design	Sampling method	Data collection period	Study subjects	Mean age or range (in years)	Sample size	Number of HCV-Ab positive	Number of HCV RNA-positive	Number of amplified samples	Assay for HCV-Ab/RNA testing
T Kazuhisa, 2002a(15)	CS	NR	NR	Liver diseases*	NR	29	14	-	-	IRMA
T Kazuhisa, 2002b(15)	CS	NR	NR	HCC	NR	20	4	-	-	IRMA
KP Kyi, 2002a(14)	CS	Consecutive	NR	General population	NR	362	9	-	-	Immunoblot
KP Kyi, 2002b(14)	CS	Convenience	NR	Liver diseases*	NR	379	95	-	-	Immunoblot
M Khin, 2003[†]	CS	Consecutive	2000-2004	Blood donors	NR	154,161	4,008	-	-	NR
T Shinji, 2004(15)	RC	Convenient	2000	Blood donors	18-51	201	-	-	110	-
AA Lwin, 2007ab(16)	CS	Convenience	NR	General population	1-75	1,333	154	-	145	PA
A Denburg, 2007(40)	C	Consecutive	2006	Refugees	NR	68	0	-	-	NR
A Thu, 2008(8)	CS	Convenience	2007-2008	PWID	29.49±6.3	298	236	-	-	Rapid test
NJ Chaves, 2009(22)	RC	Consecutive	2004-2008	Refugees	16-86	145	-	4	-	PCR
M Khin, 2010(20)	CS	Consecutive	2005-2007	Blood donors	18-60	65,237	621	-	-	Rapid test
A Srunthron, 2010abc(25)	CS	Consecutive	2007-2009	Immigrants	15-60	1,594	27	15	15	ELISA (HCV-Ab)

										PCR (HCV RNA)
MM Htun, 2010ab(17)	CS	Consecutive	NR	HBV vaccine non-responders	NR	23	0	1	-	PA (HCV-Ab)<br>PCR (HCV RNA)
YH Zhou, 2011(41)	CS	Convenience	2005-2007	PWID	31.8±9.8	318	153	-	-	ELISA
GA Paxton, 2012(42)	CS	Consecutive	2007-2009	Refugees	0.5-87	519	10	-	-	NR
TM Hayden, 2014ab(23)	CS	Convenience	2002-2007	Refugees	15-95	1,076	-	4	4	PCR
MOH Myanmar, 2015[†]	CS	Consecutive	2015	Blood donors	15-80	379,088	1,895	-	-	Rapid test
C Naing, 2015(18)	RC	Convenient	NR	Chronic hepatitis C	47.1±11.6	362	-	-	350	-

**First author, year of publication**	**Study design**	**Sampling method**	**Data collection period**	**Study subjects**	**Mean age or range (in years)**	**Sample size**	**Number of HCV-Ab positive**	**Number of HCV RNA-positive**	**Number of amplified samples**	**Assay for HCV-Ab/RNA testing**
AA Lwin, 2017(7)	CS	Random	2015	General population	48 ± 14.6	5,547	147	-	-	ICT
AA Lwin, 2018(43)	CS	NR	2016	Haemodialysis patients	20-40	111	17	-	-	ICT
NS Aye, 2018(44)	RC	NR	2015-2017	PWID	24-34	531	376	-	-	Rapid test
CC Ngo, 2018(45)	RC	Consecutive	2013-2014	Refugees	30-70	51	5	-	-	NR

HS Juon, 2019(46)	CS	Convenience	2009-2015	Immigrants	37.3 ± 13	927	45	-	-	NR
M Ye, 2019ab(27)	CS	NR	2014	PWID	16-74	100	40	-	25	ELISA
M Chen, 2019(28)	CS	Convenient	2014	PWID	22-33	7	-	-	7	-
Myanmar IBBS, 2019(47)	CS	RDS	2017-2018	PWID	34.62 ± 9.25	6,061	3,394	-	-	Rapid test
KT Nyunt, 2019(19)	CS	NR	NR	General population	35.3	178	1	-	-	ICT
TS Win, 2020(48)	CS	NR	2016	PLHIV	35 (30-42)	15	150	-	-	ELISA
NTT Kyaw, 2022(49)	C	Consecutive	2005-2016	PLHIV	1-65	27,722	2,265	-	-	OraQuick
K Urban, 2023(50)	CS	Consecutive	2010-2017	Refugees	NR	18,059	511	-	-	NR
TM Swe, 2023abc(24)	C	Consecutive	2016-2017	PLHIV	15-80	21,777	1,417	1,143	640	OraQuick (HCV-Ab)<br>PCR (HCV RNA)

HCV-Ab, hepatitis C virus antibodies; *HCV RNA*; hepatitis C virus ribonucleic acid; *MOH*, Ministry of Health; *IBBS*, integrated biological and behavioral survey; *CS*, cross-sectional; *C*, cohort; *RC*, retrospective cohort; *NR*, not reported; *RDS*, respondent driven sampling; *HBV*, hepatitis B virus; *HCV*, hepatitis C virus; *HCC*, hepatocellular carcinoma; *PWID*, people who inject drugs; *PLHIV*, people living with human immunodeficiency virus; *ELISA*, enzyme-linked immunosorbent assay; *PCR*, polymerase chain reaction; *ICT*, immunochromatography; *IRMA*, immunoradiometric assay; *CLIA*, chemiluminescence immunoassay; *PA*, particle agglutination.

* Patients with liver diseases other than hepatocellular carcinoma; [†] Unpublished observations.

Study characteristics

Out of a total of 135 reports screened for eligibility, 35 reports comprising 51 studies were included in which 33 studies provided data on HCV seroprevalence in 685,403 individuals, 8 studies reported HCV RNA prevalence in 25,018 individuals, and 10 studies examined HCV genotypes in 1,857 individuals. The studies included in the analysis were conducted from 1991 to 2018 (Table 1). In HCV antibodies and RNA prevalence studies, the cross-sectional design was primarily used in 34 out of 41 studies (82.9%), while only one study(7) (2.4%) employed a probabilistic sampling method. In terms of sample size, 53.7% (22/41) of the studies included adequate sample sizes (>312). HCV antibodies and HCV RNA were determined through biological assays which were reported in 83.0% (34/41) of the studies (Additional file 1: Table S2).

Regarding the estimation of HCV seroprevalence, 28 out of 33 studies conducted cross-sectional studies, two studies utilized retrospective cohort designs in which the prevalence was estimated from laboratory records, and the remaining three studies employed cohort studies where HCV seroprevalence was determined as part of the overall study (Table 1). For the HCV RNA prevalence study, six out of eight studies performed cross-sectional studies, one study adopted a retrospective cohort, and one study employed a cohort study (Table 1). Out of the ten studies conducted for the HCV genotype study, five utilized cross-sectional study designs, three conducted a retrospective cohort approach, and two employed a cohort study methodology (Table 1). All included reports covered a study period ranging from 1991 to 2018, with the majority of studies conducted after 2010.

In terms of participant characteristics in the HCV seroprevalence estimate, nine studies focused on low-risk groups, including the general population, blood donors, and individuals who did not respond to HBV vaccines, encompassing a total of 60,648 participants. Eleven studies targeted high-risk groups, such as PWID, PLHIV, patients who received blood transfusions, and individuals undergoing hemodialysis, with a total of 57,101 participants. Seven studies focused on liver disease patients, specifically hepatocellular carcinoma (HCC), liver cirrhosis, and acute/chronic hepatitis, including 586 patients. Finally, six studies targeted refugees/immigrants, comprising a total of 21,218 individuals (Table 1).

In the HCV RNA prevalence study, two studies focused on low-risk groups, including blood donors and individuals who did not respond to HBV vaccines, with a total of 236 subjects. One study targeted a high-risk group of PLHIV, involving 21,777 individuals. Two studies focused on liver disease patients, including one study on HCC and one study on other liver diseases, encompassing 190 patients. Additionally, three studies specifically targeted refugees/immigrants, comprising a total of 2,815 individuals (Table 1).

In the study on HCV genotypes, three studies examined low-risk groups, which included the general population and blood donors, with a combined sample size of 363 subjects. Another three studies focused on high-risk groups such as PWID and PLHIV, involving 911 individuals. Two studies concentrated on patients with liver disease, encompassing a total of 552 patients. Furthermore, two studies specifically targeted refugees, with a total of 19 participants (Table 1).

Seroprevalence in the low-risk group

Low-risk populations were addressed in 9 studies and the total number of subjects screened was 606,498 of which 6,851 subjects were positive. Five studies focused on the general population, three on blood donors, and one on HBV vaccine non-responders. The seroprevalence ranged between 0 and 11.55% and the pooled seroprevalence of HCV was estimated at 2.18% (95%CI: 1.09-3.58). However, significant heterogeneity was found in this group (I^2=99.8%, p=0) (Figure 2)

Figure 2: Forest plot of the pooled HCV seroprevalence in Myanmar, using random effect

Seroprevalence in the high-risk group

Individuals in the high-risk group were investigated in eleven studies: five on PWID, three on PLHIV, two on patients who received blood transfusion, and one on hemodialysis patients. A total of 57,101 high-risk individuals were studied, and 7,930 were HCV antibodies-positive. Seroprevalence varied from 6.51% to 79.19%, and the pooled seroprevalence was 37.07% (95%CI: 22.85-52.49). Heterogeneity was also significant in this group (I^2=99.89%, p=0) (Figure 2).

Seroprevalence in the liver disease patients

Liver disease patients were investigated in seven studies: four on hepatocellular carcinoma patients and three on other liver disease patients. Out of a total of 586 patients, 174 were HCV antibodies positive. Seroprevalence varied from 20.00% to 48.28%, and the pooled seroprevalence was 33.84% (95%CI: 25.29-42.93). Heterogeneity was also significant in this group (I^2=65.13%, p=0.0086) (Figure 2).

Seroprevalence in the immigrants/refugees

Myanmar citizen immigrants/refugees who are living outside the country were investigated in six studies. Among a total of 21,218 individuals, 598 were HCV antibodies positive. Seroprevalence varied from 0 to 9.80% and the

pooled seroprevalence was 2.52% (95%CI: 1.52-3.73). Heterogeneity was also significant in this group (I^2=83.24%, p=<0.0001) (Figure 2).

Seroprevalence by study subjects

The highest HCV antibodies positive rate was found in PWID (58.06%, 95% CI: 56.94–59.18), individuals receiving blood transfusion (51.53%, 95% CI: 34.55–68.52), and liver disease patients (28.80%, 95% CI: 25.19-32.42). The seroprevalence of HCV among the general population was 2.82% (95% CI: 2.46–3.18, I^2 = 96.57%, $p < 0.0001$), and that among blood donors was 0.68% (95% CI: 0.66-0.70, I^2=99.92%, p=<0.00001) (Table 2).

Table 2: HCV seroprevalence in sub-groups

Categories	Number of studies	Total population	Pooled seroprevalence	95% CI	I^2 index (%)	Cochran Q test p-value
Study subjects						
General population	5	7,989	2.82	2.46-3.18	96.57	<0.00001
Blood donors	3	598,486	0.68	0.66-0.70	99.92	<0.00001
HBV vaccine non-responders	1	23	0.00	0.00-0.00	NA	NA
PWID	5	7,308	58.06	56.94-59.18	97.49	<0.00001
PLHIV	3	49,649	7.36	7.13-7.59	96.12	<0.00001
Patients who received blood transfusion	2	33	51.53	34.55-68.52	NA	NA
Haemodialysis patients	1	111	15.32	8.62-22.02	NA	NA
Liver disease patients*	7	586	28.80	25.19-32.42	63.87	0.01
Refugees/immigrants	6	21,218	2.72	2.50-2.94	79.70	0.0002
Data collection period †						
2000-2005	1	154,161	2.60	2.52-2.70	NA	NA
2006-2010	1	65,237	0.95	0.90-1.03	NA	NA

2011-2015	2	384,635	0.51	0.48-0.53	99.99	<0.00001

HBV, hepatitis B virus; *PWID*, people who inject drugs; *PLHIV*, people living with human immunodeficiency virus; *NA*, not applicable.

* Patients with liver diseases including hepatocellular carcinoma; [†] Analyses performed considering only low-risk groups (blood donors and general population).

Seroprevalence by the study period of data collection

Only low-risk groups were considered for HCV seroprevalence estimate by the study period to see the pure trend of its prevalence. One unpublished study was conducted between 2000-2005, resulting in a seroprevalence of 2.60% (95%CI: 2.52-2.70). The seroprevalence decreased to 0.95% (95%CI: 0.90-1.03, one study(20)) during 2006-2010 and to 0.51% (95%CI: 0.48-0.53, I^2=99.99%, p=<0.00001) during 2011-2015 (two studies including one unpublished study(7)) (Table 2). This decreasing trend was significant (p for difference=0.03).

HCV RNA prevalence

Eight studies(17, 21-25) measured HCV RNA, and the positive rate ranged from 0.37% in refugees to 25.45% in liver disease patients. The pooled prevalence of HCV RNA was estimated at 1.40% (95%CI: 0.08-3.71, I^2=0%, p=0.3390) in low-risk group, 5.25% (95%CI: 4.96-5.55) in high-risk group, 24.96% (95%CI: 18.91-31.51, I^2=0%, p=0.0115) in patients with liver diseases and 0.84% (95%CI: 0.19-1.84, I^2=73.65%, p=0.0225) in refugees/immigrants (Figure 3).

Figure 3: Forest plot of the pooled HCV RNA prevalence in Myanmar, using random effect

HCV genotypes

The genotyping of the HCV was performed in 10 studies(13, 16, 18, 21, 23-28). Overall, HCV genotype 3 was most predominant (48%) followed by genotypes 1 (28%), 6 (23%), 2 (1%), and 4 (0.27%). By sub-group analysis, genotype 3 is predominant in all sub-groups of low-risk, high-risk, liver disease patients and refugees/immigrants (43%, 48%, 52%, and 53% respectively). The HCV genotype distribution pattern was same between low-risk groups and refugees/immigrants: genotype 3 (43% vs. 53%) followed by 6 (36% vs. 37%) and 1 (21% vs. 11%). The HCV genotype distribution was the same in high-risk groups and liver disease patients: genotype 3 (48% vs 52%) followed by 1 (31% vs. 28%) and 6 (21% vs. 18%). HCV genotype 2 was exclusively found in low-risk and liver disease patients (1% and 2% respectively) and only one case of genotype 4 was reported in a high-risk group (PLHIV) (Figure 4). The analysis of HCV genotype distribution by region showed that genotype 6 is predominant in the northern part of Myanmar whilst genotype 3 is predominant in the west and southern parts of Myanmar (Figure 5).

Figure 4: Distribution of HCV genotypes in Myanmar

Figure 5: Distribution of HCV genotypes by study place in Myanmar (N=1,294)

Publication bias

Using the graphical assessment of the funnel plot, the publication bias was found in HCV seroprevalence studies, but there was no publication bias in HCV RNA prevalence studies. In the Egger tests for funnel plot asymmetry, the p-values were 0.0001 and 0.8392 respectively (Figure 6a and 6b).

Figure 6: Funnel plot of HCV prevalence a) HCV antibodies b) HCV RNA

Discussion

This systematic review revealed Myanmar has the intermediate endemicity of HCV seroprevalence among low-risk groups including the general population. Analyzing HCV seroprevalence by sub-groups provided a broader understanding of the disease burden in each group in Myanmar. The disease spectrum differs by the characteristic of each group, and it is encountered to consider adopting effective countermeasures against HCV in Myanmar.

This meta-analysis revealed publication bias in HCV seroprevalence studies but not in HCV RNA prevalence studies. The presence of publication bias indicates a likelihood of overestimating seroprevalence rates due to the selective publication of studies with higher rates. Therefore, the pooled prevalence was not determined as a whole, and the participants were categorized into four groups: low-risk groups, high-risk groups, liver disease patients, and immigrants/refugees computed the pooled prevalence in each group using a random effect. This meta-analysis included the Burmese immigrants or refugees residing in other countries and it is believed that this group of the population reflects the disease burden of HCV among the general population in Myanmar.

HCV seroprevalence in low-risk groups and immigrants/refugees were 2.18% and 2.52% respectively. A significantly high level of heterogeneity was noticed in both groups (I^2: 99.8% vs. 83.24%). Considering the prevalence of 2.18% in the general population, 1.18 million individuals were exposed to HCV infection across the country as of 2023 Myanmar's population data(29). In Myanmar, a nationwide study on the prevalence of HBV and HCV was conducted in 18 study sites in 2015 and reported that the prevalence of HBV and HCV was 6.5% and 2.7% respectively(7). The pooled HCV seroprevalence among low-risk groups and immigrants/refugees in this meta-analysis is consistent with a 2015 nationwide study. As the studies among the abovementioned groups of the population were conducted from 2000 through 2017, it is supposed not to reflect the current epidemiological situation of HCV among the general population in Myanmar.

The pooled HCV seroprevalence of 37.07% was found in high-risk groups which comprised of frequent blood

recipients (Thalassemia etc.), hemodialysis patients, PWID, and PLHIV, all of whom were highly prone to HCV infection. A significant high level of heterogeneity was observed in this group with reported I^2 at 99.8%. The reported studies were conducted from 1996 through 2018. The high HCV seroprevalence rates among PWID, ranging from 40% to 79%, highlight the critical importance of implementing targeted interventions although harm reduction strategies that have been introduced since 2005-2006 in Myanmar. Moreover, the HIV/HCV coinfection rate ranged from 5% to 22.8% and the risk was high in the PWID population of Myanmar (Odds Ratio: 20.1, 95% CI: 13.7 to 29.5)(30, 31) which highlights the interplay between these two infections and the need for integrated prevention and treatment approaches in PWID.

The pooled HCV seroprevalence among liver disease patients was calculated to be 33.84% with a reported medium heterogeneity of 65.13%. All the studies on liver disease patients were conducted between 1991 and 1998 and there was no report or studies after 2002. Most of the studies used convenient sampling methods for participant recruitment and the under or overestimation of HCV seroprevalence among this group cannot be ruled out. After two decades, the attribution of HBV and HCV etiology to liver disease including HCC might be changed and it needs to be evaluated again.

The decreasing trend of HCV seroprevalence among the low-risk group was found by a period of data collection. It can be explained by the installation of blood donor screening in 2000, the promotion of infection control including disposable syringe usage in health care facilities in the early 2000 and raising the awareness of viral hepatitis in 2011 with local and international non-governmental organizations' involvement in viral hepatitis screening and health education.

HCV RNA positive rate was supposed to be low in low-risk and immigrants/refugee groups (1.4% vs 0.84%) assuming that approximately 50% of them were naturally resolved and the number of viremic cases was estimated at 0.7 million individuals in Myanmar. The high HCV RNA-positive rate was found in liver disease patients (24.96%) but its attribution to hepatocellular carcinoma is unknown.

There were no incidence studies on HCV infection in Myanmar and only a few of the included prevalence studies were conducted mostly in an urban area with representative samples of the target population and there were no studies or reports on HCV disease burden after the 2015 nationwide study. Furthermore, the coronavirus disease 2019 (COVID-19) pandemic together with the unstable political situation in 2021 delayed or interrupted the healthcare system in Myanmar which is threatening the infectious diseases control, countermeasures, and the road to elimination of viral hepatitis in Myanmar. A well-structured basic epidemiological study to examine the disease

burden of HCV in Myanmar is recommended.

In Myanmar, the predominant HCV genotype was 3 (48%) followed by 1 (28%) and 6 (23%) overall. This information is significant in guiding HCV treatment strategies, as genotypes can impact disease progress, treatment response, and duration. For instance, genotype 3 is associated with a higher risk of developing severe liver injuries, such as cirrhosis and hepatocellular carcinoma(32). The obvious geographical difference in genotype distribution was found across the country. In the northern region of Myanmar, which shares a border with China's Yunnan province, a high prevalence of HCV genotype 6 was observed in the PWID population. Likewise, PWID in Yunnan province, HCV genotype 6 was found to be the predominant genotype. This finding suggests that there may be transmission of HCV among PWID populations occurring through the border regions between Myanmar and Yunnan province(33). However, HCV genotype 3 was predominant in the southern part of Myanmar, and the southern east of Myanmar is bordered by Thailand where genotype 3 is reportedly predominant(34). HCV genotype 3 was also predominant in the western part of Myanmar, which shares borders with India and Bangladesh. These neighboring countries also have a high prevalence of genotype 3(35, 36). Understanding the genotype distribution and its geographical difference revealed the potential route of transmission from which effective prevention and control strategies should be developed. The genotypic diversity observed in Myanmar serves as a valuable contribution to the overall understanding of HCV genotypes in the Southeast Asia region.

This study has several limitations. The significant heterogeneity among the included studies may introduce variations in the estimated prevalence rates. This heterogeneity can be attributed to differences in sampling methods, individual characteristics of the study population, involvement of small sample sizes in some studies, and sampling biases in each recruited study so that the over or underestimation of the prevalence cannot be excluded. Most of the included studies did not provide the study setting clearly so that the urban and rural areas cannot be categorized, but most studies were assumed to be conducted in urban settings so the study in rural settings is limited in Myanmar and the actual disease burden among rural population is unknown. Moreover, the study on HCV among not only key populations such as men who have sex with men (MSM), female sex workers (FSW), and people in prisons but also the health care professionals (HCP) and pregnant women are still unknown.

In conclusion, this systematic review and meta-analysis indicated that Myanmar has intermediate endemicity of HCV seroprevalence in the low-risk group with a decreasing trend over the past 20 years due to measures such as blood donor screening, infection control practices, and increased awareness through education programs. However, the findings highlight the need for further epidemiological studies to understand the actual disease burden and

implement effective countermeasures, including screening, linkage to care, and accessibility to treatment, to achieve the WHO's goal of HCV elimination by 2030.

List of abbreviations

HCV: Hepatitis C virus; DAA: Direct-acting antivirus; RNA: Ribonucleic acid; WHO: World Health Organization; CI: Confidence Interval; MMA: Myanmar Medical Association; MLF: Myanmar Liver Foundation; MSF: Medecins Sans Frontieres; NHCP: National Hepatitis Control Program; SVR: Sustained virological response; PLHIV: People living with human immunodeficiency virus; PWID: People who inject drugs; HBV: Hepatitis B Virus HCC: Hepatocellular carcinoma; HIV: Human immunodeficiency virus; MSM: Men having sex with men; FSW: Female sex workers; HCP: Health care professional.

Declarations

Ethical approval and consent to participate

Not applicable.

Consent for publication

Not applicable.

Availability of data and materials

The dataset used and analysed in this study is available from the corresponding author upon reasonable request.

Competing interests

The authors declare no conflict of interest.

Funding

This work was supported by Japan’s Ministry of Health, Labour and Welfare (MHLW) Policy Research for Hepatitis Measures Program Grant Number JPMH22HC1001.

Authors’ contributions

ZP, KK, and JT conceptualized the study. ZP, KK, and SO contributed to developing the study design and data acquisition. ZP, KK, SO, TA, and JT analysed and interpreted the data. ZP, KK, and SO drafted the manuscript. AS, UM, GA, CC, TA, and JT contributed to the intellectual content of the manuscript. All authors read and approved the final manuscript.

Acknowledgment

We would send heartfelt gratitude to all the authors of the original papers which were used in this systematic review and meta-analysis. We would like to thank the personnel of the Department of Epidemiology, Infectious Disease Control, and Prevention, Graduate School of Biomedical and Health Sciences, Hiroshima University, for their valuable support.

References

1. World Health Organization (WHO). HCV Fact Sheet (24 June 2022). Available at: https://www.who.int/news-room/fact-sheets/detail/hepatitis-c. Accessed June 28, 2023.

2. World Health Organization (WHO). Global Hepatitis Report 2017. Available at: https://www.who.int/publications/i/item/9789241565455. Accessed 3 July 2023.

3. Polaris Public Access, Regional Dashboard. Available at https://cdafound.org/polaris-regions-compare/. Accessed June 28 2023.

4. National Hepatitis Control Program, Ministry of Health and Sports, Myanmar. Myanmar National Strategic Plan on Viral Hepatitis 2016-2020. 2017. Available at: https://www.aidsdatahub.org/resource/myanmar-national-strategic-plan-viral-hepatitis-2016-2020. Accessed 3 July 2023.

5. Ministry of Health and Sports, Myanmar. Simplified Treatment Guidelines for Hepatitis C Infection: Myanmar (2017). Available at: https://www.aidsdatahub.org/resource/simplified-treatment-guidelines-hepatitis-c-infection-myanmar.

6. Draper BL, Pedrana A, Howell J, Yee WL, Htay H, Aung KS, et al. Decentralized, Community-Based Hepatitis C Point-of-Care Testing and Direct-Acting Antiviral Treatment for People Who Inject Drugs and the General Population in Myanmar: Protocol for a Feasibility Study. JMIR Res Protoc. 2020;9(7):e16863.

7. Lwin AA, Aye KS, Htun MM, Kyaw YY, Zaw KK, Aung TT, Kyaw MP, Kyi KP, Thant KZ. Sero-prevalence of Hepatitis B and C Viral Infections in Myanmar: National and Regional Survey in 2015. Myanmar Health Sciences Research Journal, Vol. 29, No. 3, 2017.

8. Thu A, Thaw A, Oo KM, Lwin AA, Lwin O, Myint AZ, Oo SS, Aung PP, Phyu AH. Prevalence and associated risks of hepatitis B and C infections among Injecting Drug Users (IDU) attending the registered Drug Treatment Centers in Yangon, Mandalay, Myitkyina, Bamaw, and Moekaung in 2007. Myanmar Health Sciences Research Journal. 2008; 20(3): 159-164.

9. Page MJ, McKenzie JE, Bossuyt PM, Boutron I, Hoffmann TC, Mulrow CD, et al. The PRISMA 2020 statement: an updated guideline for reporting systematic reviews. Bmj. 2021;372:n71.

10. Munn Z, Moola S, Lisy K, Riitano D, Tufanaru C. Methodological guidance for systematic reviews of observational epidemiological studies reporting prevalence and cumulative incidence data. Int J Evid Based Healthc. 2015;13(3):147-53.

11. Higgins JP, Thompson SG, Deeks JJ, Altman DG. Measuring inconsistency in meta-analyses. Bmj. 2003;327(7414):557-60.

12. Hunter JP, Saratzis A, Sutton AJ, Boucher RH, Sayers RD, Bown MJ. In meta-analyses of proportion studies, funnel plots were found to be an inaccurate method of assessing publication bias. J Clin Epidemiol. 2014;67(8):897-903.

13. Mellor J, Walsh EA, Prescott LE, Jarvis LM, Davidson F, Yap PL, et al. Survey of type 6 group variants of hepatitis C virus in Southeast Asia by using a core-based genotyping assay. J Clin Microbiol. 1996;34(2):417-23.

14. Kyi KP, Aye M, Oo KM, Htun MM, Oo SS, Lwin KO, Win KM. Prevalence of Hepatitis C in Healthy Population and Patients with Liver Ailments in Myanmar. Regional Health Forum, Volume 6, Number 1, 2002.

15. Taketa K, Okada S, Win N, Hlaing NK, Wind KM. Evaluation of tumor markers for the detection of hepatocellular carcinoma in Yangon General Hospital, Myanmar. Acta Med Okayama. 2002;56(6):317-20.

16. Lwin AA, Shinji T, Khin M, Win N, Obika M, Okada S, et al. Hepatitis C virus genotype distribution in Myanmar: Predominance of genotype 6 and existence of new genotype 6 subtype. HEPATOLOGY RESEARCH. 2007;37(5):337-45.

17. Htun MM, Kyi KP, Oo KM, Khin M, Tun WM, Oo SS, Lwin KO, Abe K. Presence of hepatitis C virus RNA by nested polymerase chain reaction (RT-PCR) in plasma-derived hepatitis B vaccine non-responders. Myanmar Health Sciences Research Journal. 2010; 22(2): 101-104.

18. Naing C, Sitt T, Aung AT, Aung K. Sustained Virologic Response to a Dual Peginterferon alfa-2a and Ribavirin in Treating Chronic hepatitis C Infection: A Retrospective Cohort Study. Medicine (Baltimore). 2015;94(30):e1234.

19. Nyunt KT, Htoo KPP, Hnin MT, Kyaw PM, Mon EK, Thu ISL, Aye MM. Hepatitis B and Hepatitis C infection among teaching staff and non-teaching from University of Medical Technology, Mandalay. Myanmar Medical Journal, 2019; 61(4):35-43.

20. Myo K, San San O, Oo KM, Shimono K, Koide N, Okada S. Prevalence and Factors Associated with Hepatitis C Virus Infection among Myanmar Blood Donors. ACTA MEDICA OKAYAMA. 2010;64(5):317-21.

21. Nakai K, Win KM, Oo SS, Arakawa Y, Abe K. Molecular characteristic-based epidemiology of hepatitis B, C, and E viruses and GB virus C/hepatitis G virus in Myanmar. JOURNAL OF CLINICAL MICROBIOLOGY. 2001;39(4):1536-9.

22. Chaves NJ, Gibney KB, Leder K, O'Brien DP, Marshall C, Biggs BA. Screening practices for infectious diseases among Burmese refugees in Australia. Emerg Infect Dis. 2009;15(11):1769-72.

23. Mixson-Hayden T, Lee D, Ganova-Raeva L, Drobeniuc J, Stauffer WM, Teshale E, et al. Hepatitis B virus and hepatitis C virus infections in United States-bound refugees from Asia and Africa. Am J Trop Med Hyg. 2014;90(6):1014-20.

24. Swe TM, Johnson DC, Mar HT, Thit P, Homan T, Chu CM, et al. Epidemiological characteristics and real-world treatment outcomes of hepatitis C among HIV/HCV co-infected patients in Myanmar: A prospective cohort study. Health Sci Rep. 2023;6(2):e1119.

25. Akkarathamrongsin S, Praianantathavorn K, Hacharoen N, Theamboonlers A, Tangkijvanich P, Poovorawan Y. Seroprevalence and Genotype of Hepatitis C Virus among Immigrant Workers from Cambodia and Myanmar in Thailand. INTERVIROLOGY. 2011;54(1):10-6.

26. Shinji T, Kyaw YY, Gokan K, Tanaka Y, Ochi K, Kusano N, et al. Analysis of HCV genotypes from blood donors shows three new HCV type 6 subgroups exist in Myanmar. ACTA MEDICA OKAYAMA. 2004;58(3):135-42.

27. Ye M, Chen X, Wang Y, Duo L, Zhang C, Zheng YT. Identification of a New HCV Subtype 6xg Among Injection Drug Users in Kachin, Myanmar. Front Microbiol. 2019;10:814.

28. Chen M, Ma Y, Chen H, Dai J, Luo H, Jia M, et al. Complete genome sequencing and evolutionary analysis of HCV subtype 6xg from IDUs in Yunnan, China. PLoS One. 2019;14(5):e0217010.

29. Myanmar Population (2023), Worldometers. Available at: https://www.worldometers.info/world-population/myanmar-population/. Accessed 28 June 2023.

30. Ousley J, Nesbitt R, Kyaw NTT, Bermudez E, Soe KP, Anicete R, et al. Increased hepatitis C virus co-infection and injection drug use in HIV-infected fishermen in Myanmar. BMC INFECTIOUS DISEASES. 2018;18.

31. Zaw SK, Tun ST, Thida A, Aung TK, Maung W, Shwe M, et al. Prevalence of hepatitis C and B virus among patients infected with HIV: a cross-sectional analysis of a large HIV care program in Myanmar. Trop Doct. 2013;43(3):113-5.

32. Gondeau C, Pageaux GP, Larrey D. Hepatitis C virus infection: Are there still specific problems with genotype 3? World J Gastroenterol. 2015;21(42):12101-13.

33. Zhang Z, Yao Y, Wu W, Feng R, Wu Z, Cun W, et al. Hepatitis C virus genotype diversity among intravenous drug users in Yunnan Province, Southwestern China. PLoS One. 2013;8(12):e82598.

34. Wasitthankasem R, Vongpunsawad S, Siripon N, Suya C, Chulothok P, Chaiear K, et al. Genotypic Distribution of Hepatitis C Virus in Thailand and Southeast Asia. PLOS ONE. 2015;10(5).

35. Das BR, Kundu B, Khandapkar R, Sahni S. Geographical distribution of hepatitis C virus genotypes in India. Indian J Pathol Microbiol. 2002;45(3):323-8.

36. Islam MS, Miah MR, Roy PK, Rahman O, Siddique AB, Chowdhury J, et al. Genotypes of hepatitis C virus infection in Bangladeshi population. Mymensingh Med J. 2015;24(1):143-51.

37. Kyi KP, Win KM, Aye M, Htwe YY, Oo KM, Aung T, Oo SS. Prevalence of hepatitis B and C infections in hepatocellular carcinoma cases in Myanmar. Myanmar Health Sciences Research Journal. 1998; 10(1): 1-5.

38. Okada S, Taketa K, Ishikawa T, Koji T, Swe T, Win N, et al. High prevalence of hepatitis C in patients with thalassemia and patients with liver diseases in Myanmar (Burma). ACTA MEDICA OKAYAMA. 2000;54(3):137-8.

39. Win N, Okada S, Win KM, Taketa K, Akiyama T, Aye KS, et al. Hemoglobinopathies, Iron Overload and Chronic Viral Hepatitis in Patients with Hepatocellular Carcinoma in Myanmar. Asian Pac J Cancer Prev. 2000;1(2):127-30.

40. Denburg A, Rashid M, Brophy J, Curtis T, Malloy P, Audley J, et al. Initial health screening results for Karen refugees: a retrospective review. Can Commun Dis Rep. 2007;33(13):16-22.

41. Zhou YH, Liu FL, Yao ZH, Duo L, Li H, Sun Y, et al. Comparison of HIV-, HBV-, HCV- and Co-Infection Prevalence between Chinese and Burmese Intravenous Drug Users of the China-Myanmar Border Region. PLOS ONE. 2011;6(1).

42. Paxton GA, Sangster KJ, Maxwell EL, McBride CR, Drewe RH. Post-arrival health screening in Karen refugees in Australia. PLoS One. 2012;7(5):e38194.

43. Lwin AA, Htwe KM, Mon NCN, Maw KT, Hlaing MM, Thwin KT, Aye KS. Hepatitis B and C Viral Infections among Hemodialysis Patients Attending 500 Bedded Yangon Specialty Hospital, Myanmar. Myanmar Health Sciences Research Journal, Vol. 30, No. 3, 2018.

44. Aye NS, Oo MM, Harries AD, Mon MM, Hone S, Oo HN, et al. HIV, HBV, and HCV in people who inject drugs and are placed on methadone maintenance therapy, Yangon, Myanmar. PUBLIC HEALTH ACTION. 2018;8(4):202-10.

45. Ngo CC, Maidment C, Atkins L, Eagar S, Smith MM. Blood screen findings in a 2-year cohort of newly arrived refugees to Sydney, Australia. Public Health Res Pract. 2018;28(1).

46. Juon HS, Ha E, Kim F, Trang A, Pan J, Blanchard J. Prevalence of Viral Hepatitis in Foreign-Born Populations in the Baltimore-Washington Metropolitan Area, 2009-2015. JOURNAL OF COMMUNITY HEALTH. 2019;44(2):203-7.

47. National AIDS Programme (NAP), Myanmar Ministry of Health and Sports, Myanmar. Myanmar Integrated Biological and Behavioural Surveillance Survey and Population Size Estimates among People Who Inject Drugs (PWID) 2017-2018. Available at https://www.aidsdatahub.org/resource/myanmar-ibbs-population-size-estimates-pwid-2017-2018.

48. Win TS, Maw SY, Kyaw YY, Maw YY. Serodetection of Hepatitis C Virus Infection among HIV Patients in Mingalardon Specialist Hospital. Myanmar Health Sciences Research Journal, 2020; 32(1):19-24.

49. Kyaw NTT, Satyanarayana S, Harries AD, Kumar AMV, Kyaw KWY, Phyo KH, et al. Increased All-cause Mortality in People With HIV and Comorbidities: Hepatitis B and C Virus Seropositivity and Hyperglycemia in Myanmar, 2005-2017. Open Forum Infect Dis. 2023;10(1):ofac672.

50. Urban K, Payton C, Mamo B, Volkman H, Giorgio K, Kennedy L, et al. Hepatitis C Screening and Antibody Prevalence Among Newly Arrived Refugees to the United States, 2010-2017. J Immigr Minor Health. 2023:1-8.

Figure Legends

Figure 1: PRISMA showing a selection of articles for meta-analysis of HCV in Myanmar

This figure shows the selection process of the articles for the estimation of HCV prevalence in Myanmar. Each arrow indicates the step-by-step process for recruiting the articles and the detailed reason for the exclusion of articles were described with their respective exact number. This figure was built following the guideline for 2020 Preferred Reporting Items for Systematic Reviews and Meta-Analysis (PRISMA).

Figure 2: Forest plot of the pooled HCV seroprevalence in Myanmar, using random effect

This figure shows the funnel plot of 33 studies and the pooled HCV seroprevalence in sub-groups: low-risk group, high-risk group, liver disease patients, and refugees/immigrants. The diamond block represents for pooled prevalence and the rectangular block represents prevalence for each study.

Figure 3: Forest plot of the pooled HCV RNA prevalence in Myanmar, using random effect

This figure shows the funnel plot of 7 studies and the pooled HCV RNA prevalence in sub-groups: low-risk group, liver disease patients, and refugees/immigrants. The diamond block represents for pooled prevalence and the rectangular block represents prevalence for each study.

Figure 4: Distribution of HCV genotypes in Myanmar

This figure shows the HCV genotype distribution overall and by group of population in Myanmar. Each color represents each genotype: light blue for Genotype 1, orange for Genotype 2, dark blue for Genotype 3, and light green for Genotype 6.

Figure 5: HCV genotypes distribution by study place in Myanmar (N=1,294)

This figure shows the analysis of HCV genotype distribution by region in Myanmar. Each pie chart represents each study place. Each color inside the pie chart represents each genotype: light blue for Genotype 1, orange for Genotype 2, dark blue for Genotype 3, and light green for Genotype 6.

Figure 6: Funnel plot of HCV prevalence a) HCV antibodies and b) HCV RNA

Figures 6a and 6b show the forest plot for the assessment of publication bias among the recruited studies for estimating pooled HCV antibodies and HCV RNA prevalence. The dotted line represents the resultant pooled prevalence, and each black circle represents the prevalence of each study. The prevalence was plotted against the sample size.

Supporting information captions

S1 Appendix. PRISMA checklist

S1 Table. Database search

S2 Table. Quality appraisal of included studies using the Joanna Briggs Institute checklist for prevalence studies

Figure 1 Click here to access/download;Figure;Figure 1.pdf

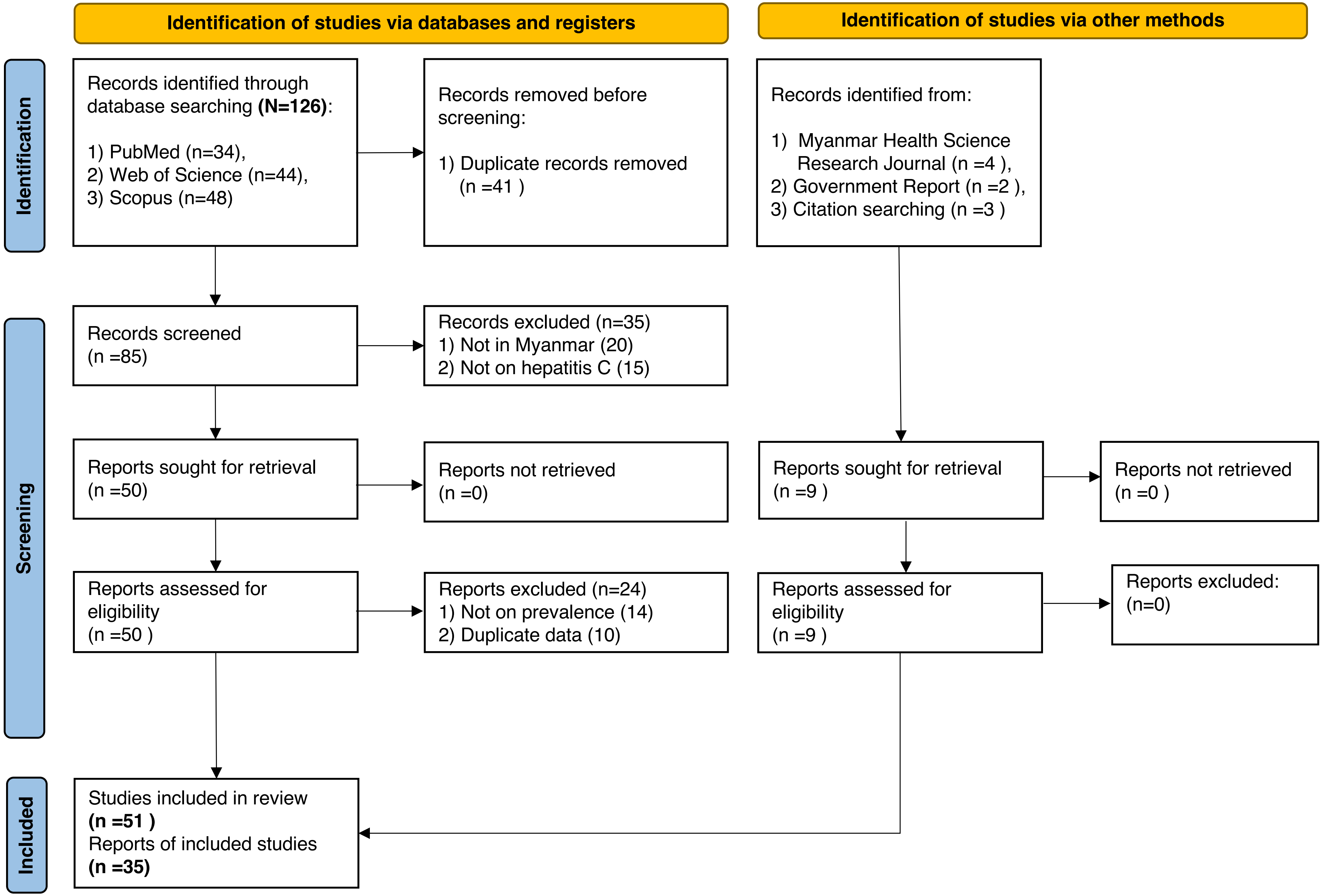

Figure 1: PRISMA showing selection of articles for meta-analysis of HCV in Myanmar

Click here to access/download;Figure;Figure 2.pdf

Author, Publication year	Subjects	Period	Cases	Total	HCV-Ab Prevalence	[95% CI]	Weight
**Low risk group**							
M Khin. et al 2003	Blood donors	2000-2004	4008	154161	2.60	[2.52; 2.68]	11.9%
M Khin. et al 2010	Blood donors	2005-2007	621	65237	0.95	[0.88; 1.03]	11.9%
Ministry of Health, 2015	Blood donors	2015	1895	379088	0.50	[0.48; 0.52]	11.9%
AA Lwin. et al 2017	General population	2015	147	5547	2.65	[2.24; 3.11]	11.8%
M Khin. et al 2000	General population	NR	16	569	2.81	[1.62; 4.53]	11.5%
KP Kyi et al 2002a	General population	NR	9	362	2.49	[1.14; 4.67]	11.4%
AA Lwin. et al 2007a	General population	NR	154	1333	11.55	[9.89; 13.39]	11.7%
MM Htun. et al 2010a	Hepatitis B vaccine non-responders	NR	0	23	0.00	[0.00; 14.82]	7.1%
KT Nyunt. et al 2019	General population	NR	1	178	0.56	[0.01; 3.09]	10.9%
**Pooled HCV-Ab Prevalence**				**606498**	**2.18**	**[1.09; 3.58]**	**100.0%**
Heterogeneity: I^2 = 99.8%, p = 0							
**High risk group**							
O Shigeru. et al 2000a	Thalassemia	1996-1998	7	15	46.67	[21.27; 73.41]	5.2%
O Shigeru. et al 2000b	Blood receipts	1996-1998	10	18	55.56	[30.76; 78.47]	5.6%
NTT Kyaw. et al 2022	HIV	2005-2016	2265	27722	8.17	[7.85; 8.50]	10.5%
A Thu. et al 2008	PWID	2007-2008	236	298	79.19	[74.14; 83.66]	10.0%
YH Zhou. et al 2011	PWID	2005-2007	153	318	48.11	[42.50; 53.76]	10.0%
M Ye. et al 2019a	PWID	2014	40	100	40.00	[30.33; 50.28]	9.0%
NS Aye. et al 2018	PWID	2015-2017	376	531	70.81	[66.74; 74.65]	10.2%
AA Lwin. et al 2018	Hemodialysis Patients	2016	17	111	15.32	[9.18; 23.39]	9.2%
TS Win. et al 2020	HIV	2016	15	150	10.00	[5.71; 15.96]	9.5%
TM Swe. et al 2023a	HIV	2016-2017	1417	21777	6.51	[6.18; 6.84]	10.5%
IBBS, 2019	PWID	2017-2018	3394	6061	56.00	[54.74; 57.25]	10.5%
**Pooled HCV-Ab Prevalence**				**57101**	**37.07**	**[22.85; 52.49]**	**100.0%**
Heterogeneity: I^2 = 99.89%, p = 0							
**Liver disease patients**							
KP Kyi. et al 1998	HCC	1991	14	40	35.00	[20.63; 51.68]	14.8%
N Win. et al 2000	HCC	1996	7	16	43.75	[19.75; 70.12]	10.5%
O Shigeru. et al 2000c	Liver diseases	1996-1998	32	74	43.24	[31.77; 55.28]	16.9%
O Shigeru. et al 2000d	HCC	1996-1998	8	28	28.57	[13.22; 48.67]	13.2%
T Kazuhisa.et al 2002b	Liver diseases	NR	14	29	48.28	[29.45; 67.47]	13.3%
T Kazuhisa.et al 2002a	HCC	NR	4	20	20.00	[5.73; 43.66]	11.6%
KP Kyi et al 2002b	Liver diseases	NR	95	379	25.07	[20.78; 29.75]	19.7%
**Pooled HCV-Ab Prevalence**				**586**	**33.84**	**[25.29; 42.93]**	**100.0%**
Heterogeneity: I^2 = 65.13%, p = 0.0086							
**Refugees/Immigrants**							
A Denburg. et al 2007	Refugees	2006	0	68	0.00	[0.00; 5.28]	14.7%
GA Paxton. et al 2012	Refugees	2006-2007	10	519	1.93	[0.93; 3.51]	17.6%
A Srunthron. et al 2010a	Immigrants	2007-2009	27	1594	1.69	[1.12; 2.45]	17.9%
HS Juon. et al 2019	Immigrants	2009-2015	45	927	4.85	[3.56; 6.44]	17.8%
K Urban. et al 2023	Refugees	2010-2017	511	18059	2.83	[2.59; 3.08]	18.1%
CC Ngo. et al 2018	Refugees	2013-2014	5	51	9.80	[3.26; 21.41]	13.8%
**Pooled HCV-Ab Prevalence**				**21218**	**2.52**	**[1.52; 3.73]**	**100.0%**
Heterogeneity: I^2 = 83.24%, p < 0.0001							
Test for subgroup differences: χ^2_3 = 126.19, df = 3 (p < 0.0001)							

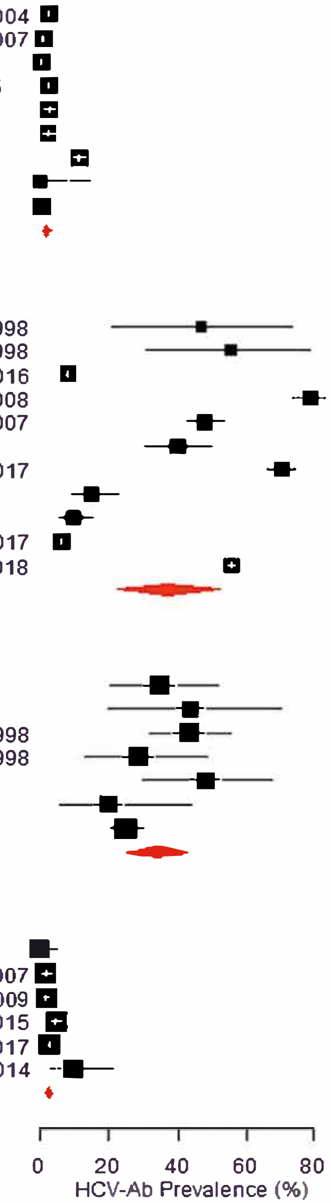

Figure 2: Forest plot of the pooled HCV seroprevalence in Myanmar, using random effect

Figure 3
Click here to access/download;Figure;Figure 3.pdf

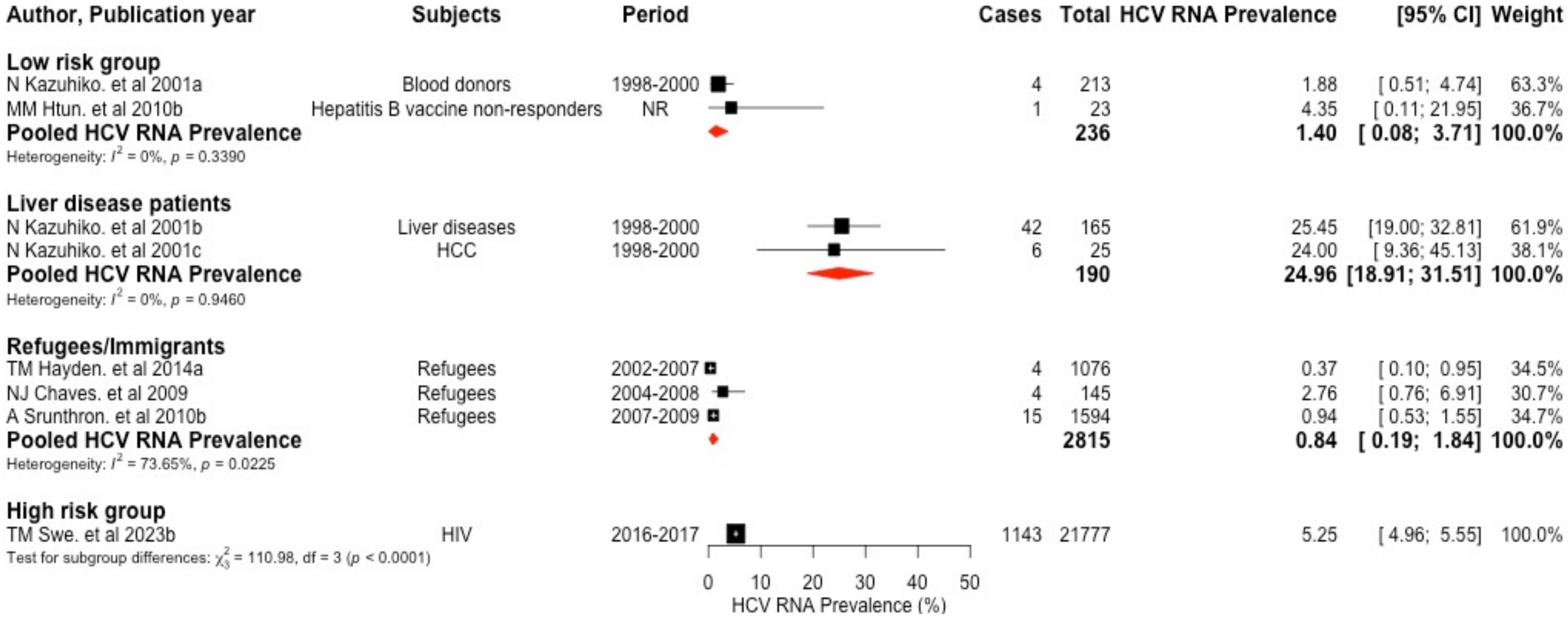

Figure 3: Forest plot of the pooled HCV RNA prevalence in Myanmar, using random effect

Click here to access/download;Figure;Figure 4.pdf

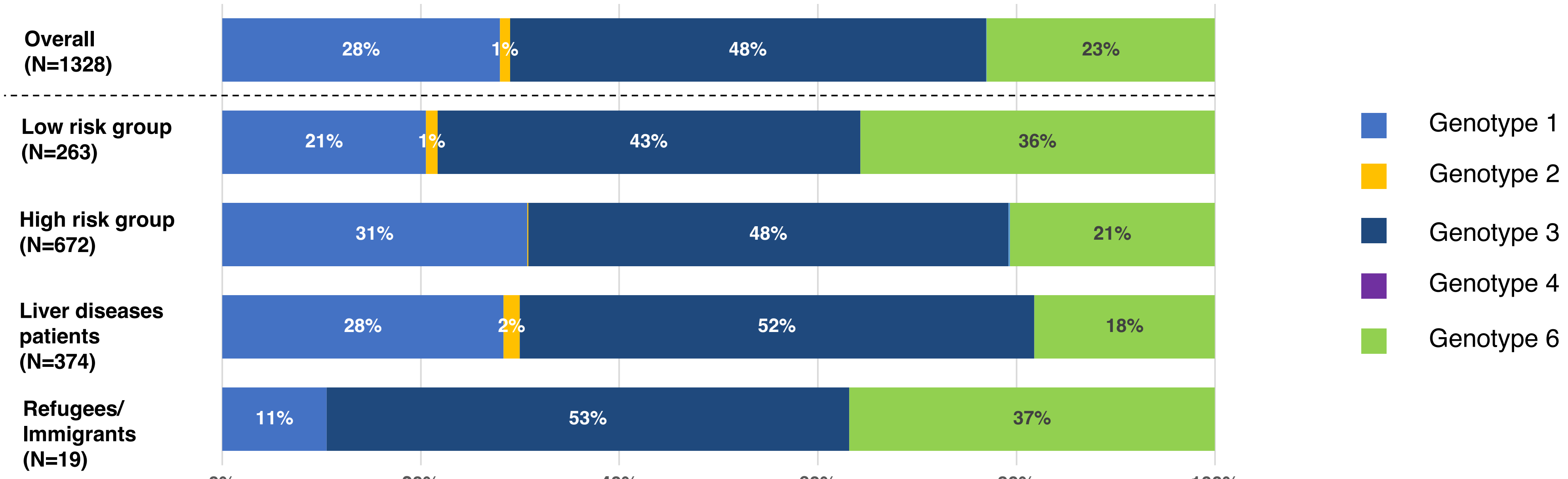

Figure 4: Distribution of HCV genotypes in Myanmar

Figure 5

Click here to access/download;Figure;Figure 5.pdf

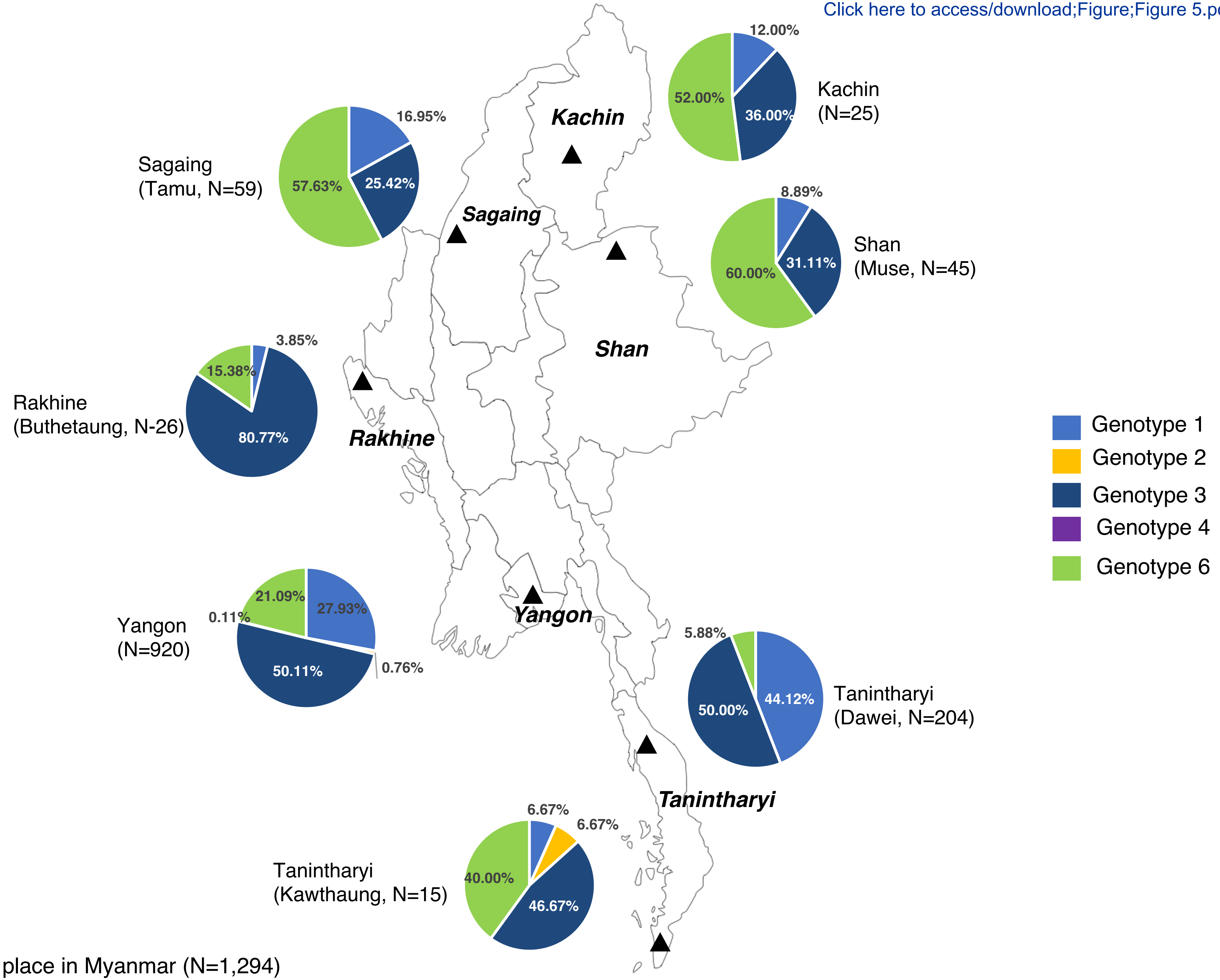

Figure 5: Distribution of HCV genotypes by study place in Myanmar (N=1,294)

Click here to access/download;Figure;Figure 6.pdf

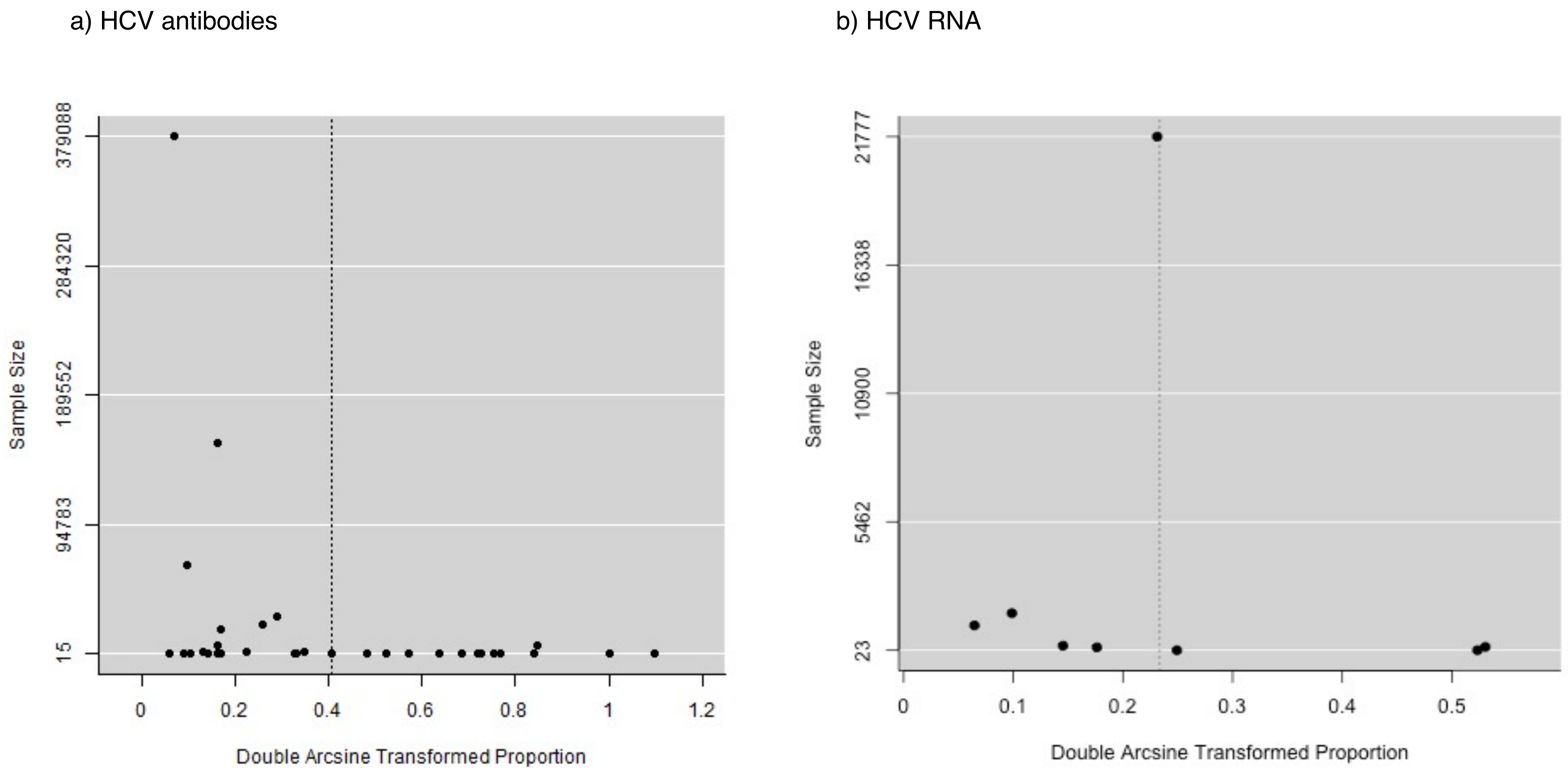

Egger Test for Funnel Plot Asymmetry:
z = -3.8213,
p = 0.0001

Egger Test for Funnel Plot Asymmetry:
z = -0.2030,
p = 0.8392

Figure 6: Funnel plot of HCV prevalence a) HCV antibodies b) HCV RNA

Supporting file 1

Click here to access/download
Supporting Information
Supporting file1_PRISMA checklist.docx

Supporting file 2

Click here to access/download
Supporting Information
Supporting file2_Supplementary TableS1S2.docx
