# Supplementary material for: Intermediate hepatitis C virus (HCV) endemicity and its genotype distribution in Myanmar: A systematic review and meta-analysis
Source: PLoS One. 2024 Sep 19;19(9):e0307872. doi: 10.1371/journal.pone.0307872 (PMC11412534; doi:10.1371/journal.pone.0307872)
Supplement: Attachment — Submitted filename: 240617 Response to ReviewersV2.pdf [file pone.0307872.s006.pdf]

Response to Reviewer Comments

We wish to express our appreciation to the editor, and reviewers for their additional comments on our paper. We revised our paper based on the editor and reviewer's comment as below.

Response to Reviewer-1

Comment-1: The study was not registered in a prospective systematic review register such as PROSPERO. Have authors confirmed that there are no similar ongoing studies? If so, it should be clearly stated.

Response-1: Thank you so much for your comment. We acknowledge that we did not register the protocol in a systematic review registry. But, before the review, we searched databases such as PubMed, Web of Science, and Scopus for published studies on this topic and found none in PROSPERO, INPLASY, etc. We have clarified this in the manuscript (Page 4, Line 94-98) as below.

"Though we did not prospectively register our systematic review in the registry database such as International Prospective Register of Systematic Reviews (PROSPERO), International Platform of Registered Systematic Review and Meta-analysis Protocols (INPLASY) etc, there was no ongoing similar studies in those databases. Before the review, we searched databases such as PubMed, Web of Science, and Scopus for published studies on this topic and found none."

Comment-2: Authors should indicate which are 'studies' and 'reports' from the cited references in Table 1.

Response-2: We appreciate very much for your valuable comments. We added the symbol "^ in the studies issued from the same report" in the Table 1 and mentioned in the footnote.

Comment-3. Authors should consider performing a sensitivity analysis excluding unpublish data in groups where they occur.

Response-3: Thank you for your comment and important suggestion. We performed a sensitivity analysis by excluding unpublished data. All unpublished data were in low-risk groups only. In the initial analysis, the prevalence of HCV in low-risk groups was 2.18% [1.09% - 3.58%]. After excluding unpublished data, the prevalence increased to 2.41% [0.51%; 5.40%] in the low-risk group. This difference is not statistically significant (please see below). We have included the results and discussion of the sensitivity analysis in the manuscript, but we decided not to include the calculation result figure.

(Result: Page 12, Line 218-220)
"A sensitivity analysis indicated that the prevalence of HCV antibodies increased to 2.41% (95% CI: 0.51%-5.4%) after excluding 3 unpublished reports (n=72,680). However, this difference was not statistically significant (the result not shown)."

(Discussion: Page 17, Line 322-325)
"Despite the sensitivity analysis showing a slight increase in HCV antibody prevalence after removing the unpublished reports in low-risk groups, the change was not statistically significant. However, caution should be exercised when interpreting the pooled prevalence estimates."

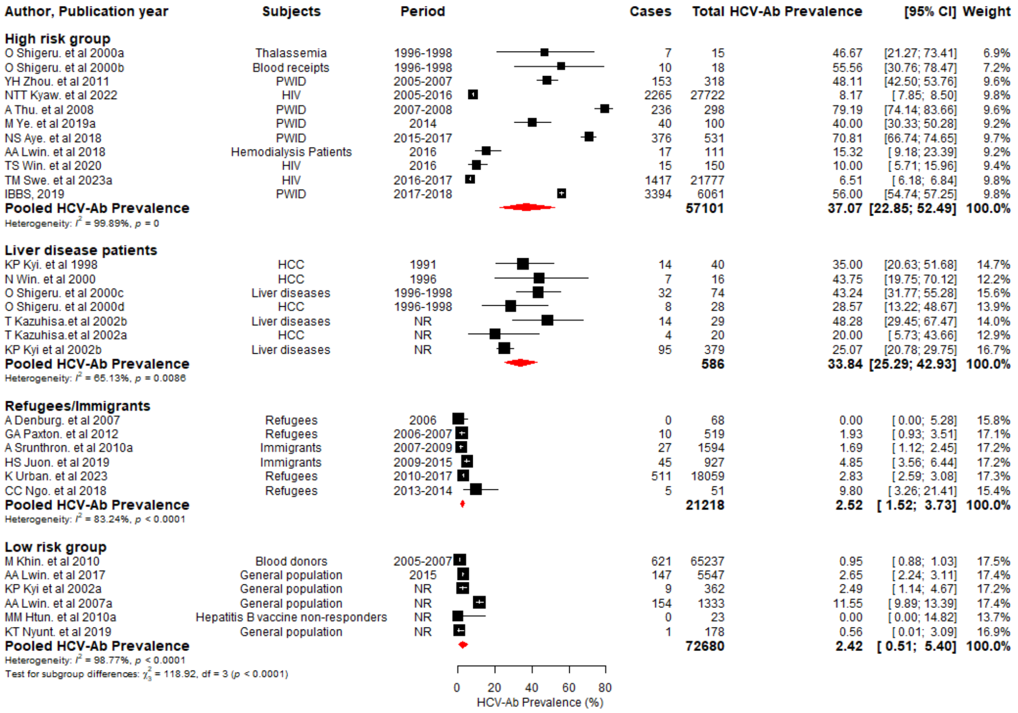

Comment-4: Line 272: How are the genotype distribution 'same' based on the proportion values provided in the sentence? Maybe 'similar'? Please clarify.

Response-4: Thank you so much for your clarification. It was our mistake, and we corrected as "similar". We have revised in the result section accordingly.

Response to Reviewer-2

Comment-1: The abstract should be rewritten stating the background, objective of the study. In the Methods, how data was extracted, and the persons involved. Also, the conclusion should reflect the answer to the main objective of the work.

Response-1: Thank you very much for your comments, we revised the abstract according to your suggestion. As we had provided the information of person involved in the main manuscript, please let us not include in the abstract.

Comment-2: In the Background section; The first paragraph should include what is HCV, before giving the prevalence. Also, Paragraph 2 is not necessary. Paragraph 3 and 4 should be rewritten, including more references, and stating clearly the importance of the work before stating the objectives.

Response-2: We appreciate very much for your valuable comments. Based on your suggestions, we deleted paragraph 2, added more references supporting the facts in background and clarified and justified the study aims based on our research title respectively.

Comment-3: In the Material and methods section: All the sentences starting with To, should be rephrased. I have highlighted in the manuscript.

Response-3: Thank you very much for your intensive review. Based on your suggestion, we revised all the statements started with "To" appropriately. We appreciate very much for your valuable comments.

Comment-4: In the results section; I have highlighted in the manuscript, Some sentences should be rephrased, starting appropriately.

Response-4: Thank you very much for your valuable suggestion, we have rephrased them accordingly.

Comment-5: In the Discussion section, Same comments as the results section.

Response-5: Thank you very much for your insightful suggestion, we rephrased and revised the statement as you commented details in the manuscript.

Comment-6: Conclusion is Ok.

Response-6: Thank you very much for your comment, we appreciate.

Comment-7: References: All the references should be rearranged and harmonised.

Response-7: Thank you very much for your comment and all the references were also harmonized according to the journal's requirement.

Response to the editorial office

Comment-1. Please ensure that your manuscript meets PLOS ONE's style requirements, including those for file naming. The PLOS ONE style templates can be found at https://journals.plos.org/plosone/s/file?id=wjVg/PLOSOne_formatting_sample_main_body.pdf and https://journals.plos.org/plosone/s/file?id=ba62/PLOSOne_formatting_sample_title_authors_affiliations.pdf

Response-1: Thank you very much for your comments, we formatted the manuscript based on the instruction.

Comment-2. We suggest you thoroughly copyedit your manuscript for language usage, spelling, and grammar. If you do not know anyone who can help you do this, you may wish to consider employing a professional scientific editing service.
The American Journal Experts (AJE) (https://www.aje.com/) is one such service that has extensive experience helping authors meet PLOS guidelines and can provide language editing, translation, manuscript formatting, and figure formatting to ensure your manuscript meets our submission guidelines. Please note that having the manuscript copyedited by AJE or any other editing services does not guarantee selection for peer review or acceptance for publication.
Upon resubmission, please provide the following:
The name of the colleague or the details of the professional service that edited your manuscript
*A copy of your manuscript showing your changes by either highlighting them or using track changes (uploaded as a *supporting information* file)*
*A clean copy of the edited manuscript (uploaded as the new *manuscript* file)*

Response-2: Thank you very much for your comments, ZP and KK, the first and coauthor of this manuscript did the intensive edition, and we provided the revised version in both clean and with tract change.

Comment-3. Please include a complete copy of PLOS' questionnaire on inclusivity in global research in your revised manuscript. Our policy for research in this area aims to improve transparency in the reporting of research performed outside of researchers' own country or community. The policy applies to researchers who have travelled to a different country to conduct research, research with Indigenous populations or their lands, and research on cultural artefacts. The questionnaire can also be requested at the journal's discretion for any other submissions, even if these conditions are not met. Please find more information on the policy and a link to download a blank copy of the questionnaire here: https://journals.plos.org/plosone/s/best-practices-in-research-reporting. Please upload a completed version of your questionnaire as Supporting Information when you resubmit your manuscript.

Response-3: Thank you very much for your comments, we provided the checklist for the Inclusivity in global research questionnaire as Supporting Information and mentioned in the method section with sub-heading (Page 6, Line 150-152).

"Inclusivity in global research: Additional information regarding the ethical, cultural, and scientific considerations specific to inclusivity in global research is included in the Supporting Information (S4 Checklist)."

Comment-4. Thank you for stating the following financial disclosure:
"This work was supported by Japan's Ministry of Health, Labour and Welfare (MHLW) Policy Research for Hepatitis Measures Program Grant Number JPMH22HC1001."
Please state what role the funders took in the study. If the funders had no role, please state: "The funders had no role in study design, data collection and analysis, decision to publish, or preparation of the manuscript."
If this statement is not correct you must amend it as needed.
Please include this amended Role of Funder statement in your cover letter; we will change the online submission form on your behalf.

Response-4: Thank you very much for your comments, we added this funder role statement in the manuscript and in the cover letter. Using this opportunity, we would like to change the funding source as "This work was supported by Japan Society for the Promotion of Science (JSPS) Core-to-Core Program (Grant number: JPJSCCB20240009). The funders had no role in study design, data collection and analysis, decision to publish, or preparation of the manuscript".

Comment-5. We note that Figure 5 in your submission contain [map/satellite] images which may be copyrighted. All PLOS content is published under the Creative Commons Attribution License (CC BY 4.0), which means that the manuscript, images, and Supporting Information files will be freely available online, and any third party is permitted to access, download, copy, distribute, and use these materials in any way, even commercially, with proper attribution. For these reasons, we cannot publish previously copyrighted maps or satellite images created using proprietary data, such as Google software (Google Maps, Street View, and Earth). For more information, see our copyright guidelines: http://journals.plos.org/plosone/s/licenses-and-copyright.

We require you to either (1) present written permission from the copyright holder to publish these figures specifically under the CC BY 4.0 license, or (2) remove the figures from your submission:
a. You may seek permission from the original copyright holder of Figure 5 to publish the content specifically under the CC BY 4.0 license.
We recommend that you contact the original copyright holder with the Content Permission Form (http://journals.plos.org/plosone/s/file?id=7c09/content-permission-form.pdf) and the following text:
"I request permission for the open-access journal PLOS ONE to publish XXX under the Creative Commons Attribution License (CCAL) CC BY 4.0 (http://creativecommons.org/licenses/by/4.0/). Please be aware that this license allows unrestricted use and distribution, even commercially, by third parties. Please reply and provide explicit written permission to publish XXX under a CC BY license and complete the attached form."
Please upload the completed Content Permission Form or other proof of granted permissions as an "Other" file with your submission.
In the figure caption of the copyrighted figure, please include the following text: "Reprinted from [ref] under a CC BY license, with permission from [name of publisher], original copyright [original copyright year]."
b. If you are unable to obtain permission from the original copyright holder to publish these figures under the CC BY 4.0 license or if the copyright holder's requirements are incompatible with the CC BY 4.0 license, please either i) remove the figure or ii) supply a replacement figure that complies with the CC BY 4.0 license. Please check copyright information on all replacement figures and update the figure caption with source information. If applicable, please specify in the figure caption text when a figure is similar but not identical to the original image and is therefore for illustrative purposes only.
The following resources for replacing copyrighted map figures may be helpful:
USGS National Map Viewer (public domain): http://viewer.nationalmap.gov/viewer/
The Gateway to Astronaut Photography of Earth (public domain): http://eol.jsc.nasa.gov/sseop/clickmap/
Maps at the CIA (public domain): https://www.cia.gov/library/publications/the-world-factbook/index.html and https://www.cia.gov/library/publications/cia-maps-publications/index.html
NASA Earth Observatory (public domain): http://earthobservatory.nasa.gov/
Landsat: http://landsat.visibleearth.nasa.gov/
USGS EROS (Earth Resources Observatory and Science (EROS) Center) (public domain): http://eros.usgs.gov/#
Natural Earth (public domain): http://www.naturalearthdata.com/

Response-5: Thank you very much for your comments. In fact, we created the map by ourselves using the free online based creation at http://n.freemap.jp. Therefore, no copyright issue to use the map from that abovementioned free webpage. But, for the printing of the colored map what we created, the copyright belongs to us.
