# Supplementary material for: Intermediate hepatitis C virus (HCV) endemicity and its genotype distribution in Myanmar: A systematic review and meta-analysis
Source: PLoS One. 2024 Sep 19;19(9):e0307872. doi: 10.1371/journal.pone.0307872 (PMC11412534; doi:10.1371/journal.pone.0307872)
Supplement: Attachment — Submitted filename: PONE-D-24-10345_R1 Revised DrZeukoo.pdf [file pone.0307872.s007.pdf]

PLOS ONE

Intermediate hepatitis C virus (HCV) endemicity and its genotype distribution in Myanmar: A systematic review and meta-analysis

--Manuscript Draft--

**Manuscript Number:**	PONE-D-24-10345R1
**Article Type:**	Research Article
**Full Title:**	Intermediate hepatitis C virus (HCV) endemicity and its genotype distribution in Myanmar: A systematic review and meta-analysis
**Short Title:**	Hepatitis C in Myanmar
**Corresponding Author:**	Junko Tanaka, Ph.D.<br>Hiroshima University Faculty of Medicine Graduate School of Biomedical and Health Sciences: Hiroshima Daigaku Igakubu Daigakuin Ishiyaku Hokengaku Kenkyuka<br>Hiroshima, JAPAN
**Keywords:**	Seroprevalence; hepatitis; HCV; HCV-Ab; HCV RNA; HCV genotype; Myanmar; Burma; Systematic review; Meta-analysis.
**Abstract:**	Comprehensive details on Hepatitis C virus (HCV) infection in Myanmar are lacking. This study determined the prevalence of HCV antibodies and ribonucleic acid (RNA) and the distribution of HCV genotypes across different populations in Myanmar from 1990 to 2023. A systematic search in PubMed, Web of Science, Scopus, and local journals identified studies reporting on HCV antibodies, RNA, and genotypes, excluding clinical research related to liver disease prognosis. Screening and data extraction was done by two authors and study populations were categorized into low-risk, high-risk, liver disease patients, and refugees outside the country. The pooled prevalence was performed by Dersimonian and Laird method using the R program. The publication bias was shown by funnel plot, the Egger test was used to assess the symmetry of the plot, and the heterogeneity was examined by the Cochran Q test and I2 index. Out of 135 reports screened for eligibility, 35 reports comprising 51 studies were included in which 33 studies provided data on HCV seroprevalence in 685,403 individuals, 8 studies reported HCV RNA prevalence in 25,018 individuals, and 10 studies examined HCV genotypes in 1,845 individuals. The pooled seroprevalence of HCV among low-risk, high-risk, liver disease patients and refugees were 2.18%, 37.07%, 33.84%, and 2.52% respectively. HCV RNA-positive rates in these groups were 1.40%, 5.25%, 24.96%, and 0.84% respectively. Seroprevalence studies showed publication bias (Egger test, p=0.0001), while RNA studies did not (Egger test, p=0.8392). HCV genotype 3 was predominant in all sub-groups in Myanmar. Our study shows Myanmar has intermediate HCV endemicity with lowest HCV prevalence of 2.18% in low-risk groups and highest prevalence of 37.07% in high- risk groups. However, the findings highlight the need for further epidemiological studies to understand actual disease burden and implement effective countermeasures to achieve the WHO's goal of HCV elimination by 2030.
**Order of Authors:**	Zayar Phyo
	Ko Ko
	Serge Ouoba
	Aya Sugiyama
	Ulugbek Khudayberdievich Mirzaev
	Golda Ataa Akuffo
	Chanroth Chhoung
	Tomoyuki Akita
	Junko Tanaka, Ph.D.
**Response to Reviewers:**	Response to Reviewer Comments<br>We wish to express our appreciation to the editor, and reviewers for their additional comments on our paper. We revised our paper based on the editor and reviewer's

Powered by Editorial Manager® and ProduXion Manager® from Aries Systems Corporation

comment as below.

Response to Reviewer-1

Comment-1: The study was not registered in a prospective systematic review register such as PROSPERO. Have authors confirmed that there are no similar ongoing studies? If so, it should be clearly stated.

Response-1: Thank you so much for your comment. We acknowledge that we did not register the protocol in a systematic review registry. But, before the review, we searched databases such as PubMed, Web of Science, and Scopus for published studies on this topic and found none in PROSPERO, INPLASY, etc. We have clarified this in the manuscript (Page 4, Line 94-98) as below.

“Though we did not prospectively register our systematic review in the registry database such as International Prospective Register of Systematic Reviews (PROSPERO), International Platform of Registered Systematic Review and Meta-analysis Protocols (INPLASY) etc, there was no ongoing similar studies in those databases. Before the review, we searched databases such as PubMed, Web of Science, and Scopus for published studies on this topic and found none.”

Comment-2: Authors should indicate which are ‘studies’ and ‘reports’ from the cited references in Table 1.

Response-2: We appreciate very much for your valuable comments. We added the symbol “^ in the studies issued from the same report” in the Table 1 and mentioned in the footnote.

Comment-3. Authors should consider performing a sensitivity analysis excluding unpublish data in groups where they occur.

Response-3: Thank you for your comment and important suggestion. We performed a sensitivity analysis by excluding unpublished data. All unpublished data were in low-risk groups only. In the initial analysis, the prevalence of HCV in low-risk groups was 2.18% [1.09% - 3.58%]. After excluding unpublished data, the prevalence increased to 2.41% [0.51%; 5.40%] in the low-risk group. This difference is not statistically significant (please see below). We have included the results and discussion of the sensitivity analysis in the manuscript, but we decided not to include the calculation result figure.

(Result: Page 12, Line 218-220)
“A sensitivity analysis indicated that the prevalence of HCV antibodies increased to 2.41% (95% CI: 0.51%-5.4%) after excluding 3 unpublished reports (n=72,680). However, this difference was not statistically significant (the result not shown).”

(Discussion: Page 17, Line 322-325)
“Despite the sensitivity analysis showing a slight increase in HCV antibody prevalence after removing the unpublished reports in low-risk groups, the change was not statistically significant. However, caution should be exercised when interpreting the pooled prevalence estimates.”

Comment-4: Line 272: How are the genotype distribution ‘same’ based on the proportion values provided in the sentence? Maybe ‘similar’? Please clarify.

Response-4: Thank you so much for your clarification. It was our mistake, and we corrected as “similar”. We have revised in the result section accordingly.

Response to Reviewer-2

Comment-1: The abstract should be rewritten stating the background, objective of the study. In the Methods, how data was extracted, and the persons involved. Also, the conclusion should reflect the answer to the main objective of the work.

Powered by Editorial Manager® and ProduXion Manager® from Aries Systems Corporation

Response-1: Thank you very much for your comments, we revised the abstract according to your suggestion. As we had provided the information of person involved in the main manuscript, please let us not include in the abstract.

Comment-2: In the Background section; The first paragraph should include what is HCV, before giving the prevalence. Also, Paragraph 2 is not necessary. Paragraph 3 and 4 should be rewritten, including more references, and stating clearly the importance of the work before stating the objectives.

Response-2: We appreciate very much for your valuable comments. Based on your suggestions, we deleted paragraph 2, added more references supporting the facts in background and clarified and justified the study aims based on our research title respectively.

Comment-3: In the Material and methods section: All the sentences starting with To, should be rephrased. I have highlighted in the manuscript.

Response-3: Thank you very much for your intensive review. Based on your suggestion, we revised all the statements started with “To” appropriately. We appreciate very much for your valuable comments.

Comment-4: In the results section; I have highlighted in the manuscript, Some sentences should be rephrased, starting appropriately.

Response-4: Thank you very much for your valuable suggestion, we have rephrased them accordingly.

Comment-5: In the Discussion section, Same comments as the results section.

Response-5: Thank you very much for your insightful suggestion, we rephrased and revised the statement as you commented details in the manuscript.

Comment-6: Conclusion is Ok.

Response-6: Thank you very much for your comment, we appreciate.

Comment-7: References: All the references should be rearranged and harmonised.

Response-7: Thank you very much for your comment and all the references were also harmonized according to the journal’s requirement.

Response to the editorial office

Comment-1. Please ensure that your manuscript meets PLOS ONE's style requirements, including those for file naming. The PLOS ONE style templates can be found at
https://journals.plos.org/plosone/s/file?id=wjVg/PLOSOne_formatting_sample_main_body.pdf and
https://journals.plos.org/plosone/s/file?id=ba62/PLOSOne_formatting_sample_title_authors_affiliations.pdf

Response-1: Thank you very much for your comments, we formatted the manuscript based on the instruction.

Comment-2. We suggest you thoroughly copyedit your manuscript for language usage, spelling, and grammar. If you do not know anyone who can help you do this, you may wish to consider employing a professional scientific editing service.
The American Journal Experts (AJE) (https://www.aje.com/) is one such service that has extensive experience helping authors meet PLOS guidelines and can provide language editing, translation, manuscript formatting, and figure formatting to ensure your manuscript meets our submission guidelines. Please note that having the manuscript copyedited by AJE or any other editing services does not guarantee

selection for peer review or acceptance for publication.
Upon resubmission, please provide the following:
The name of the colleague or the details of the professional service that edited your manuscript
A copy of your manuscript showing your changes by either highlighting them or using track changes (uploaded as a *supporting information* file)
A clean copy of the edited manuscript (uploaded as the new *manuscript* file)

Response-2: Thank you very much for your comments, ZP and KK, the first and coauthor of this manuscript did the intensive edition, and we provided the revised version in both clean and with tract change.

Comment-3. Please include a complete copy of PLOS' questionnaire on inclusivity in global research in your revised manuscript. Our policy for research in this area aims to improve transparency in the reporting of research performed outside of researchers' own country or community. The policy applies to researchers who have travelled to a different country to conduct research, research with Indigenous populations or their lands, and research on cultural artefacts. The questionnaire can also be requested at the journal's discretion for any other submissions, even if these conditions are not met. Please find more information on the policy and a link to download a blank copy of the questionnaire here: https://journals.plos.org/plosone/s/best-practices-in-research-reporting. Please upload a completed version of your questionnaire as Supporting Information when you resubmit your manuscript.

Response-3: Thank you very much for your comments, we provided the checklist for the Inclusivity in global research questionnaire as Supporting Information and mentioned in the method section with sub-heading (Page 6, Line 150-152).
"Inclusivity in global research: Additional information regarding the ethical, cultural, and scientific considerations specific to inclusivity in global research is included in the Supporting Information (S4 Checklist)."

Comment-4. Thank you for stating the following financial disclosure:
"This work was supported by Japan's Ministry of Health, Labour and Welfare (MHLW) Policy Research for Hepatitis Measures Program Grant Number JPMH22HC1001."
Please state what role the funders took in the study. If the funders had no role, please state: "The funders had no role in study design, data collection and analysis, decision to publish, or preparation of the manuscript."
If this statement is not correct you must amend it as needed.
Please include this amended Role of Funder statement in your cover letter; we will change the online submission form on your behalf.

Response-4: Thank you very much for your comments, we added this funder role statement in the manuscript and in the cover letter. Using this opportunity, we would like to change the funding source as "This work was supported by Japan Society for the Promotion of Science (JSPS) Core-to-Core Program (Grant number: JPJSCCB20240009). The funders had no role in study design, data collection and analysis, decision to publish, or preparation of the manuscript".

Comment-5. We note that Figure 5 in your submission contain [map/satellite] images which may be copyrighted. All PLOS content is published under the Creative Commons Attribution License (CC BY 4.0), which means that the manuscript, images, and Supporting Information files will be freely available online, and any third party is permitted to access, download, copy, distribute, and use these materials in any way, even commercially, with proper attribution. For these reasons, we cannot publish previously copyrighted maps or satellite images created using proprietary data, such as Google software (Google Maps, Street View, and Earth). For more information, see our copyright guidelines: http://journals.plos.org/plosone/s/licenses-and-copyright.
We require you to either (1) present written permission from the copyright holder to publish these figures specifically under the CC BY 4.0 license, or (2) remove the figures from your submission:
a. You may seek permission from the original copyright holder of Figure 5 to publish the content specifically under the CC BY 4.0 license.
We recommend that you contact the original copyright holder with the Content

	Permission Form (http://journals.plos.org/plosone/s/file?id=7c09/content-permission-form.pdf) and the following text:<br>“I request permission for the open-access journal PLOS ONE to publish XXX under the Creative Commons Attribution License (CCAL) CC BY 4.0 (http://creativecommons.org/licenses/by/4.0/). Please be aware that this license allows unrestricted use and distribution, even commercially, by third parties. Please reply and provide explicit written permission to publish XXX under a CC BY license and complete the attached form.”<br>Please upload the completed Content Permission Form or other proof of granted permissions as an "Other" file with your submission.<br>In the figure caption of the copyrighted figure, please include the following text: “Reprinted from [ref] under a CC BY license, with permission from [name of publisher], original copyright [original copyright year].”<br>b. If you are unable to obtain permission from the original copyright holder to publish these figures under the CC BY 4.0 license or if the copyright holder’s requirements are incompatible with the CC BY 4.0 license, please either i) remove the figure or ii) supply a replacement figure that complies with the CC BY 4.0 license. Please check copyright information on all replacement figures and update the figure caption with source information. If applicable, please specify in the figure caption text when a figure is similar but not identical to the original image and is therefore for illustrative purposes only.<br>The following resources for replacing copyrighted map figures may be helpful:<br>USGS National Map Viewer (public domain): http://viewer.nationalmap.gov/viewer/<br>The Gateway to Astronaut Photography of Earth (public domain): http://eol.jsc.nasa.gov/sseop/clickmap/<br>Maps at the CIA (public domain): https://www.cia.gov/library/publications/the-world-factbook/index.html and https://www.cia.gov/library/publications/cia-maps-publications/index.html<br>NASA Earth Observatory (public domain): http://earthobservatory.nasa.gov/<br>Landsat: http://landsat.visibleearth.nasa.gov/<br>USGS EROS (Earth Resources Observatory and Science (EROS) Center) (public domain): http://eros.usgs.gov/#<br>Natural Earth (public domain): http://www.naturalearthdata.com/<br><br>Response-5: Thank you very much for your comments. In fact, we created the map by ourselves using the free online based creation at http://n.freemap.jp. Therefore, no copyright issue to use the map from that abovementioned free webpage. But, for the printing of the colored map what we created, the copyright belongs to us.
**Additional Information:**	
**Question**	**Response**
**Financial Disclosure**<br><br>Enter a financial disclosure statement that describes the sources of funding for the work included in this submission. Review the submission guidelines for detailed requirements. View published research articles from *PLOS ONE* for specific examples.<br><br>This statement is required for submission and **will appear in the published article** if the submission is accepted. Please make sure it is accurate.	Yes

**Funded studies**<br>Enter a statement with the following details:<br>• Initials of the authors who received each award<br>• Grant numbers awarded to each author<br>• The full name of each funder<br>• URL of each funder website<br>• Did the sponsors or funders play any role in the study design, data collection and analysis, decision to publish, or preparation of the manuscript?<br><br>Did you receive funding for this work?	
Please add funding details.<br>as follow-up to "**Financial Disclosure**<br><br>Enter a financial disclosure statement that describes the sources of funding for the work included in this submission. Review the submission guidelines for detailed requirements. View published research articles from *PLOS ONE* for specific examples.<br><br>This statement is required for submission and **will appear in the published article** if the submission is accepted. Please make sure it is accurate.<br><br>**Funded studies**<br>Enter a statement with the following details:<br>• Initials of the authors who received each award<br>• Grant numbers awarded to each author<br>• The full name of each funder<br>• URL of each funder website<br>• Did the sponsors or funders play any role in the study design, data collection and analysis, decision to publish, or preparation of the manuscript?<br><br>Did you receive funding for this work?"	This work was supported by Japan’s Ministry of Health, Labour and Welfare (MHLW) Policy Research for Hepatitis Measures Program Grant Number JPMH22HC1001.
Please select the country of your main research funder (please select carefully as in some cases this is used in fee calculation).	JAPAN - JP

as follow-up to "**Financial Disclosure**<br><br>Enter a financial disclosure statement that describes the sources of funding for the work included in this submission. Review the submission guidelines for detailed requirements. View published research articles from *PLOS ONE* for specific examples.<br><br>This statement is required for submission and **will appear in the published article** if the submission is accepted. Please make sure it is accurate.<br><br>**Funded studies**<br>Enter a statement with the following details:<br>• Initials of the authors who received each award<br>• Grant numbers awarded to each author<br>• The full name of each funder<br>• URL of each funder website<br>• Did the sponsors or funders play any role in the study design, data collection and analysis, decision to publish, or preparation of the manuscript?<br><br>Did you receive funding for this work?"	
**Competing Interests**<br><br>Use the instructions below to enter a competing interest statement for this submission. On behalf of all authors, disclose any competing interests that could be perceived to bias this work—acknowledging all financial support and any other relevant financial or non-financial competing interests.<br><br>This statement is required for submission and **will appear in the published article** if the submission is accepted. Please make sure it is accurate and that any funding sources listed in your Funding Information later in the submission form are also declared in your Financial Disclosure statement.	The authors declare no conflict of interest.

View published research articles from *PLOS ONE* for specific examples.<br><br>**NO authors have competing interests**<br><br>Enter: *The authors have declared that no competing interests exist.*<br><br>**Authors with competing interests**<br><br>Enter competing interest details beginning with this statement:<br><br>*I have read the journal's policy and the authors of this manuscript have the following competing interests: [insert competing interests here]*<br><br>* typeset	
**Ethics Statement**<br><br>Enter an ethics statement for this submission. This statement is required if the study involved:<br><br>• Human participants<br>• Human specimens or tissue<br>• Vertebrate animals or cephalopods<br>• Vertebrate embryos or tissues<br>• Field research<br><br>Write "N/A" if the submission does not require an ethics statement.<br><br>General guidance is provided below. Consult the submission guidelines for detailed instructions. **Make sure that all information entered here is included in the Methods section of the manuscript.**	Not applicable.

**Format for specific study types**<br><br>**Human Subject Research (involving human participants and/or tissue)**<br>• Give the name of the institutional review board or ethics committee that approved the study<br>• Include the approval number and/or a statement indicating approval of this research<br>• Indicate the form of consent obtained (written/oral) or the reason that consent was not obtained (e.g. the data were analyzed anonymously)<br><br>**Animal Research (involving vertebrate animals, embryos or tissues)**<br>• Provide the name of the Institutional Animal Care and Use Committee (IACUC) or other relevant ethics board that reviewed the study protocol, and indicate whether they approved this research or granted a formal waiver of ethical approval<br>• Include an approval number if one was obtained<br>• If the study involved *non-human primates*, add *additional details* about animal welfare and steps taken to ameliorate suffering<br>• If anesthesia, euthanasia, or any kind of animal sacrifice is part of the study, include briefly which substances and/or methods were applied<br><br>**Field Research**<br><br>Include the following details if this study involves the collection of plant, animal, or other materials from a natural setting:<br>• Field permit number<br>• Name of the institution or relevant body that granted permission	
**Data Availability**<br><br>Authors are required to make all data underlying the findings described fully available, without restriction, and from the time of publication. PLOS allows rare exceptions to address legal and ethical concerns. See the PLOS Data Policy and FAQ for detailed information.	Yes - all data are fully available without restriction

Powered by Editorial Manager® and ProduXion Manager® from Aries Systems Corporation

A Data Availability Statement describing where the data can be found is required at submission. Your answers to this question constitute the Data Availability Statement and **will be published in the article**, if accepted.<br><br>**Important:** Stating 'data available on request from the author' is not sufficient. If your data are only available upon request, select 'No' for the first question and explain your exceptional situation in the text box.<br><br>Do the authors confirm that all data underlying the findings described in their manuscript are fully available without restriction?	
**Describe where the data may be found in full sentences. If you are copying our sample text, replace any instances of XXX with the appropriate details.**<br><br>• If the data are **held or will be held in a public repository**, include URLs, accession numbers or DOIs. If this information will only be available after acceptance, indicate this by ticking the box below. For example: *All XXX files are available from the XXX database (accession number(s) XXX, XXX.).*<br>• If the data are all contained **within the manuscript and/or Supporting Information files**, enter the following: *All relevant data are within the manuscript and its Supporting Information files.*<br>• If neither of these applies but you are able to provide **details of access elsewhere**, with or without limitations, please do so. For example:<br><br>*Data cannot be shared publicly because of [XXX]. Data are available from the XXX Institutional Data Access / Ethics Committee (contact via XXX) for researchers who meet the criteria for access to confidential data.*<br><br>*The data underlying the results presented in the study are available from (include the name of the third party*	All relevant data are within the manuscript and its Supporting Information files.

Powered by Editorial Manager® and ProduXion Manager® from Aries Systems Corporation

and contact information or URL).<br>• This text is appropriate if the data are owned by a third party and authors do not have permission to share the data.<br><br>* typeset	
Additional data availability information:	

Powered by Editorial Manager® and ProduXion Manager® from Aries Systems Corporation

Manuscript

Click here to access/download;Manuscript;240617 ManuscriptV2.docx

Intermediate hepatitis C virus (HCV) endemicity and its genotype distribution in Myanmar: A systematic review and meta-analysis

Zayar Phyo[1,2], Ko Ko[1,2], Serge Ouoba[1,2,3], Aya Sugiyama[1,2], Ulugbek Khudayberdievich Mirzaev[1,2,4], Golda Ataa Akuffo[1,2], Chanroth Chhoung[1,2], Tomoyuki Akita[1,2], Junko Tanaka[1,2*]

[1]Department of Epidemiology, Infectious Disease Control and Prevention, Graduate School of Biomedical and Health Science, Hiroshima University, Hiroshima, Japan

[2]Project Research Center for Epidemiology and Prevention of viral hepatitis and hepatocellular carcinoma, Hiroshima University, Hiroshima, Japan

[3]Unité de Recherche Clinique de Nanoro (URCN), Institut de Recherche en Science de la Santé (IRSS), Nanoro, Burkina Faso

[4]Department of Hepatology, Scientific Research Institute of Virology, Tashkent, Uzbekistan

*Corresponding Author

Email: jun-tanaka@hiroshima-u.ac.jp (JT)

Abstract

Comprehensive details on Hepatitis C virus (HCV) infection in Myanmar are lacking. This study determined the prevalence of HCV antibodies and ribonucleic acid (RNA) and the distribution of HCV genotypes across different populations in Myanmar from 1990 to 2023. A systematic search in PubMed, Web of Science, Scopus, and local journals identified studies reporting on HCV antibodies, RNA, and genotypes, excluding clinical research related to liver disease prognosis. Screening and data extraction was done by two authors and study populations were categorized into low-risk, high-risk, liver disease patients, and refugees outside the country. The pooled prevalence was performed by Dersimonian and Laird method using the R program. The publication bias was shown by funnel plot, the Egger test was used to assess the symmetry of the plot, and the heterogeneity was examined by the Cochran Q test and I^2 index. Out of 135 reports screened for eligibility, 35 reports comprising 51 studies were included in which 33 studies provided data on HCV seroprevalence in 685,403 individuals, 8 studies reported HCV RNA prevalence in 25,018 individuals, and 10 studies examined HCV genotypes in 1,845 individuals. The pooled seroprevalence of HCV among low-risk, high-risk, liver disease patients and refugees were 2.18%, 37.07%, 33.84%, and 2.52% respectively. HCV RNA-positive rates in these groups were 1.40%, 5.25%, 24.96%, and 0.84% respectively. Seroprevalence studies showed publication bias (Egger test, p=0.0001), while RNA studies did not (Egger test, p=0.8392). HCV genotype 3 was predominant in all sub-groups in Myanmar. Our study shows Myanmar has intermediate HCV endemicity with lowest HCV prevalence of 2.18% in low-risk groups and highest prevalence of 37.07% in high-risk groups. However, the findings highlight the need for further epidemiological studies to understand actual disease burden and implement effective countermeasures to achieve the WHO's goal of HCV elimination by 2030.

Keywords: Seroprevalence, Hepatitis, HCV, HCV-Ab, HCV RNA, HCV genotype, Myanmar, Burma,

Systematic review, Meta-analysis.

Background

Hepatitis C virus (HCV) is an enveloped positive-strand RNA virus belonging to the genus Hepacivirus in the family Flaviviridae. It causes liver inflammation, which can range from mild to severe, including cirrhosis and liver cancer. HCV is a bloodborne virus transmitted through exposure to infected blood, which can occur via unsafe injection practices, unsafe healthcare, unscreened blood transfusions, injection drug use, and sexual practices that involve contact with contaminated blood [1]. Globally, 58 million people are infected by HCV infection with approximately 1.5 million new infections each year [1]. According to the World Health Organization (WHO), only 20% of those infected with HCV have been diagnosed, leaving a significant number undiagnosed cases because of limited access to affordable testing [2]. The imperceptible barriers were still present in line with the elimination of viral hepatitis as a global action, rising as a public health concern. As for WHO Southeast Asia region in 2020, there was an estimated 10 million people living with chronic hepatitis C, resulting in 40,938 deaths annually and less than 1% of diagnosed viremic patients had started treatment [3].

Myanmar, a developing country in Southeast Asia, is rich in natural resources and home to a diverse population of 54.78 million people from various ethnic groups. Before 2017, there was no national program or strategic plan in Myanmar providing free-of-charge treatment for HCV. Although HCV antibodies and HCV RNA testing were available in private hospitals and laboratories, they are costly [4]. With the introduction of direct-acting antivirals (DAAs) which have a high success rate (>90% sustained virologic response: SVR) and fewer side effects, the Myanmar government initiated the free testing and antiviral treatment in seven public hospitals in 2017 [5]. This made Myanmar the first country in Southeast Asia region providing free HCV treatment. Various local and international non-governmental organizations have also joined efforts to eliminate HCV in Myanmar [6]. The Myanmar Liver Foundation (MLF) has been offering anti-HCV treatment to patients with chronic liver diseases through their Than Sitt Charity Clinics since 2017 [7]. Médecins Sans Frontières (MSF) has been providing anti-HCV treatment specifically to individuals co-infected with HIV in Myanmar since the same year [8]. Additionally, the Burnet Institute of Myanmar has been delivering HCV treatment to both the general population and people who inject drugs (PWID) since 2019 [6].

The epidemiological patterns of HCV infection in Myanmar are not well-documented. Between 1996 and 2017, reported prevalence rates of HCV antibodies varied widely, from the lowest 0.50% among blood donors (from an unpublished study) to the highest 79.19% [9] among people who inject drugs (PWID). A nationwide study in 2015 estimated the prevalence of HCV antibodies at 2.65% [10], suggesting that approximately 1.4 million

people had been exposed to HCV. Given that about 30% of those infected naturally eliminate the virus within six months without treatment [1], the total number of chronic HCV infections was estimated to be around 0.9 million. These chronic infections can progress to severe complications such as liver cirrhosis and hepatocellular carcinoma (HCC) [11]. Since 2018, no new studies have been conducted on the burden of HCV infection in Myanmar. The external validity and representativeness of the findings remain uncertain as most of the existing studies employed the convenience sampling methods [12]. Furthermore, the lack of detailed information on HCV infection characteristics within specific populations and geographic regions poses a significant challenge for the effective implementation of targeted control strategies [13]. Therefore, this study aims to provide the baseline epidemiological data on the disease burden of HCV infection in Myanmar and to determine the prevalence of HCV antibodies and HCV RNA and to assess the distribution of HCV genotypes across various populations from 1990 to 2023.

Materials and method

Study design and guidelines

This systematic review was conducted following the 2020 Preferred Reporting Items for Systematic Reviews and Meta-Analysis (PRISMA) guidelines [14] (S1 Appendix). Though we did not prospectively register our systematic review in the registry database such as International Prospective Register of Systematic Reviews (PROSPERO), International Platform of Registered Systematic Review and Meta-analysis Protocols (INPLASY) etc, there was no ongoing similar studies in those databases. Before the review, we searched databases such as PubMed, Web of Science, and Scopus for published studies on this topic and found none.

Data source and study selection

Searches in PubMed, Web of Science, and Scopus were conducted to identify the relevant records (S2 Table). Our review included studies conducted from January 1990 to May 2023 that reported on the prevalence of HCV antibodies, HCV RNA, and/or HCV genotypes, specifically including exact sample sizes and the number of positive cases. There were no restrictions on the type of study, and the subjects could be Myanmar citizens living inside or outside the country. However, clinical research or studies focusing on the prognosis of liver diseases in HCV-infected patients were excluded. Other factors such as

gender, age, work nature, and place of living did not affect the eligibility. Only reports in English were considered, and manual searches were conducted in The Myanmar Health Sciences Research Journal, Google Scholar, reference lists of eligible reports, and government reports. The last search was conducted on May 31, 2023, and duplicate records were removed using the Rayyan – Intelligent Systematic Review tool. Two independent investigators (ZP and KK) screened the records based on titles and abstracts, and relevant reports were retained for full-text review. In cases of disagreements, a third investigator (JT) made the final decision.

Data extraction

All relevant raw data, including authors' names, year of publication, study design, sampling method, study period, characteristics of study subjects (age, sex, location, comorbidities), sample size, number of participants positive for HCV antibodies/HCV RNA/HCV genotype, and type of biological assay, were extracted from each recruited study using Microsoft Excel 2010. The data were counter-checked by a second author. In this study, a "report" refers to any document mentioning HCV prevalence/genotype data, while a "study" pertains to the measurement of HCV antibodies/HCV RNA/HCV genotype in a specific population. Consequently, one report may describe multiple studies, and data were extracted separately for each study. In cases where study subjects were found to overlap in two or more different studies, only one publication was included.

Quality Assessment of the Included Studies

The Joanna Briggs Institute checklist for prevalence studies was used to assess the quality of the included studies [15] (S3 Table). The review process unified the minimum acceptable information for each variable. The minimum sample size for prevalence estimation was set at 312 subjects, calculated based on an assumed seroprevalence of 2.65% [10], a precision of 1.8%, and a confidence level of 95%. The sampling procedure of each study was examined in detail, and studies employing probabilistic sampling were considered representative of the target population. The validity of seroprevalence and genotype data relied on whether the diagnostics were based on standard procedures and methods.

Figure 1: PRISMA showing selection of articles for meta-analysis of HCV in Myanmar

Statistical Analysis

The study populations were categorized into four groups based on the characteristics of subjects: (1) low-risk group, (2) high-risk group, (3) liver disease patients, and (4) refugees/immigrants. The low-risk group included the general population, blood donors, and hepatitis B virus (HBV) vaccine non-responders. The high-risk group contained people living with human immunodeficiency virus (PLHIV), PWID, patients who received blood transfusions, and haemodialysis patients. The other two groups were simply categorized as liver disease patients and refugees/immigrants. The statistical analysis was conducted using the 'meta' and 'metafor' packages in R programming. The Freeman-Tukey double arcsine method was used to transform the data to accommodate small proportions. The meta-analysis was performed using the Dersimonian and Laird method, which utilizes a random-effects model, and the results were presented in a forest plot. For the low-risk group, which included three unpublished papers with a total sample size of 72,680, a sensitivity analysis was performed by excluding these unpublished papers. Confidence intervals (CI) for the proportions of individual studies were calculated using the Clopper-Pearson method. The Cochran Q test was employed to evaluate heterogeneity and then its degree was quantified by the I^2 index. Heterogeneity was considered significant if the p-value of the Cochran Q test was less than 0.05. The high, medium, or low level of heterogeneity was identified by the I^2 index thresholds of 75%, 50%, and 25% respectively [16]. A funnel plot of the transformed proportion against the sample size was generated to assess publication bias [17]. The symmetry of the plot was evaluated using the Egger test, with significance determined at a threshold of $p < 0.1$.

Inclusivity in global research

Additional information regarding the ethical, cultural, and scientific considerations specific to inclusivity in global research is included in the Supporting Information (S4 Checklist).

Results

Search results

A total of 126 articles were identified through the database search. Nine articles from the Myanmar Health Sciences Research Journal and government reports were manually added. After removing the duplication and

irrelevant articles, 85 articles were screened by their abstracts resulting in the exclusion of 35 articles based on the strict exclusion criteria. The remaining 50 articles were accessed for full text review and carefully evaluated for eligibility. Ultimately, 35 articles met the criteria and were included in the analysis to estimate the prevalence of HCV antibodies, HCV RNA and the distribution of HCV genotype in Myanmar (Fig 1). Some studies indicated the exact number of males and females or the place from which the samples were taken and also stated the age-specific number of samples and number of positive participants and corresponding prevalence, but most of the studies had lack of such information and it was quite difficult to divide the study subjects by their sex, age, and place of living. In terms of time series for included reports, 5, 9, and 13 reports were conducted during 1990-2000, 2001-2010, and 2011-2020 respectively but 8 reports did not describe the study year [18-24] (Table 1).

Figure 1: PRISMA showing a selection of articles for meta-analysis of HCV in Myanmar

Legend: This figure shows the selection process of the articles for the estimation of HCV prevalence in Myanmar. Each arrow indicates the step-by-step process for recruiting the articles and the detailed reason for the exclusion of articles were described with their respective exact number. This figure was built following the guideline for 2020 Preferred Reporting Items for Systematic Reviews and Meta-Analysis (PRISMA).

Table 1: Characteristics of included studies reporting the prevalence of HCV and genotype distribution in Myanmar between 1990 and 2023

First author, year of publication	Study design	Sampling method	Data collection period	Study subjects	Mean age or range (in years)	Sample size	Number of HCV-Ab positive	Number of HCV RNA-positive	Number of amplified samples	Assay for HCV-Ab/RNA testing
J Mellor, 1996[18]	C	Convenient	NR	Blood donors	NR	8	-	-	8	-
KP Kyi, 1998[25]	CS	Consecutive	1991	HCC	20-89	40	14	-	-	ELISA
M Khin, 2000†	CS	Convenience	NR	General population	1-74	569	16	-	-	NR
O Shigeru, 2000a^[26]	CS	NR	1996-1998	Thalassemia	7±3.5	15	7	-	-	ELISA
O Shigeru, 2000b^[26]	CS	NR	1996-1998	Blood receipts	20±9	18	10	-	-	ELISA
O Shigeru, 2000c^[26]	CS	NR	1996-1998	Liver diseases*	3-60	74	32	-	-	ELISA
O Shigeru, 2000d^[26]	CS	NR	1996-1998	HCC	47±12	28	8	-	-	ELISA
N Win, 2000[27]	CS	NR	1996	HCC	23-70	16	7	-	-	ELISA
N Kazuhiko, 2001a^[28]	CS	Convenience	1998-2000	Blood donors	7-80	213	-	4	-	PCR
N Kazuhiko, 2001bd^[28]	CS	Convenience	1998-2000	Liver diseases*	7-80	165	-	42	24	PCR
N Kazuhiko, 2001c^[28]	CS	Convenience	1998-2000	HCC	7-80	25	-	6	-	PCR

T Kazuhisa, 2002a^[20]	CS	NR	NR	Liver diseases*	NR	29	14	-	-	IRMA
T Kazuhisa, 2002b^[20]	CS	NR	NR	HCC	NR	20	4	-	-	IRMA
KP Kyi, 2002a^[19]	CS	Consecutive	NR	General population	NR	362	9	-	-	Immunoblot

First author, year of publication	Study design	Sampling method	Data collection period	Study subjects	Mean age or range (in years)	Sample size	Number of HCV-Ab positive	Number of HCV RNA-positive	Number of amplified samples	Assay for HCV-Ab/RNA testing
KP Kyi, 2002b^[19]	CS	Convenience	NR	Liver diseases*	NR	379	95	-	-	Immunoblot
M Khin, 2003†	CS	Consecutive	2000-2004	Blood donors	NR	154,161	4,008	-	-	NR
T Shinji, 2004[20]	RC	Convenient	2000	Blood donors	18-51	201	-	-	110	-
AA Lwin, 2007ab^[21]	CS	Convenience	NR	General population	1-75	1,333	154	-	145	PA
A Denburg, 2007[29]	C	Consecutive	2006	Refugees	NR	68	0	-	-	NR
A Thu, 2008[9]	CS	Convenience	2007-2008	PWID	29.49±6.3	298	236	-	-	Rapid test
NJ Chaves, 2009[30]	RC	Consecutive	2004-2008	Refugees	16-86	145	-	4	-	PCR
M Khin, 2010[31]	CS	Consecutive	2005-2007	Blood donors	18-60	65,237	621	-	-	Rapid test
A Srunthron, 2010abc^[32]	CS	Consecutive	2007-2009	Immigrants	15-60	1,594	27	15	15	ELISA (HCV-Ab)

										PCR (HCV RNA)
MM Htun, 2010ab^[22]	CS	Consecutive	NR	HBV vaccine non-responders	NR	23	0	1	-	PA (HCV-Ab) PCR (HCV RNA)
YH Zhou, 2011[33]	CS	Convenience	2005-2007	PWID	31.8±9.8	318	153	-	-	ELISA
GA Paxton, 2012[34]	CS	Consecutive	2007-2009	Refugees	0.5-87	519	10	-	-	NR
TM Hayden, 2014ab^[35]	CS	Convenience	2002-2007	Refugees	15-95	1,076	-	4	4	PCR
MOH Myanmar, 2015†	CS	Consecutive	2015	Blood donors	15-80	379,088	1,895	-	-	Rapid test
C Naing, 2015[23]	RC	Convenient	NR	Chronic hepatitis C	47.1±11.6	362	-	-	350	-

First author, year of publication	Study design	Sampling method	Data collection period	Study subjects	Mean age or range (in years)	Sample size	Number of HCV-Ab positive	Number of HCV RNA-positive	Number of amplified samples	Assay for HCV-Ab/RNA testing
AA Lwin, 2017[10]	CS	Random	2015	General population	48 ± 14.6	5,547	147	-	-	ICT
AA Lwin, 2018[36]	CS	NR	2016	Haemodialysis patients	20-40	111	17	-	-	ICT
NS Aye, 2018[37]	RC	NR	2015-2017	PWID	24-34	531	376	-	-	Rapid test
CC Ngo, 2018[38]	RC	Consecutive	2013-2014	Refugees	30-70	51	5	-	-	NR

HS Juon, 2019[39]	CS	Convenience	2009-2015	Immigrants	37.3 ± 13	927	45	-	-	NR
M Ye, 2019ab^[40]	CS	NR	2014	PWID	16-74	100	40	-	25	ELISA
M Chen, 2019[41]	CS	Convenient	2014	PWID	22-33	7	-	-	7	-
Myanmar IBBS, 2019[42]	CS	RDS	2017-2018	PWID	34.62 ± 9.25	6,061	3,394	-	-	Rapid test
KT Nyunt, 2019[24]	CS	NR	NR	General population	35.3	178	1	-	-	ICT
TS Win, 2020[43]	CS	NR	2016	PLHIV	35 (30-42)	15	150	-	-	ELISA
NTT Kyaw, 2022[44]	C	Consecutive	2005-2016	PLHIV	1-65	27,722	2,265	-	-	OraQuick
K Urban, 2023[45]	CS	Consecutive	2010-2017	Refugees	NR	18,059	511	-	-	NR
TM Swe, 2023abc^[8]	C	Consecutive	2016-2017	PLHIV	15-80	21,777	1,417	1,143	640	OraQuick (HCV-Ab)<br>PCR (HCV RNA)

HCV-Ab, hepatitis C virus antibodies; *HCV RNA*; hepatitis C virus ribonucleic acid; *MOH*, Ministry of Health; *IBBS*, integrated biological and behavioral survey; *CS*, cross-sectional; *C*, cohort; *RC*, retrospective cohort; *NR*, not reported; *RDS*, respondent driven sampling; *HBV*, hepatitis B virus; *HCV*, hepatitis C virus; *HCC*, hepatocellular carcinoma; *PWID*, people who inject drugs; *PLHIV*, people living with human immunodeficiency virus; *ELISA*, enzyme-linked immunosorbent assay; *PCR*, polymerase chain reaction; *ICT*, immunochromatography; *IRMA*, immunoradiometric assay; *CLIA*, chemiluminescence immunoassay; *PA*, particle agglutination.

*Patients with liver diseases other than hepatocellular carcinoma; †Unpublished observations, ^Studies issued from the same report.

Study characteristics

Out of a total of 135 reports screened for eligibility, 35 reports comprising 51 studies were included in which 33 studies provided data on HCV seroprevalence in 685,403 individuals, 8 studies reported HCV RNA prevalence in 25,018 individuals, and 10 studies examined HCV genotypes in 1,857 individuals. The studies included in the analysis were conducted from 1991 to 2018 (Table 1). In HCV antibodies and RNA prevalence studies, the cross-sectional design was primarily used in 34 out of 41 studies (82.9%), while only one study [10] (2.4%) employed a probabilistic sampling method. In terms of sample size, 53.7% (22/41) of the studies included adequate sample sizes (>312). HCV antibodies and HCV RNA were determined through biological assays which were reported in 83.0% (34/41) of the studies (S2 Table).

Regarding the estimation of HCV seroprevalence, 28 out of 33 studies conducted cross-sectional studies, two studies utilized retrospective cohort designs in which the prevalence was estimated from laboratory records, and the remaining three studies employed cohort studies where HCV seroprevalence was determined as part of the overall study (Table 1). For the HCV RNA prevalence study, six out of eight studies performed cross-sectional studies, one study adopted a retrospective cohort, and one study employed a cohort study (Table 1). Out of the ten studies conducted for the HCV genotype study, five utilized cross-sectional study designs, three conducted a retrospective cohort approach, and two employed a cohort study methodology (Table 1). All included reports covered a study period ranging from 1991 to 2018, with the majority of studies conducted after 2010.

In terms of participant characteristics in the HCV seroprevalence estimate, nine studies focused on low-risk groups, including the general population, blood donors, and individuals who did not respond to HBV vaccines, encompassing a total of 60,648 participants. Eleven studies targeted high-risk groups, such as PWID, PLHIV, patients who received blood transfusions, and individuals undergoing hemodialysis, with a total of 57,101 participants. Seven studies focused on liver disease patients, specifically hepatocellular carcinoma (HCC), liver cirrhosis, and acute/chronic hepatitis, including 586 patients. Finally, six studies targeted refugees/immigrants, comprising a total of 21,218 individuals (Table 1).

In the HCV RNA prevalence study, two studies focused on low-risk groups, including blood donors and individuals who did not respond to HBV vaccines, with a total of 236 subjects. One study targeted a high-risk group of PLHIV, involving 21,777 individuals. Two studies focused on liver disease patients, including one study on HCC and one study on other liver diseases, encompassing 190 patients. Additionally, three studies specifically

targeted refugees/immigrants, comprising a total of 2,815 individuals (Table 1).

In the study on HCV genotypes, three studies examined low-risk groups, which included the general population and blood donors, with a combined sample size of 363 subjects. Another three studies focused on high-risk groups such as PWID and PLHIV, involving 911 individuals. Two studies concentrated on patients with liver disease, encompassing a total of 552 patients. Furthermore, two studies specifically targeted refugees, with a total of 19 participants (Table 1).

Seroprevalence of HCV antibodies

Seroprevalence in the low-risk group

Low-risk populations were addressed in 9 studies and the total number of subjects screened was 606,498 of which 6,851 subjects were positive. Five studies focused on the general population, three on blood donors, and one on HBV vaccine non-responders. The seroprevalence ranged between 0 and 11.55% and the pooled seroprevalence of HCV was estimated at 2.18% (95%CI: 1.09-3.58). However, significant heterogeneity was found in this group (I^2=99.8%, p=0) (Fig 2). A sensitivity analysis indicated that the prevalence of HCV antibodies increased to 2.41% (95% CI: 0.51%-5.4%) after excluding 3 unpublished reports (n=72,680). However, this difference was not statistically significant (the result not shown).

Figure 2: Forest plot of the pooled HCV seroprevalence in Myanmar, using random effect

Legend: This figure shows the funnel plot of 33 studies and the pooled HCV seroprevalence in sub-groups: low-risk group, high-risk group, liver disease patients, and refugees/immigrants. The diamond block represents for pooled prevalence and the rectangular block represents prevalence for each study.

Seroprevalence in the high-risk group

Individuals in the high-risk group were investigated in eleven studies: five on PWID, three on PLHIV, two on patients who received blood transfusion, and one on hemodialysis patients. A total of 57,101 high-risk individuals

were studied, and 7,930 were HCV antibodies positive. Seroprevalence varied from 6.51% to 79.19%, and the pooled seroprevalence was 37.07% (95%CI: 22.85-52.49). Heterogeneity was also significant in this group (I^2=99.89%, p=0) (Fig 2).

Seroprevalence in the liver disease patients

Liver disease patients were investigated in seven studies: four on hepatocellular carcinoma patients and three on other liver disease patients. Out of a total of 586 patients, 174 were HCV antibodies positive. Seroprevalence varied from 20.00% to 48.28%, and the pooled seroprevalence was 33.84% (95% CI: 25.29-42.93). Heterogeneity was also significant in this group (I^2=65.13%, p=0.0086) (Fig 2).

Seroprevalence in the immigrants/refugees

Myanmar citizen immigrants/refugees who are living outside the country were investigated in six studies. Among a total of 21,218 individuals, 598 were HCV antibodies positive. Seroprevalence varied from 0 to 9.80% and the pooled seroprevalence was 2.52% (95%CI: 1.52-3.73). Heterogeneity was also significant in this group (I^2=83.24%, p=<0.0001) (Fig 2).

Seroprevalence by study subjects

The highest HCV antibodies positive rate was found in PWID (58.06%, 95% CI: 56.94–59.18), individuals receiving blood transfusion (51.53%, 95% CI: 34.55–68.52), and liver disease patients (28.80%, 95% CI: 25.19-32.42). The seroprevalence of HCV among the general population was 2.82% (95% CI: 2.46–3.18, I^2 = 96.57%, $p < 0.0001$), and that among blood donors was 0.68% (95% CI: 0.66-0.70, I^2=99.92%, p=<0.00001) (Table 2).

253

Table 2: HCV seroprevalence in sub-groups

Categories	Number of studies	Total population	Pooled seroprevalence	95% CI	I^2 index (%)	Cochran Q test p-value
Study subjects						
General population	5	7,989	2.82	2.46-3.18	96.57	<0.00001
Blood donors	3	598,486	0.68	0.66-0.70	99.92	<0.00001
HBV vaccine non-responders	1	23	0.00	0.00-0.00	NA	NA
PWID	5	7,308	58.06	56.94-59.18	97.49	<0.00001
PLHIV	3	49,649	7.36	7.13-7.59	96.12	<0.00001
Patients who received blood transfusion	2	33	51.53	34.55-68.52	NA	NA
Haemodialysis patients	1	111	15.32	8.62-22.02	NA	NA
Liver disease patients*	7	586	28.80	25.19-32.42	63.87	0.01
Refugees/immigrants	6	21,218	2.72	2.50-2.94	79.70	0.0002
Data collection period†						
2000-2005	1	154,161	2.60	2.52-2.70	NA	NA
2006-2010	1	65,237	0.95	0.90-1.03	NA	NA

2011-2015	2	384,635	0.51	0.48-0.53	99.99	<0.00001

HBV, hepatitis B virus; *PWID*, people who inject drugs; *PLHIV*, people living with human immunodeficiency virus; *NA*, not applicable.

* Patients with liver diseases including hepatocellular carcinoma; [†] Analyses performed considering only low-risk groups (blood donors and general population).

Seroprevalence by the study period of data collection

Only low-risk groups were considered for HCV seroprevalence estimate by the study period to see the pure trend of its prevalence. One unpublished study was conducted between 2000-2005, resulting in a seroprevalence of 2.60% (95%CI: 2.52-2.70). The seroprevalence decreased to 0.95% (95%CI: 0.90-1.03, one study[31]) during 2006-2010 and to 0.51% (95%CI: 0.48-0.53, I^2=99.99%, p=<0.00001) during 2011-2015 (two studies including one unpublished study[10]) (Table 2). This decreasing trend was significant (p for difference=0.03).

HCV RNA prevalence

Eight studies[8, 22, 28, 30, 32, 35] measured HCV RNA, and the positive rate ranged from 0.37% in refugees to 25.45% in liver disease patients. The pooled prevalence of HCV RNA was estimated at 1.40% (95%CI: 0.08-3.71, I^2=0%, p=0.3390) in low-risk group, 5.25% (95%CI: 4.96-5.55) in high-risk group, 24.96% (95%CI: 18.91-31.51, I^2=0%, p=0.0115) in patients with liver diseases and 0.84% (95%CI: 0.19-1.84, I^2=73.65%, p=0.0225) in refugees/immigrants (Fig 3).

Figure 3: Forest plot of the pooled HCV RNA prevalence in Myanmar, using random effect

Legend: This figure shows the funnel plot of 7 studies and the pooled HCV RNA prevalence in sub-groups: low-risk group, liver disease patients, and refugees/immigrants. The diamond block represents for pooled prevalence and the rectangular block represents prevalence for each study.

HCV genotypes

The genotyping of the HCV was performed in 10 studies[8, 18, 21, 23, 28, 32, 35, 40, 41, 46]. Overall, HCV genotype 3 was most predominant (48%) followed by genotypes 1 (28%), 6 (23%), 2 (1%), and 4 (0.27%). By sub-group analysis, genotype 3 is predominant in all sub-groups of low-risk, high-risk, liver disease patients and refugees/immigrants (43%, 48%, 52%, and 53% respectively). The HCV genotype distribution pattern was similar between low-risk groups and refugees/immigrants: genotype 3 (43% vs. 53%) followed by 6 (36% vs. 37%) and 1 (21% vs. 11%). The HCV genotype distribution was the similar in high-risk groups and liver disease patients:

genotype 3 (48% vs 52%) followed by 1 (31% vs. 28%) and 6 (21% vs. 18%). HCV genotype 2 was exclusively found in low-risk and liver disease patients (1% and 2% respectively) and only one case of genotype 4 was reported in a high-risk group (PLHIV) (Fig 4). The analysis of HCV genotype distribution by region showed that genotype 6 is predominant in the northern part of Myanmar whilst genotype 3 is predominant in the west and southern parts of Myanmar (Fig 5).

Figure 4: Distribution of HCV genotypes in Myanmar

Legend: This figure shows the HCV genotype distribution overall and by group of population in Myanmar. Each color represents each genotype: light blue for Genotype 1, orange for Genotype 2, dark blue for Genotype 3, and light green for Genotype 6.

Figure 5: HCV genotypes distribution by study place in Myanmar (N=1,294)

Legend: This figure shows the analysis of HCV genotype distribution by region in Myanmar. Each pie chart represents each study place. Each color inside the pie chart represents each genotype: light blue for Genotype 1, orange for Genotype 2, dark blue for Genotype 3, and light green for Genotype 6.

Publication bias

Using the graphical assessment of the funnel plot, the publication bias was found in HCV seroprevalence studies, but there was no publication bias in HCV RNA prevalence studies. In the Egger tests for funnel plot asymmetry, the p-values were 0.0001 and 0.8392 respectively (Fig 6a and 6b).

Figure 6: Funnel plot of HCV prevalence a) HCV antibodies and b) HCV RNA

Figures 6a and 6b show the forest plot for the assessment of publication bias among the recruited studies for estimating pooled HCV antibodies and HCV RNA prevalence. The dotted line represents the resultant pooled

prevalence, and each black circle represents the prevalence of each study. The prevalence was plotted against the sample size.

Discussion

This systematic review revealed Myanmar has the intermediate endemicity of HCV seroprevalence among low-risk groups including the general population. The analysis of HCV seroprevalence by sub-groups offered a more comprehensive understanding of the disease burden within each group in Myanmar. The disease spectrum differs by the characteristic of each group, and it is encountered to consider adopting effective countermeasures against HCV in Myanmar.

This meta-analysis revealed publication bias in HCV seroprevalence studies but not in HCV RNA prevalence studies. The presence of publication bias indicates a likelihood of overestimating seroprevalence rates due to the selective publication of studies with higher rates. Therefore, the pooled prevalence was not determined as a whole, and the participants were categorized into four groups: low-risk groups, high-risk groups, liver disease patients, and immigrants/refugees computed the pooled prevalence in each group using a random effect. This meta-analysis included the Burmese immigrants or refugees residing in other countries and it is believed that this group of the population reflects the disease burden of HCV among the general population in Myanmar.

HCV seroprevalence in low-risk groups and immigrants/refugees were 2.18% and 2.52% respectively. Despite the sensitivity analysis showing a slight increase in HCV antibody prevalence after removing the unpublished reports in low-risk groups, the change was not statistically significant. However, caution should be exercised when interpreting the pooled prevalence estimates. A significantly high level of heterogeneity was noticed in both groups (I^2: 99.8% vs. 83.24%). Considering the prevalence of 2.18% in the general population, 1.18 million individuals were exposed to HCV infection across the country as of 2023 Myanmar's population data [47]. In Myanmar, a nationwide study on the prevalence of HBV and HCV was conducted in 18 study sites in 2015 and reported that the prevalence of HBV and HCV was 6.5% and 2.7% respectively [10]. The pooled HCV seroprevalence among low-risk groups and immigrants/refugees in this meta-analysis is consistent with a 2015 nationwide study. The studies conducted from 2000 through 2017 among the abovementioned population groups likely do not reflect the current epidemiological situation of HCV among the general population in Myanmar.

The pooled HCV seroprevalence of 37.07% was found in high-risk groups which comprised of frequent blood recipients (Thalassemia etc.), hemodialysis patients, PWID, and PLHIV, all of whom were highly prone to HCV infection. A significant high level of heterogeneity was observed in this group with reported I^2 at 99.8%. The reported studies were conducted from 1996 through 2018. The high HCV seroprevalence rates among PWID, ranging from 40% to 79%, highlight the critical importance of implementing targeted interventions although harm reduction strategies that have been introduced since 2005-2006 in Myanmar. Moreover, the HIV/HCV coinfection rate ranged from 5% to 22.8% and the risk was high in the PWID population of Myanmar (Odds Ratio: 20.1, 95% CI: 13.7 to 29.5) [48, 49] which highlights the interplay between these two infections and the need for integrated prevention and treatment approaches in PWID.

The pooled HCV seroprevalence among liver disease patients was calculated to be 33.84% with a reported medium heterogeneity of 65.13%. All the studies on liver disease patients were conducted between 1991 and 1998 and there was no report or studies after 2002. Most of the studies used convenient sampling methods for participant recruitment and the under or overestimation of HCV seroprevalence among this group cannot be ruled out. After two decades, the attribution of HBV and HCV etiology to liver disease including HCC might be changed and it needs to be evaluated again.

The decreasing trend of HCV seroprevalence among the low-risk group was found by a period of data collection. It can be explained by the installation of blood donor screening in 2000, the promotion of infection control including disposable syringe usage in health care facilities in the early 2000 and raising the awareness of viral hepatitis in 2011 with local and international non-governmental organizations' involvement in viral hepatitis screening and health education.

HCV RNA positive rate was supposed to be low in low-risk and immigrants/refugee groups (1.4% vs 0.84%) assuming that approximately 50% of them were naturally resolved and the number of viremic cases was estimated at 0.7 million individuals in Myanmar. The high HCV RNA-positive rate was found in liver disease patients (24.96%) but its attribution to hepatocellular carcinoma is unknown.

There were no incidence studies on HCV infection in Myanmar and only a few of the included prevalence studies were conducted mostly in an urban area with representative samples of the target population and there were no studies or reports on HCV disease burden after the 2015 nationwide study. Furthermore, the coronavirus disease 2019 (COVID-19) pandemic together with the unstable political situation in 2021 delayed or interrupted the healthcare system in Myanmar which is threatening the infectious diseases control, countermeasures, and the road

to elimination of viral hepatitis in Myanmar. A well-structured basic epidemiological study to examine the disease burden of HCV in Myanmar is recommended.

In Myanmar, the predominant HCV genotype was 3 (48%) followed by 1 (28%) and 6 (23%) overall. This information is significant in guiding HCV treatment strategies, as genotypes can impact disease progress, treatment response, and duration. For instance, genotype 3 is associated with a higher risk of developing severe liver injuries, such as cirrhosis and hepatocellular carcinoma [50]. The obvious geographical difference in genotype distribution was found across the country. In the northern region of Myanmar, which shares a border with China's Yunnan province, a high prevalence of HCV genotype 6 was observed in the PWID population. Likewise, PWID in Yunnan province, HCV genotype 6 was found to be the predominant genotype. This finding suggests that there may be transmission of HCV among PWID populations occurring through the border regions between Myanmar and Yunnan province [51]. However, HCV genotype 3 was predominant in the southern part of Myanmar, and the southern east of Myanmar is bordered by Thailand where genotype 3 is reportedly predominant [52]. HCV genotype 3 was also predominant in the western part of Myanmar, which shares borders with India and Bangladesh. These neighboring countries also have a high prevalence of genotype 3 [53, 54]. Understanding the genotype distribution and its geographical difference revealed the potential route of transmission from which effective prevention and control strategies should be developed. The genotypic diversity observed in Myanmar serves as a valuable contribution to the overall understanding of HCV genotypes in the Southeast Asia region.

This study has several limitations. The significant heterogeneity among the included studies may introduce variations in the estimated prevalence rates. This heterogeneity can be attributed to differences in sampling methods, individual characteristics of the study population, involvement of small sample sizes in some studies, and sampling biases in each recruited study so that the over or underestimation of the prevalence cannot be excluded. Most of the included studies did not provide the study setting clearly so that the urban and rural areas cannot be categorized, but most studies were assumed to be conducted in urban settings so the study in rural settings is limited in Myanmar and the actual disease burden among rural population is unknown. Moreover, the study on HCV among not only key populations such as men who have sex with men (MSM), female sex workers (FSW), and people in prisons but also the health care professionals (HCP) and pregnant women are still unknown.

In conclusion, this systematic review and meta-analysis indicated that Myanmar has intermediate endemicity of HCV seroprevalence in the low-risk group with a decreasing trend over the past 20 years due to measures such as blood donor screening, infection control practices, and increased awareness through education programs. However,

the findings highlight the need for further epidemiological studies to understand the actual disease burden and implement effective countermeasures, including screening, linkage to care, and accessibility to treatment, to achieve the WHO's goal of HCV elimination by 2030.

List of abbreviations

HCV: Hepatitis C virus; DAA: Direct-acting antivirus; RNA: Ribonucleic acid; WHO: World Health Organization; PRISMA: Preferred Reporting Items for Systematic Reviews and Meta-Analysis; PROSPERO: International Prospective Register of Systematic Reviews; INPLASY: International Platform of Registered Systematic Review and Meta-analysis Protocols; CI: Confidence Interval; MMA: Myanmar Medical Association; MLF: Myanmar Liver Foundation; MSF: Medecins Sans Frontieres; NHCP: National Hepatitis Control Program; SVR: Sustained virological response; PLHIV: People living with human immunodeficiency virus; PWID: People who inject drugs; HBV: Hepatitis B Virus HCC: Hepatocellular carcinoma; HIV: Human immunodeficiency virus; MSM: Men having sex with men; FSW: Female sex workers; HCP: Health care professional.

Declarations

Ethical approval and consent to participate

Not applicable.

Consent for publication

Not applicable.

Competing interests

The authors declare no conflict of interest.

Role of funding sources

This work was supported by Japan Society for the Promotion of Science (JSPS) Core-to-Core Program (Grant number: JPJSCCB20240009). The funders had no role in study design, data collection and analysis, decision to publish, or preparation of the manuscript.

Availability of data

The dataset used and analysed in this study is available from the corresponding author upon reasonable request.

Acknowledgment

We would send heartfelt gratitude to all the authors of the original papers which were used in this systematic review and meta-analysis. We would like to thank the personnel of the Department of Epidemiology, Infectious Disease Control, and Prevention, Graduate School of Biomedical and Health Sciences, Hiroshima University, for their valuable support.

Author contributions

ZP, KK, and JT conceptualized the study. ZP, KK, and SO contributed to developing the study design and data acquisition. ZP, KK, SO, TA, and JT analysed and interpreted the data. ZP, KK, and SO drafted the manuscript. AS, UM, GA, CC, TA, and JT contributed to the intellectual content of the manuscript. All authors read and approved the final manuscript.

References

1. HCV Fact Sheet. World Health Organization (WHO). 2024 April 9. [Cited 2024 June 7]. Available from: https://www.who.int/news-room/fact-sheets/detail/hepatitis-c.

2. Global Hepatitis Report. World Health Organization (WHO); 2017.

3. Polaris Public Access, Regional Dashboard. [Cited 2023 June 28]. Available from https://cdafound.org/polaris-regions-compare/.

4. Yee WL, Bowring A, Draper B, O'Keefe D, Htay H, Myint KT, et al. Patients' access to and acceptance of community-based hepatitis C testing and treatment in Myanmar: A mixed-method study. PLOS Glob Public Health. 2023;3(6):e0000902. Epub 2023/06/16. doi: 10.1371/journal.pgph.0000902. PubMed PMID: 37327249; PubMed Central PMCID: PMCPMC10275420.

5. Myanmar national simplified treatment guidelines for hepatitis C infection. Ministry of Health and Sports Myanmar. 2nd edn, 2019. [Cited 2024 June 7]. Available from: https://mohs.gov.mm/page/3212.

6. Draper BL, Pedrana A, Howell J, Yee WL, Htay H, Aung KS, et al. Decentralized, Community-Based Hepatitis C Point-of-Care Testing and Direct-Acting Antiviral Treatment for People Who Inject Drugs and the General Population in Myanmar: Protocol for a Feasibility Study. JMIR Res Protoc. 2020;9(7):e16863. Epub 2020/07/17. doi: 10.2196/16863. PubMed PMID: 32673260; PubMed Central PMCID: PMCPMC7388045.

7. Draper BL, Yee WL, Shilton S, Bowring A, Htay H, Nwe N, et al. Feasibility of decentralised, task-shifted hepatitis C testing and treatment services in urban Myanmar: implications for scale-up. BMJ Open. 2022;12(5):e059639. Epub 2022/05/04. doi: 10.1136/bmjopen-2021-059639. PubMed PMID: 35504640; PubMed Central PMCID: PMCPMC9066562.

8. Swe TM, Johnson DC, Mar HT, Thit P, Homan T, Chu CM, et al. Epidemiological characteristics and real-world treatment outcomes of hepatitis C among HIV/HCV co-infected patients in Myanmar: A prospective cohort study. Health Sci Rep. 2023;6(2):e1119. Epub 2023/02/24. doi: 10.1002/hsr2.1119. PubMed PMID: 36819986; PubMed Central PMCID: PMCPMC9938359.

9. Thu A, Thaw A, Oo KM, Lwin AA, Lwin O, Myint AZ, et al. Prevalence and associated risks of hepatitis B and C infections among Injecting Drug Users (IDU) attending the registered Drug Treatment Centers in Yangon, Mandalay, Myitkyina, Bamaw and Moekaung in 2007. Myanmar Health Sciences Research Journal. 2008; 20(3): 159-164.

10. Lwin AA, Aye KS, Htun MM, Kyaw YY, Zaw KK, Aung TT, et al. Sero-prevalence of Hepatitis B and C Viral Infections in Myanmar: National and Regional Survey in 2015. Myanmar Health Sciences Research Journal. 2017; 29(3): 167-175.

11. Spearman CW, Dusheiko GM, Hellard M, Sonderup M. Hepatitis C. Lancet. 2019;394(10207):1451-66. Epub 2019/10/22. doi: 10.1016/s0140-6736(19)32320-7. PubMed PMID: 31631857.

12. Andrade C. The Inconvenient Truth About Convenience and Purposive Samples. Indian J Psychol Med. 2021;43(1):86-8. Epub 2021/08/06. doi: 10.1177/0253717620977000. PubMed PMID: 34349313; PubMed Central PMCID: PMCPMC8295573.

13. Petruzziello A, Marigliano S, Loquercio G, Cacciapuoti C. Hepatitis C virus (HCV) genotypes distribution: an epidemiological up-date in Europe. Infect Agent Cancer. 2016;11:53. Epub 2016/10/19. doi: 10.1186/s13027-016-0099-0. PubMed PMID: 27752280; PubMed Central PMCID: PMCPMC5062817.

14. Page MJ, McKenzie JE, Bossuyt PM, Boutron I, Hoffmann TC, Mulrow CD, et al. The PRISMA 2020 statement: an updated guideline for reporting systematic reviews. Bmj. 2021;372:n71. Epub 2021/03/31. doi: 10.1136/bmj.n71. PubMed PMID: 33782057; PubMed Central PMCID: PMCPMC8005924.

15. Munn Z, Moola S, Lisy K, Riitano D, Tufanaru C. Methodological guidance for systematic reviews of observational epidemiological studies reporting prevalence and cumulative incidence data. Int J Evid Based Healthc. 2015;13(3):147-53. Epub 2015/09/01. doi: 10.1097/xeb.0000000000000054. PubMed PMID: 26317388.

16. Higgins JP, Thompson SG, Deeks JJ, Altman DG. Measuring inconsistency in meta-analyses. Bmj. 2003;327(7414):557-60. Epub 2003/09/06. doi: 10.1136/bmj.327.7414.557. PubMed PMID: 12958120; PubMed Central PMCID: PMCPMC192859.

17. Hunter JP, Saratzis A, Sutton AJ, Boucher RH, Sayers RD, Bown MJ. In meta-analyses of proportion studies, funnel plots were found to be an inaccurate method of assessing publication bias. J Clin Epidemiol. 2014;67(8):897-903. Epub 2014/05/06. doi: 10.1016/j.jclinepi.2014.03.003. PubMed PMID: 24794697.

18. Mellor J, Walsh EA, Prescott LE, Jarvis LM, Davidson F, Yap PL, et al. Survey of type 6 group variants of hepatitis C virus in Southeast Asia by using a core-based genotyping assay. J Clin Microbiol. 1996;34(2):417-23. Epub 1996/02/01. doi: 10.1128/jcm.34.2.417-423.1996. PubMed PMID: 8789027; PubMed Central PMCID: PMCPMC228809.

19. Kyi KP, Aye M, Oo KM, Htun MM, Oo SS, Lwin KO, et al. Prevalence of Hepatitis C in Healthy Population and Patients with Liver Ailments in Myanmar. Regional Health Forum. 2002; 6(1): 1-6.

20. Taketa K, Okada S, Win N, Hlaing NK, Wind KM. Evaluation of tumor markers for the detection of hepatocellular carcinoma in Yangon General Hospital, Myanmar. Acta Med Okayama. 2002;56(6):317-20. Epub 2003/04/11. doi: 10.18926/amo/31689. PubMed PMID: 12685861.

21. Lwin AA, Shinji T, Khin M, Win N, Obika M, Okada S, et al. Hepatitis C virus genotype distribution in Myanmar: Predominance of genotype 6 and existence of new genotype 6 subtype. HEPATOLOGY RESEARCH. 2007;37(5):337-45. doi: 10.1111/j.1872-034X.2007.00053.x. PubMed PMID: WOS:000245693200005.

22. Htun MM, Kyi KP, Oo KM, Khin M, Tun WM, Oo SS, et al. Presence of hepatitis C virus RNA by nested polymerase chain reaction (RT-PCR) in plasma-derived hepatitis B vaccine non-responders. Myanmar Health Sciences Research Journal. 2010; 22(2): 101-104.

23. Naing C, Sitt T, Aung AT, Aung K. Sustained Virologic Response to a Dual Peginterferon alfa-2a and Ribavirin in Treating Chronic hepatitis C Infection: A Retrospective Cohort Study. Medicine (Baltimore). 2015;94(30):e1234. Epub 2015/07/30. doi: 10.1097/md.0000000000001234. PubMed PMID: 26222859; PubMed Central PMCID: PMCPMC4554122.

24. Nyunt KT, Htoo KPP, Hnin MT, Kyaw PM, Mon EK, Thu ISL, et al. Hepatitis B and Hepatitis C infection among teaching staff and non-teaching from University of Medical Technology, Mandalay. Myanmar Medical Journal. 2019; 61(4): 35-43.

25. Kyi KP, Win KM, Aye M, Htwe YY, Oo KM, Aung T, et al. Prevalence of hepatitis B and C infections in hepatocellular carcinoma cases in Myanmar. Myanmar Health Sciences Research Journal. 1998; 10(1): 1-5.

26. Okada S, Taketa K, Ishikawa T, Koji T, Swe T, Win N, et al. High prevalence of hepatitis C in patients with thalassemia and patients with liver diseases in Myanmar (Burma). ACTA MEDICA OKAYAMA. 2000;54(3):137-8. PubMed PMID: WOS:000087965700007.

27. Win N, Okada S, Win KM, Taketa K, Akiyama T, Aye KS, et al. Hemoglobinopathies, Iron Overload and Chronic Viral Hepatitis in Patients with Hepatocellular Carcinoma in Myanmar. Asian Pac J Cancer Prev. 2000;1(2):127-30. Epub 2003/04/30. PubMed PMID: 12718679.

28. Nakai K, Win KM, Oo SS, Arakawa Y, Abe K. Molecular characteristic-based epidemiology of hepatitis B, C, and E viruses and GB virus C/hepatitis G virus in Myanmar. JOURNAL OF CLINICAL MICROBIOLOGY. 2001;39(4):1536-9. doi: 10.1128/JCM.39.4.1536-1539.2001. PubMed PMID: WOS:000167946500055.

29. Denburg A, Rashid M, Brophy J, Curtis T, Malloy P, Audley J, et al. Initial health screening results for Karen refugees: a retrospective review. Can Commun Dis Rep. 2007;33(13):16-22. Epub 2007/12/29. PubMed PMID: 18161207.

30. Chaves NJ, Gibney KB, Leder K, O'Brien DP, Marshall C, Biggs BA. Screening practices for infectious diseases among Burmese refugees in Australia. Emerg Infect Dis. 2009;15(11):1769-72. Epub 2009/11/07. doi: 10.3201/eid1511.090777. PubMed PMID: 19891864; PubMed Central PMCID: PMCPMC2857247.

31. Khin M, Oo SS, Oo KM, Shimono K, Koide N, Okada S. Prevalence and Factors Associated with Hepatitis C Virus Infection among Myanmar Blood Donors. ACTA MEDICA OKAYAMA. 2010;64(5):317-21. PubMed PMID: WOS:000283563300007.

32. Akkarathamrongsin S, Praianantathavorn K, Hacharoen N, Theamboonlers A, Tangkijvanich P, Poovorawan Y. Seroprevalence and Genotype of Hepatitis C Virus among Immigrant Workers from Cambodia and Myanmar in Thailand. INTERVIROLOGY. 2011;54(1):10-6. doi: 10.1159/000318884. PubMed PMID: WOS:000282170300002.

33. Zhou YH, Liu FL, Yao ZH, Duo L, Li H, Sun Y, et al. Comparison of HIV-, HBV-, HCV- and Co-Infection Prevalence between Chinese and Burmese Intravenous Drug Users of the China-Myanmar Border Region. PLOS ONE. 2011;6(1). doi: 10.1371/journal.pone.0016349. PubMed PMID: WOS:000286522300038.

34. Paxton GA, Sangster KJ, Maxwell EL, McBride CR, Drewe RH. Post-arrival health screening in Karen refugees in Australia. PLoS One. 2012;7(5):e38194. Epub 2012/06/14. doi: 10.1371/journal.pone.0038194. PubMed PMID: 22693599; PubMed Central PMCID: PMCPMC3364970.

35. Mixson-Hayden T, Lee D, Ganova-Raeva L, Drobeniuc J, Stauffer WM, Teshale E, et al. Hepatitis B virus and hepatitis C virus infections in United States-bound refugees from Asia and Africa. Am J Trop Med Hyg. 2014;90(6):1014-20. Epub 2014/04/16. doi: 10.4269/ajtmh.14-0068. PubMed PMID: 24732462; PubMed Central PMCID: PMCPMC4047722.

36. Lwin AA, Htwe KM, Mon NCN, Maw KT, Hlaing MM, Thwin KT, et al. Hepatitis B and C Viral Infections among Hemodialysis Patients Attending 500 Bedded Yangon Specialty Hospital, Myanmar. Myanmar Health Sciences Research Journal. 2018; 30 (3): 213-217.

37. Aye NS, Oo MM, Harries AD, Mon MM, Hone S, Oo HN, et al. HIV, HBV and HCV in people who inject drugs and are placed on methadone maintenance therapy, Yangon, Myanmar. PUBLIC HEALTH ACTION. 2018;8(4):202-10. doi: 10.5588/pha.18.0050. PubMed PMID: WOS:000457790500010.

38. Ngo CC, Maidment C, Atkins L, Eagar S, Smith MM. Blood screen findings in a 2-year cohort of newly arrived refugees to Sydney, Australia. Public Health Res Pract. 2018;28(1). Epub 2018/03/28. doi: 10.17061/phrp2811804. PubMed PMID: 29582037.

39. Juon HS, Ha E, Kim F, Trang A, Pan J, Blanchard J. Prevalence of Viral Hepatitis in Foreign-Born Populations in the Baltimore-Washington Metropolitan Area, 2009-2015. JOURNAL OF COMMUNITY HEALTH. 2019;44(2):203-7. doi: 10.1007/s10900-018-0573-2. PubMed PMID: WOS:000460944900002.

40. Ye M, Chen X, Wang Y, Duo L, Zhang C, Zheng YT. Identification of a New HCV Subtype 6xg Among Injection Drug Users in Kachin, Myanmar. Front Microbiol. 2019;10:814. Epub 2019/05/07. doi: 10.3389/fmicb.2019.00814. PubMed PMID: 31057519; PubMed Central PMCID: PMCPMC6482298.

41. Chen M, Ma Y, Chen H, Dai J, Luo H, Jia M, et al. Complete genome sequencing and evolutionary analysis of HCV subtype 6xg from IDUs in Yunnan, China. PLoS One. 2019;14(5):e0217010. Epub 2019/05/17. doi: 10.1371/journal.pone.0217010. PubMed PMID: 31095618; PubMed Central PMCID: PMCPMC6522032.

42. Myanmar Integrated Biological and Behavioural Surveillance Survey and Population Size Estimates among People Who Inject Drugs (PWID) 2017-2018. National AIDS Programme (NAP), Myanmar Ministry of Health and Sports, Myanmar; 2019.

43. Win TS, Maw SY, Kyaw YY, Maw YY. Serodetection of Hepatitis C Virus Infection among HIV Patients in Mingalardon Specialist Hospital. Myanmar Health Sciences Research Journal, 2020; 32(1):19-24.

44. Kyaw NTT, Satyanarayana S, Harries AD, Kumar AMV, Kyaw KWY, Phyo KH, et al. Increased All-cause Mortality in People With HIV and Comorbidities: Hepatitis B and C Virus Seropositivity and Hyperglycemia in Myanmar, 2005-2017. Open Forum Infect Dis. 2023;10(1):ofac672. Epub 2023/01/13. doi: 10.1093/ofid/ofac672. PubMed PMID: 36632421; PubMed Central PMCID: PMCPMC9830546.

45. Urban K, Payton C, Mamo B, Volkman H, Giorgio K, Kennedy L, et al. Hepatitis C Screening and Antibody Prevalence Among Newly Arrived Refugees to the United States, 2010-2017. J Immigr Minor Health. 2023:1-8. Epub 2023/03/31. doi: 10.1007/s10903-023-01471-8. PubMed PMID: 36995524; PubMed Central PMCID: PMCPMC10062256.

46. Shinji T, Kyaw YY, Gokan K, Tanaka Y, Ochi K, Kusano N, et al. Analysis of HCV genotypes from blood donors shows three new HCV type 6 subgroups exist in Myanmar. ACTA MEDICA OKAYAMA. 2004;58(3):135-42. PubMed PMID: WOS:000222273300004.

47. Worldometers. Myanmar Population. 2023. [Cited 2023 June 28]. Available from: https://www.worldometers.info/world-population/myanmar-population/.

48. Ousley J, Nesbitt R, Kyaw NTT, Bermudez E, Soe KP, Anicete R, et al. Increased hepatitis C virus co-infection and injection drug use in HIV-infected fishermen in Myanmar. BMC INFECTIOUS DISEASES. 2018;18. doi: 10.1186/s12879-018-3558-y. PubMed PMID: WOS:000453239800001.

49. Zaw SK, Tun ST, Thida A, Aung TK, Maung W, Shwe M, et al. Prevalence of hepatitis C and B virus among patients infected with HIV: a cross-sectional analysis of a large HIV care programme in Myanmar. Trop Doct. 2013;43(3):113-5. Epub 2013/06/27. doi: 10.1177/0049475513493416. PubMed PMID: 23800421.

50. Gondeau C, Pageaux GP, Larrey D. Hepatitis C virus infection: Are there still specific problems with genotype 3? World J Gastroenterol. 2015;21(42):12101-13. Epub 2015/11/18. doi: 10.3748/wjg.v21.i42.12101. PubMed PMID: 26576095; PubMed Central PMCID: PMCPMC4641128.

51. Zhang Z, Yao Y, Wu W, Feng R, Wu Z, Cun W, et al. Hepatitis C virus genotype diversity among intravenous drug users in Yunnan Province, Southwestern China. PLoS One. 2013;8(12):e82598. Epub 2013/12/21. doi: 10.1371/journal.pone.0082598. PubMed PMID: 24358211; PubMed Central PMCID: PMCPMC3866230.

52. Wasitthankasem R, Vongpunsawad S, Siripon N, Suya C, Chulothok P, Chaiear K, et al. Genotypic Distribution of Hepatitis C Virus in Thailand and Southeast Asia. PLOS ONE. 2015;10(5). doi: 10.1371/journal.pone.0126764. PubMed PMID: WOS:000354542500147.

53. Das BR, Kundu B, Khandapkar R, Sahni S. Geographical distribution of hepatitis C virus genotypes in India. Indian J Pathol Microbiol. 2002;45(3):323-8. Epub 2003/06/06. PubMed PMID: 12785176.

54. Islam MS, Miah MR, Roy PK, Rahman O, Siddique AB, Chowdhury J, et al. Genotypes of hepatitis C virus infection in Bangladeshi population. Mymensingh Med J. 2015;24(1):143-51. Epub 2015/03/03. PubMed PMID: 25725681.

Supporting information

S1 Appendix. PRISMA checklist

S2 Table. Database search

S3 Table. Quality appraisal of included studies using the Joanna Briggs Institute checklist for prevalence studies

S4 Checklist. Inclusivity in global research questionnaire

Figure 3

Click here to access/download;Figure;Fig3.tif

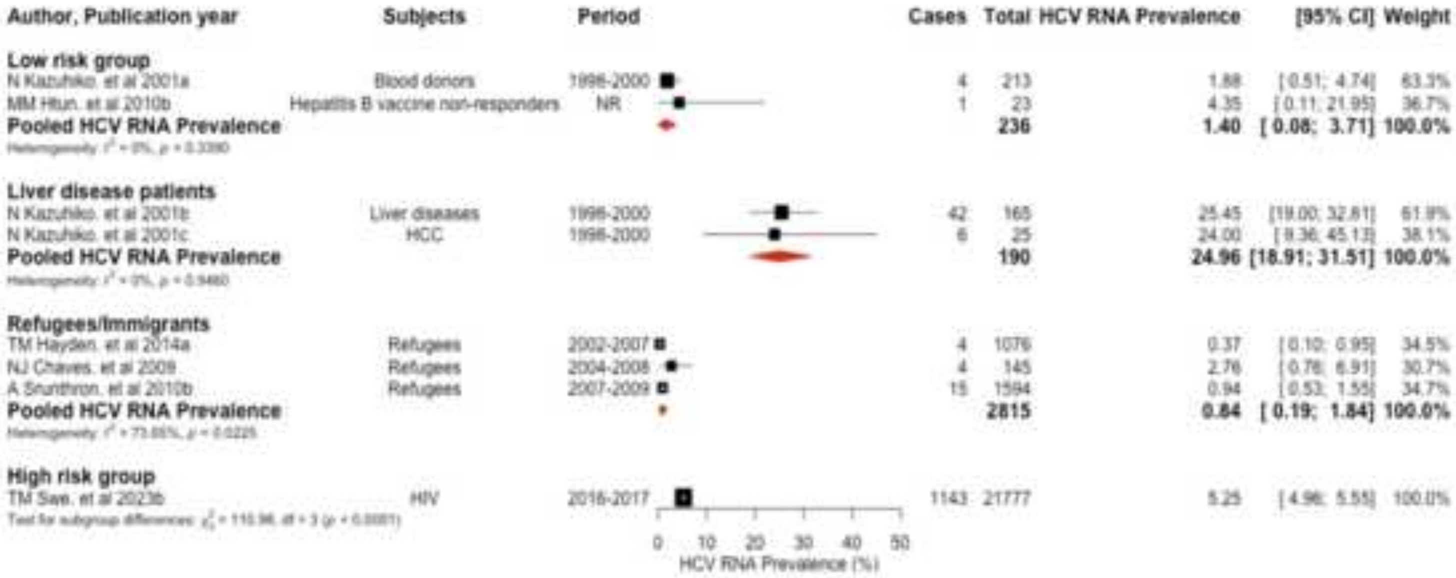

Figure 3: Forest plot of the pooled HCV RNA prevalence in Myanmar, using random effect

Figure 4

Click here to access/download;Figure;Fig4.tif

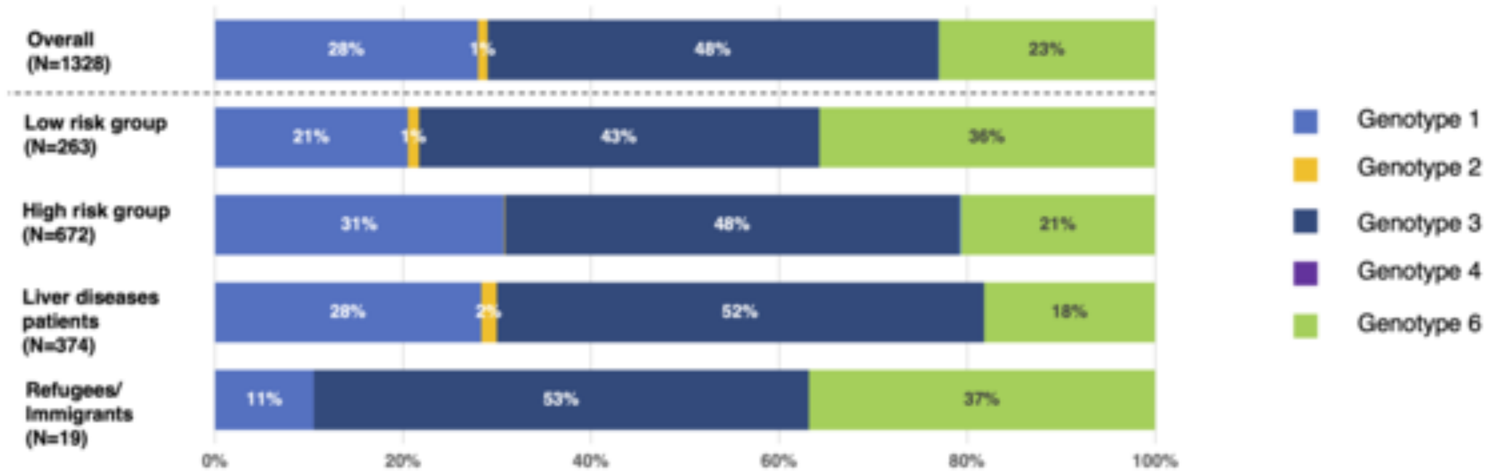

Figure 4: Distribution of HCV genotypes in Myanmar

Figure 1

Click here to access/download;Figure;Fig1.tif

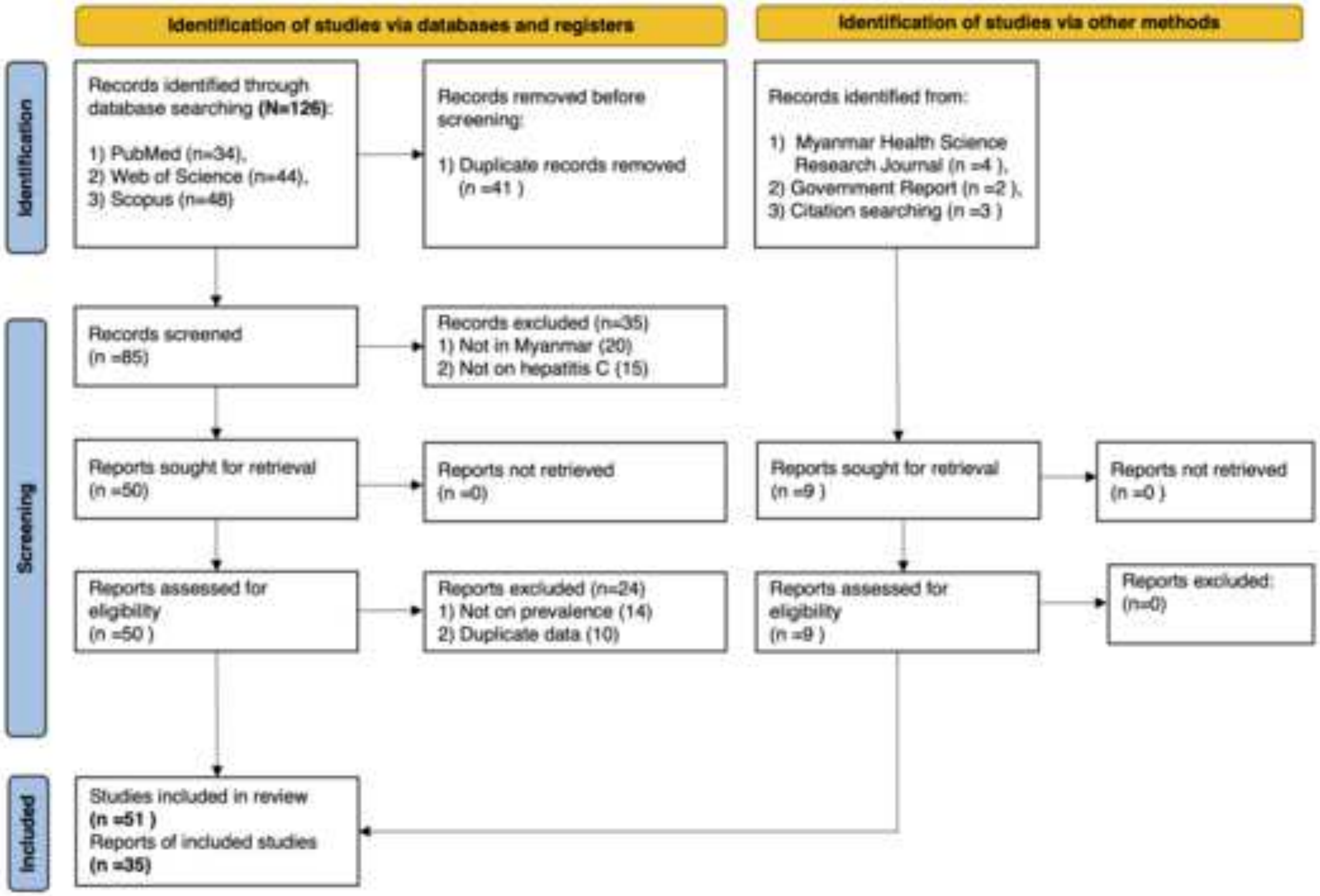

Figure 1: PRISMA showing selection of articles for meta-analysis of HCV in Myanmar

Figure 2

Click here to access/download;Figure;Fig2.tif

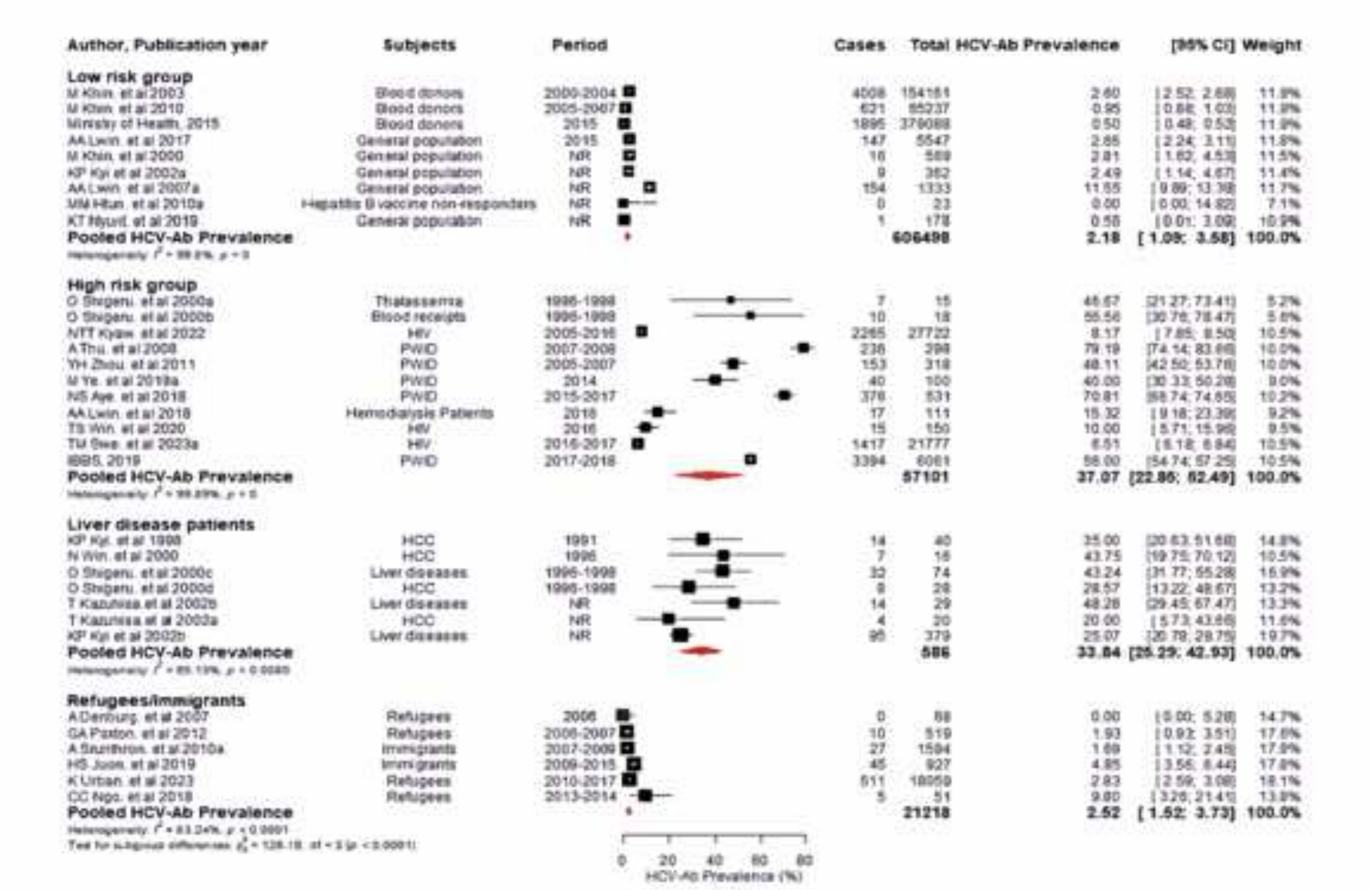

Figure 2: Forest plot of the pooled HCV seroprevalence in Myanmar, using random effect

Figure 5

Click here to access/download;Figure;Fig5.tif

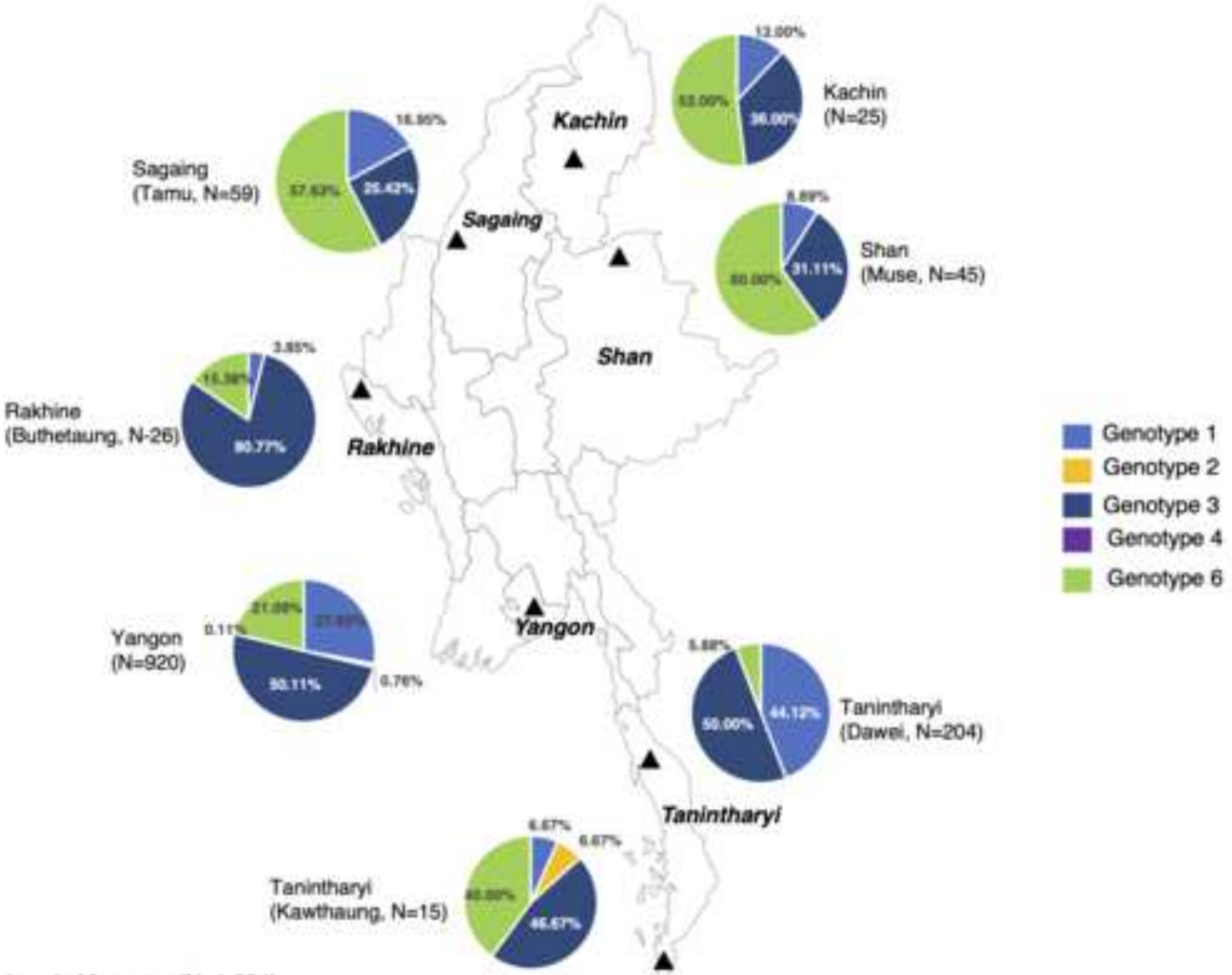

Figure 5: Distribution of HCV genotypes by study place in Myanmar (N=1,294)

Figure 6

Click here to access/download;Figure;Fig6.tif

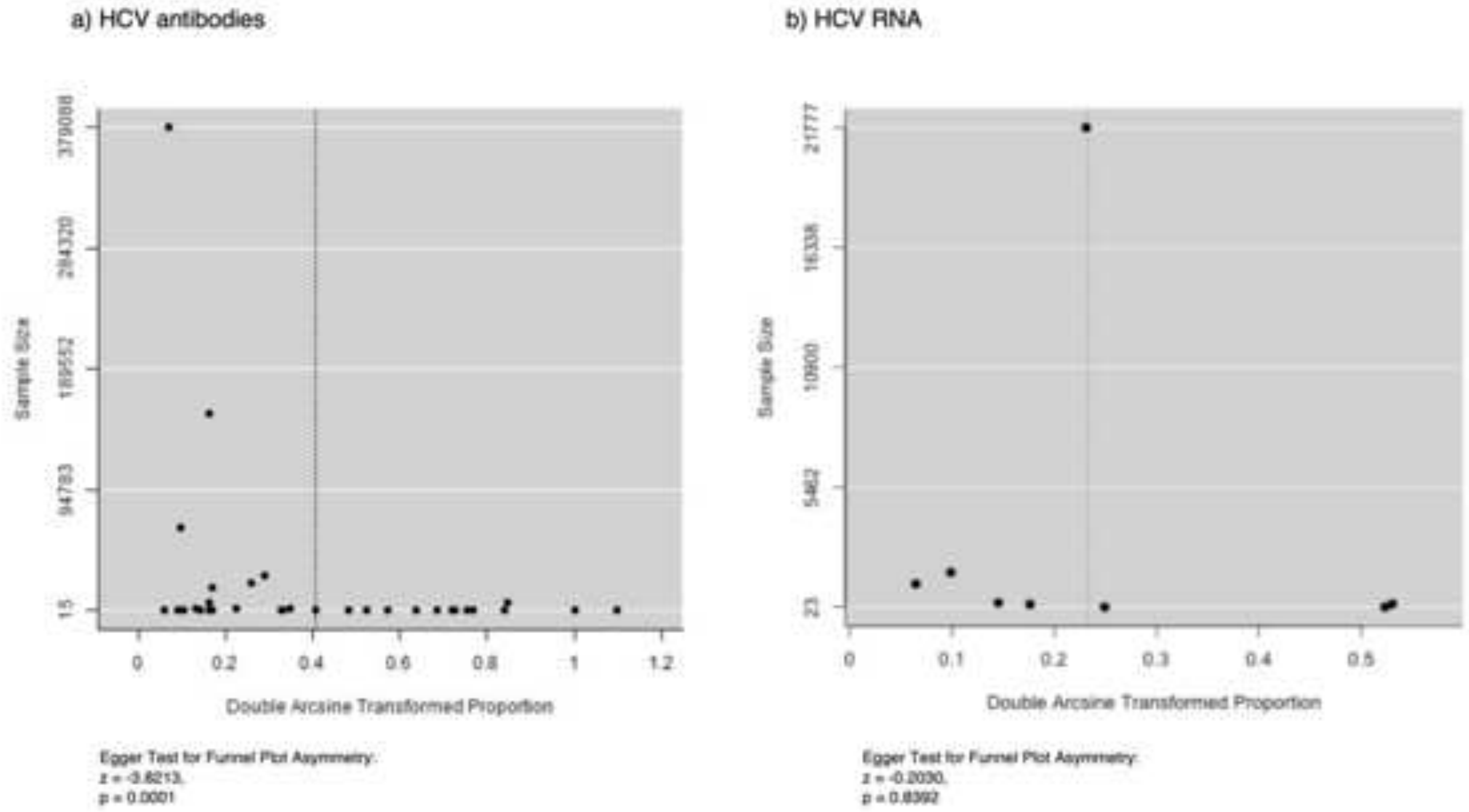

Figure 6: Funnel plot of HCV prevalence a) HCV antibodies b) HCV RNA

Click here to access/download
Supporting Information
S2 Table. Database searchV2.docx

Click here to access/download
Supporting Information
S1 Appendix. PRISMA checklistV2.docx

Click here to access/download
Supporting Information
S3 Table. Quality appraisalV2.docx

Click here to access/download
Supporting Information
S4 Checklist.QuestionnaireV1.docx

Intermediate hepatitis C virus (HCV) endemicity and its genotype distribution in Myanmar: A systematic review and meta-analysis

Formatted: Font: 12 pt

Zayar Phyo[1,2], Ko Ko[1,2], Serge Ouoba[1,2,3], Aya Sugiyama[1,2], Ulugbek Khudayberdievich Mirzaev[1,2,4], Golda Ataa Akuffo[1,2], Chanroth Chhoung[1,2], Tomoyuki Akita[1,2], Junko Tanaka[1,2*]

[1]Department of Epidemiology, Infectious Disease Control and Prevention, Graduate School of Biomedical and Health Science, Hiroshima University, Hiroshima, Japan

[2]Project Research Center for Epidemiology and Prevention of viral hepatitis and hepatocellular carcinoma, Hiroshima University, Hiroshima, Japan

[3]Unité de Recherche Clinique de Nanoro (URCN), Institut de Recherche en Science de la Santé (IRSS), Nanoro, Burkina Faso

[4]Department of Hepatology, Scientific Research Institute of Virology, Tashkent, Uzbekistan

*Corresponding Author

~~**Address for correspondence and reprint requests:**~~

~~Junko Tanaka, PhD~~

~~Department of Epidemiology, Infectious Disease Control and Prevention, Graduate School of Biomedical and Health Sciences Hiroshima University, Hiroshima, Japan~~

~~1-2-3, Kasumi, Minami-ku, Hiroshima, 734-8551, Japan~~

~~Tel: +81-82-257-5160~~

~~Fax: +81-82-257-5164~~

Email: jun-tanaka@hiroshima-u.ac.jp (JT)

Abstract ~~(338/300)~~

~~**Background:**~~ Comprehensive details on Hepatitis C virus (HCV) infection in Myanmar are lacking. This study determined the prevalence of~~estimated~~ HCV antibodies and ribonucleic acid (RNA) ~~prevalence~~ and ~~genotype~~ the distribution of HCV genotypes across~~in~~ different~~various~~ populations in Myanmar from~~between~~ 1990 to~~and~~ 2023.

~~**Methods:**~~ A systematic search in PubMed, Web of Science, Scopus, and local journals identified studies reporting on HCV antibodies, RNA, and genotypes, excluding clinical research related to liver disease prognosis. Screening and data extraction was done by two authors ~~A systematic search in PubMed, Web of Science, Scopus, and local journals, identified studies from January 1990 to May 2023 that reported data on HCV antibodies, RNA, and genotypes, excluding clinical research related to liver disease prognosis.studies on HCV prevalence or genotypes for inclusion. All relevant data were extracted using Microsoft Excel, with two authors,~~ and study populations were categorized into low-risk, high-risk, liver disease patients, and refugees outside the country. The pooled prevalence was performed by Dersimonian and Laird method using the R

Formatted: Font: 18 pt, Font color: Text 1

Formatted: Font: 16 pt

Formatted: Indent: First line: 0.5"

Formatted: Font: 16 pt

program. The publication bias was shown by funnel plot, the Egger test was used to assess the symmetry of the plot, and the heterogeneity was examined by the Cochran Q test and I^2 index.

~~**Results:**~~ Out ~~of a total~~ of 135 reports screened for eligibility, 35 reports comprising 51 studies were included in which 33 studies provided data on HCV seroprevalence in 685,403 individuals, 8 studies reported HCV RNA prevalence in 25,018 individuals, and 10 studies examined HCV genotypes in 1,845 individuals. The pooled seroprevalence of HCV among low-risk, high-risk, liver disease patients and refugees were 2.18%, 37.07%, 33.84%, and 2.52% respectively. HCV RNA-positive rates in ~~low-risk, high-risk, liver disease patients and refugees~~these groups were 1.40%, 5.25%, 24.96%, and 0.84% respectively. Seroprevalence studies showed publication bias (Egger test, p=0.0001), while RNA studies did not (Egger test, p=0.8392). HCV genotype 3 was predominant in all sub-groups in Myanmar~~.~~.

~~**Conclusion**~~: ~~This systematic review and meta-analysis~~Our study shows~~explore~~ ~~indicated~~d ~~that~~ Myanmar has intermediate HCV endemicity with lowest HCV prevalence of 2.18% in ~~low risk~~low-risk groups and highest prevalence of 37.07% in high- risk groups. ~~of HCV seroprevalence in the low-risk group with a decreasing trend over the past 20 years due to measures such as blood donor screening, infection control practices, and increased awareness through education programs.~~ However, the findings highlight the need for further epidemiological studies to understand ~~the~~ actual disease burden and implement effective countermeasures to achieve the WHO's goal of HCV elimination by 2030.

Keywords: Seroprevalence, Hepatitis, HCV, HCV-Ab, HCV RNA, HCV genotype, Myanmar, Burma, Systematic review, Meta-analysis.

Formatted: Font: 16 pt, Not Bold

Formatted: Font: 16 pt

Formatted: Font: 16 pt, Not Bold

Formatted: Font: 16 pt

Formatted: Left, Indent: First line: 0.5", Space Before: 0 pt, Widow/Orphan control, Adjust space between Latin and Asian text, Adjust space between Asian text and numbers

Formatted: Font color: Auto

Formatted: Font color: Auto

Formatted: Font: 16 pt, Font color: Auto

68

Formatted: Font: 18 pt

Background

Hepatitis C virus (HCV) is an enveloped positive-strand RNA virus belonging to the genus Hepacivirus in the family Flaviviridae. It causes liver inflammation, which can range from mild to severe, including cirrhosis and liver cancer. HCV is a bloodborne virus transmitted through exposure to infected blood, which can occur via unsafe injection practices, unsafe healthcare, unscreened blood transfusions, injection drug use, and sexual practices that involve contact with contaminated blood~~Hepatitis C virus (HCV) infection is a liver inflammation that ranges from mild illness to severe lifelong conditions like cirrhosis and cancer.~~ ~~It is primarily a bloodborne virus, with infections typically resulting from unsafe injection practices, inadequate healthcare, unscreened blood transfusions, and certain risky behaviors involving blood exposure that mainly affects developing countries~~[1]. Globally, ~~HCV infection affects~~ 58 million people are infected by HCV infection with approximately ~~and annually~~1.5 million new infections ~~are estimated to be around 1.5 million~~each year [1]. According to the World Health Organization (WHO), ~~reported that~~only 20% of those infected with HCV ~~infected people~~ have~~d~~ been diagnosed, leaving a significant number ~~and a huge proportion of infected people remained~~ undiagnosed cases because of limited~~lack of~~ access to affordable testing [2]. The imperceptible barriers were still present in line with the elimination of viral hepatitis as a global action, rising as a public health concern. As for WHO Southeast Asia region i~~I~~n 2020, there was an estimated 10 million people living with chronic hepatitis C, resulting in ~~WHO Southeast Asia region had an estimated 10 million people living with chronic hepatitis C with an attributable~~ 40,938 deaths annually~~–~~ and~~and only~~ ~~–~~less than 1% of diagnosed viremic ~~diagnosed~~ patients had ~~been initiated~~started treatment [3].

Myanmar, a developing country in Southeast Asia, is rich in natural resources and home to a diverse population of 54.78 million people from various ethnic groups. ~~In Myanmar, liver-related research was started in 1972 and the liver research unit was established in 1973. The blood donor screening for HCV antibodies was started in 2000 and the awareness on prevention of viral hepatitis was enhanced in 2011. In 2013 and 2014, the Gastro-Intestinal and Liver Society of Myanmar Medical Association (MMA), a non-profit local organization developed the Viral Hepatitis Clinical Management Guidelines. After the establishment of the National Hepatitis Control Program (NHCP) in November 2014, the prevention, diagnosis, and treatment of viral hepatitis have been enhanced in Myanmar following the WHO's 2014 resolution strategies. Later the NHCP adopted the National Strategic Plan (2016-2020)(4) adhering to the world's vision to halt the transmission of~~

Formatted: Strikethrough

Formatted: Strikethrough

Formatted: Strikethrough

~~viral hepatitis and ensuring everyone living with viral hepatitis has access to safe, affordable, and effective care and treatment. National Simplified Treatment Guideline for HCV infection was released in February 2017(5) and then direct-acting antivirals (DAAs) were used for the treatment of HCV infection.~~

Formatted: Strikethrough

Formatted: Strikethrough

Before 2017, there was no national program or strategic plan ~~was~~ in Myanmar providing free-of-charge treatment for~~of~~ HCV. ~~a~~Although ~~the testing of~~ HCV antibodies and HCV RNA testing were~~as~~ ~~expensively~~ available in private hospitals and ~~laboratorie~~laboratories, they are costly [4]~~s~~. With the ~~widespread availability~~introduction of direct-acting antivirals (DAAs) which have a high success rate (>90% sustained virologic response: SVR) and reported fewer side effects, the Myanmar government ~~with a high success rate (>90% sustained virologic response: SVR), the Government of Myanmar~~ initiated the free testing and ~~the free-of-charge~~ antiviral treatment ~~for HCV~~ in seven public hospitals in 2017 [5]. This made Myanmar the ~~which has been denoted as a very~~ first country in Southeast Asia region providing free~~-of-charge~~ HCV treatment. ~~in South-East Asia region. In Myanmar,~~ V~~v~~arious local and international non-governmental organizations have also joined~~taken part in the~~ efforts to eliminate HCV in Myanmar [6]. The Myanmar Liver Foundation (MLF) has been offering anti-HCV treatment to patients with chronic liver diseases through their Than Sitt Charity Clinics since 2017 [7]. Médecins Sans Frontières (MSF) has been providing anti-HCV treatment specifically to individuals co-infected with HIV in Myanmar since the same year [8]. Additionally, the Burnet Institute of Myanmar has been delivering HCV treatment to both the general population and people who inject drugs (PWID) since 2019 ~~One prominent organization involved in this endeavor is the Myanmar Liver Foundation (MLF), which has been offering anti-HCV treatment to patients with chronic liver diseases since 2017 through their Than Sitt Charity Clinics. Additionally, Medecins Sans Frontieres (MSF) has been providing anti-HCV treatment specifically to individuals who are co-infected with HIV since 2017. Another organization actively engaged in combating HCV is the Burnet Institute of Myanmar. Since 2019, they have been delivering HCV treatment not only to the general population but also to people who inject drugs (PWID)~~[6].

~~In Myanmar, a country in South East Asia, the~~The epidemiological patterns of HCV infection in Myanmar are not well-documented. Between 1996 and 2017, reported prevalence rates of HCV antibodies varied widely, from the lowest 0.50% among blood donors (from an unpublished study) to the highest 79.19% [9] among people who inject drugs (PWID). ~~In Myanmar, an estimated nationwide prevalence of HCV antibodies was 2.65%(7) in 2015 suggesting that 1.4 million people are exposed to HCV infection. Considering that 30% of infected persons spontaneously clear the virus within 6 months of infection without treatment(1), the total number of chronic HCV infections was estimated to be 0.9 million. From 1996 through 2017, some studies~~

Formatted: Not Highlight

Formatted: Not Highlight

reported the prevalence of HCV antibodies ranging from the lowest of 0.50% among blood donors (unpublished study) to the highest of 79.19%(8) among PWID. Most of the studies are conducted in a convenient sampling strategy so that the external validation and representativeness of the target population are questionable. TheA only nationwide study in -2015 estimatedestimated the prevalence of HCV antibodies at -in Myanmar was 2.65% [10] in 2015, suggesting that indicating that approximately 1.4 million people werehad been exposed to the hepatitis C virusHCV. Given that about 30% of those infected naturally eliminate the virus within six months withoutt any treatment [1], the total number of chronic HCV infections was estimated to be artound at 0.9 million. These chronic infections can progress to severe complications such as liver cirrhosis and hepatocellular carcinoma (HCC) [11]. Since 2018, there have been no new studies have been conducted on the burden of HCV infection in Myanmar. Between 1996 and 2017, reported prevalence rates of HCV antibodies varied widely, from a low of 0.50% among blood donors (from an unpublished study) to a high of 79.19%(9) among people who inject drugs (PWIDT-the external validity and representativeness of theese findings for the broader population remain uncertain as most of the existing studies employed the convenience sampling methods [12]., the external validity and representativeness of these findings for the broader population remain uncertain.

Since 2018, tthat beganseverehindering (10)disruption may (11)(11)MoreoverFurthermore, the absencelack of detailed information on HCV infection characteristics within specific populations and geographic regions presentsposes a significant challenge for the effectively implementiimplementation ofng targeted control strategies [13]. Therefore, Moreover, no studies related to the burden of HCV infection in Myanmar were found after 2018. Additionally, the ongoing political situation since 2021 disrupted the existing healthcare system and interrupted all the strategic plans that had been developed before. Hence, a comprehensive analysis of data spanning over three decades is needed to identify the situation of HCV infection in Myanmar and its potential impact on public health. Furthermore, by examining the distribution of HCV genotypes, the genetic diversity of the virus circulating among different populations can be elucidated. To provide the baseline epidemiological data on the disease burden of HCV infection in specific populations and the detailed characteristics in Myanmar, this study aimed to estimate the prevalence of HCV antibodies and HCV RNA and the distribution of HCV genotypes in various populations in Myanmar between 1990 and 2023.this study was conductedaims to establish provide the baseline epidemiological data on the disease burden of HCV infection in Myanmar and to determine , aiming to estimate the prevalence of HCV antibodies and HCV RNA, as well as and to assess the distribution of HCV genotypes across various populations from 1990 to 2023.

Formatted: Not Highlight

Formatted: Not Highlight

Formatted: Not Highlight

Formatted: Not Highlight

Formatted: Not Highlight

Field Code Changed

Formatted: Not Highlight

Formatted: Not Highlight

Formatted: Not Highlight

Formatted: Not Highlight

Formatted: Not Highlight

Formatted: Not Highlight

Field Code Changed

Formatted: Not Highlight

Formatted: Not Highlight

Formatted: Not Highlight

Formatted: Not Highlight

Formatted: Not Highlight

Formatted: Not Highlight

Formatted: Not Highlight

Formatted: Not Highlight

Formatted: Not Highlight

Formatted: Not Highlight

Formatted: Not Highlight

Formatted: Not Highlight

Formatted: Not Highlight

Formatted: Not Highlight

Formatted: Not Highlight

Materials and method

Study design and guidelines

This systematic review was conducted following the 2020 Preferred Reporting Items for Systematic Reviews and Meta-Analysis (PRISMA) guidelines [14] (~~Additional file 1~~S1 Appendix~~Table~~). Though we did not prospectively register our systematic review in the registry database such as International Prospective Register of Systematic Reviews (PROSPERO), International Platform of Registered Systematic Review and Meta-analysis Protocols (INPLASY) etc, there was no ongoing similar studies in ~~those database~~those databases. Before the review, we searched databases such as PubMed, Web of Science, and Scopus for published studies on this topic and found none.~~,"The study was not registered in the International Prospective Register of Systematic Reviews (PROSPERO) but we have checked PROSPERO for similar ongoing studies and confirmed that there are no ongoing studies that are similar to ours.~~

Data source and study selection

~~We s~~Searches in~~ed~~ PubMed, Web of Science, and Scopus were conducted to identify the relevant records (S2 Table). Our review included studies conducted from January 1990 to May 2023 that reported on the prevalence of HCV antibodies, HCV RNA, and/or HCV genotypes, specifically including exact sample sizes and the number of positive cases.~~To identify relevant records, we conducted searches in PubMed, Web of Science, and Scopus. The eligible studies included in our review were conducted between January 1990 and May 2023, reporting the prevalence of HCV antibodies, HCV RNA, and/or HCV genotype with exact sample sizes and the number of positive cases.~~ There were no restrictions on the type of study, and the subjects could be Myanmar citizens living inside or outside the country. However, clinical research or studies focusing on the prognosis of liver diseases in HCV-infected patients were excluded. Other factors such as gender, age, work nature, and place of living did not affect the eligibility. Only reports in English were considered, and manual searches were conducted in The Myanmar Health Sciences Research Journal, Google Scholar, reference lists of eligible reports, and government reports. The last search was conducted on May 31, 2023, and duplicate records were removed using the Rayyan – Intelligent Systematic Review

Formatted: Font: 18 pt

Formatted: Font: 18 pt

Formatted: Font: 16 pt

Formatted: Font: (Default) Times New Roman, 10 pt

Formatted: Font: (Default) Times New Roman, 10 pt

Formatted: Font: (Default) Times New Roman, 10 pt

Formatted: Font: (Default) Times New Roman, 10 pt

Formatted: Font: 14 pt

tool. Two independent investigators (ZP and KK) screened the records based on titles and abstracts, and relevant reports were retained for full-text review. In cases of disagreements, a third investigator (JT) made the final decision.

Data extraction

Formatted: Font: 14 pt

All relevant raw data, including authors' names, year of publication, study design, sampling method, study period, characteristics of study subjects (age, sex, location, comorbidities), sample size, number of participants positive for HCV antibodies/HCV RNA/HCV genotype, and type of biological assay, were extracted from each recruited study using Microsoft Excel 2010. The data were counter-checked by a second author. In this study, a "report" refers to any document mentioning HCV prevalence/genotype data, while a "study" pertains to the measurement of HCV antibodies/HCV RNA/HCV genotype in a specific population. Consequently, one report may describe multiple studies, and data were extracted separately for each study. In cases where study subjects were found to overlap in two or more different studies, only one publication was included.

Quality Assessment of the Included Studies

Formatted: Font: 14 pt

The Joanna Briggs Institute checklist for prevalence studies was used to assess the quality of the included studies The methodological quality of the prevalence studies was assessed using the Joanna Briggs Institute checklist for prevalence studies[15] (Additional file 2:S32 Table S2). The review process unified the minimum acceptable information for each variable. The minimum sample size for prevalence estimation was set at 312 subjects, calculated based on an assumed seroprevalence of 2.65% [10], a precision of 1.8%, and a confidence level of 95%. The sampling procedure of each study was examined in detail, and studies employing probabilistic sampling were considered representative of the target population. The validity of seroprevalence and genotype data relied on whether the diagnostics were based on standard procedures and methods.

Formatted: Font color: Red

Field Code Changed

Figure 1: PRISMA showing selection of articles for meta-analysis of HCV in Myanmar

Statistical Analysis

Formatted: Font: 16 pt

The study populations were categorized into four groups based on the characteristics of subjects: (1) low-risk

group, (2) high-risk group, (3) liver disease patients, and (4) refugees/immigrants. The low-risk group included the general population, blood donors, and hepatitis B virus (HBV) vaccine non-responders. The high-risk group contained people living with human immunodeficiency virus (PLHIV), PWID, patients who received blood transfusions, and hemodialysishaemodialysis patients. The other two groups were simply categorized as liver disease patients and refugees/immigrants. The statistical analysis was conducted using the 'meta' and 'metafor' packages in R programming. The Freeman-Tukey double arcsine method was used to transform the data to accommodate small proportions.To accommodate small proportions, the data were transformed using the Freeman-Tukey double arcsine method. The meta-analysis was performed using the Dersimonian and Laird method, which utilizes a random-effects model, and the results were presented in a forest plot. For the low-risk group, which included three unpublished papers with a total sample size of 72,680 xxxx, a sensitivity analysis was performed by excluding these unpublished papers. Confidence intervals (CI) for the proportions of individual studies were calculated using the Clopper-Pearson method. The Cochran Q test was employed to evaluate heterogeneity and then its degree was quantified by , and the I^2 index was used to quantify the degree of heterogeneity. To evaluate heterogeneity, the Cochran Q test was employed, and the degree of heterogeneity was quantified using the I^2 index. Heterogeneity was considered significant if the p-value of the Cochran Q test was less than 0.05. The high, medium, or low level of heterogeneity was identified by the I^2 index thresholds of 75%, 50%, and 25% respectively [16]. A funnel plot of the transformed proportion against the sample size was generated to assess publication bias To assess publication bias, a funnel plot of the transformed proportion against the sample size was generated[17]. The symmetry of the plot was evaluated using the Egger test, with significance determined at a threshold of $p < 0.1$.

Inclusivity in global research

Additional information regarding the ethical, cultural, and scientific considerations specific to inclusivity in global research is included in the Supporting Information (S4 Checklist).

Results

Search results

A total of 126 articles were identified through the database search. Nine articles from the Myanmar Health

Formatted: Not Highlight

Formatted: Superscript

Formatted: Font: 10 pt, Not Bold

Formatted: Font: 18 pt

Formatted: Font: 16 pt

Sciences Research Journal and government reports were manually added. After removing the duplication and irrelevant articles, 85 articles were screened by their abstracts ~~and~~ resulting in the ex~~ex~~clusion of 35 articles ~~were excluded under~~based on the strict exclusion criteria. ~~And then,~~The remaining 50 articles were accessed for full text review and carefully ~~reviewed~~ evaluated for ~~its~~ eligibility. Ultimately, ~~Thirty-five~~35 ~~Finally, 35~~ articles ~~that~~ met the ~~eligible~~ criteria and ~~, and~~ were included in the analysis to estimate~~ating~~ the prevalence of HCV antibodies, ~~and~~ HCV RNA and the distribution of HCV genotype ~~distribution~~ in Myanmar (Fig~~ure~~ 1). Some studies indicated the exact number of males and females or the place from which the samples were taken and also stated the age-specific number of samples and number of positive participants and corresponding prevalence, but most of the studies had lack of such information and it was quite difficult to divide the study subjects by their sex, age, and place of living. In terms of time series for included reports, 5, 9, and 13 reports were conducted during 1990-2000, 2001-2010, and 2011-2020 respectively but 8 reports did not describe the study year [18-24] (Table 1).

Figure 1: PRISMA showing a selection of articles for meta-analysis of HCV in Myanmar

Legend: This figure shows the selection process of the articles for the estimation of HCV prevalence in Myanmar. Each arrow indicates the step-by-step process for recruiting the articles and the detailed reason for the exclusion of articles were described with their respective exact number. This figure was built following the guideline for 2020 Preferred Reporting Items for Systematic Reviews and Meta-Analysis (PRISMA).

Formatted: Font color: Text 1, Not Highlight
Formatted: Font color: Text 1
Formatted: Font color: Text 1, Not Highlight
Formatted: Font color: Text 1
Formatted: Font color: Text 1, Not Highlight
Formatted: Font color: Text 1
Formatted: Font color: Text 1, Not Highlight
Formatted: Font color: Text 1
Formatted: Font color: Text 1, Not Highlight
Formatted: Font color: Text 1
Formatted: Font color: Text 1, Not Highlight
Formatted: Font color: Text 1
Formatted: Font color: Text 1, Not Highlight
Formatted: Font color: Text 1
Formatted: Font color: Text 1
Formatted: Font color: Text 1
Formatted: Font: Italic

Table 1: Characteristics of included studies reporting the prevalence of HCV and genotype distribution in Myanmar between 1990 and 2023

First author, year of publication	Study design	Sampling method	Data collection period	Study subjects	Mean age or range (in years)	Sample size	Number of HCV-Ab positive	Number of HCV RNA-positive	Number of amplified samples	Assay for HCV-Ab/RNA testing
J Mellor, 1996[18]	C	Convenient	NR	Blood donors	NR	8	-	-	8	-
KP Kyi, 1998[25]	CS	Consecutive	1991	HCC	20-89	40	14	-	-	ELISA
M Khin, 2000[†]	CS	Convenience	NR	General population	1-74	569	16	-	-	NR
O Shigeru, 2000a[△][26]	CS	NR	1996-1998	Thalassemia	7±3.5	15	7	-	-	ELISA
O Shigeru, 2000b[△][26]	CS	NR	1996-1998	Blood receipts	20±9	18	10	-	-	ELISA
O Shigeru, 2000c[△][26]	CS	NR	1996-1998	Liver diseases*	3-60	74	32	-	-	ELISA
O Shigeru, 2000d[△][26]	CS	NR	1996-1998	HCC	47±12	28	8	-	-	ELISA
N Win, 2000[27]	CS	NR	1996	HCC	23-70	16	7	-	-	ELISA
N Kazuhiko, 2001a[△][28]	CS	Convenience	1998-2000	Blood donors	7-80	213	-	4	-	PCR
N Kazuhiko, 2001bd[△][28]	CS	Convenience	1998-2000	Liver diseases*	7-80	165	-	42	24	PCR
N Kazuhiko, 2001c[△][28]	CS	Convenience	1998-2000	HCC	7-80	25	-	6	-	PCR

First author, year of publication	Study design	Sampling method	Data collection period	Study subjects	Mean age or range (in years)	Sample size	Number of HCV-Ab positive	Number of HCV RNA-positive	Number of amplified samples	Assay for HCV-Ab/RNA testing
T Kazuhisa, 2002a△[20]	CS	NR	NR	Liver diseases*	NR	29	14	-	-	IRMA
T Kazuhisa, 2002b△[20]	CS	NR	NR	HCC	NR	20	4	-	-	IRMA
KP Kyi, 2002a△[19]	CS	Consecutive	NR	General population	NR	362	9	-	-	Immunoblot
KP Kyi, 2002b△[19]	CS	Convenience	NR	Liver diseases*	NR	379	95	-	-	Immunoblot
M Khin, 2003†	CS	Consecutive	2000-2004	Blood donors	NR	154,161	4,008	-	-	NR
T Shinji, 2004[20]	RC	Convenient	2000	Blood donors	18-51	201	-	-	110	-
AA Lwin, 2007ab△[21]	CS	Convenience	NR	General population	1-75	1,333	154	-	145	PA
A Denburg, 2007[29]	C	Consecutive	2006	Refugees	NR	68	0	-	-	NR
A Thu, 2008[9]	CS	Convenience	2007-2008	PWID	29.49±6.3	298	236	-	-	Rapid test
NJ Chaves, 2009[30]	RC	Consecutive	2004-2008	Refugees	16-86	145	-	4	-	PCR
M Khin, 2010[31]	CS	Consecutive	2005-2007	Blood donors	18-60	65,237	621	-	-	Rapid test
A Srunthron, 2010abc△[32]	CS	Consecutive	2007-2009	Immigrants	15-60	1,594	27	15	15	ELISA (HCV-Ab)

First author, year of publication	Study design	Sampling method	Data collection period	Study subjects	Mean age or range (in years)	Sample size	Number of HCV-Ab positive	Number of HCV RNA-positive	Number of amplified samples	Assay for HCV-Ab/RNA testing
										PCR (HCV RNA)
MM Htun, 2010ab△[22]	CS	Consecutive	NR	HBV vaccine non-responders	NR	23	0	1	-	PA (HCV-Ab) PCR (HCV RNA)
YH Zhou, 2011[33]	CS	Convenience	2005-2007	PWID	31.8±9.8	318	153	-	-	ELISA
GA Paxton, 2012[34]	CS	Consecutive	2007-2009	Refugees	0.5-87	519	10	-	-	NR
TM Hayden, 2014ab△[35]	CS	Convenience	2002-2007	Refugees	15-95	1,076	-	4	4	PCR
MOH Myanmar, 2015†	CS	Consecutive	2015	Blood donors	15-80	379,088	1,895	-	-	Rapid test
C Naing, 2015[23]	RC	Convenient	NR	Chronic hepatitis C	47.1±11.6	362	-	-	350	-
AA Lwin, 2017[10]	CS	Random	2015	General population	48 ± 14.6	5,547	147	-	-	ICT
AA Lwin, 2018[36]	CS	NR	2016	Haemodialysis patients	20-40	111	17	-	-	ICT
NS Aye, 2018[37]	RC	NR	2015-2017	PWID	24-34	531	376	-	-	Rapid test
CC Ngo, 2018[38]	RC	Consecutive	2013-2014	Refugees	30-70	51	5	-	-	NR

HS Juon, 2019[39]	CS	Convenience	2009-2015	Immigrants	37.3 ± 13	927	45	-	-	NR
M Ye, 2019ab△[40]	CS	NR	2014	PWID	16-74	100	40	-	25	ELISA
M Chen, 2019[41]	CS	Convenient	2014	PWID	22-33	7	-	-	7	-
Myanmar IBBS, 2019[42]	CS	RDS	2017-2018	PWID	34.62 ± 9.25	6,061	3,394	-	-	Rapid test
KT Nyunt, 2019[24]	CS	NR	NR	General population	35.3	178	1	-	-	ICT
TS Win, 2020[43]	CS	NR	2016	PLHIV	35 (30-42)	15	150	-	-	ELISA
NTT Kyaw, 2022[44]	C	Consecutive	2005-2016	PLHIV	1-65	27,722	2,265	-	-	OraQuick
K Urban, 2023[45]	CS	Consecutive	2010-2017	Refugees	NR	18,059	511	-	-	NR
TM Swe, 2023abc△[8]	C	Consecutive	2016-2017	PLHIV	15-80	21,777	1,417	1,143	640	OraQuick (HCV-Ab)<br>PCR (HCV RNA)

HCV-Ab, hepatitis C virus antibodies; *HCV RNA*; hepatitis C virus ribonucleic acid; *MOH*, Ministry of Health; *IBBS*, integrated biological and behavioral survey; *CS*, cross-sectional; *C*, cohort; *RC*, retrospective cohort; *NR*, not reported; *RDS*, respondent driven sampling; *HBV*, hepatitis B virus; *HCV*, hepatitis C virus; *HCC*, hepatocellular carcinoma; *PWID*, people who inject drugs; *PLHIV*, people living with human immunodeficiency virus; *ELISA*, enzyme-linked immunosorbent assay; *PCR*, polymerase chain reaction; *ICT*, immunochromatography; *IRMA*, immunoradiometric assay; *CLIA*, chemiluminescence immunoassay; *PA*, particle agglutination.

* Patients with liver diseases other than hepatocellular carcinoma; † Unpublished observations, △Studies issued from the same report.

Study characteristics

Formatted: Font: 16 pt

Out of a total of 135 reports screened for eligibility, 35 reports comprising 51 studies were included in which 33 studies provided data on HCV seroprevalence in 685,403 individuals, 8 studies reported HCV RNA prevalence in 25,018 individuals, and 10 studies examined HCV genotypes in 1,857 individuals. The studies included in the analysis were conducted from 1991 to 2018 (Table 1). In HCV antibodies and RNA prevalence studies, the cross-sectional design was primarily used in 34 out of 41 studies (82.9%), while only one study [10] (2.4%) employed a probabilistic sampling method. In terms of sample size, 53.7% (22/41) of the studies included adequate sample sizes (>312). HCV antibodies and HCV RNA were determined through biological assays which were reported in 83.0% (34/41) of the studies (~~Additional file 1:~~S2 Table~~ S2~~).

Regarding the estimation of HCV seroprevalence, 28 out of 33 studies conducted cross-sectional studies, two studies utilized retrospective cohort designs in which the prevalence was estimated from laboratory records, and the remaining three studies employed cohort studies where HCV seroprevalence was determined as part of the overall study (Table 1). For the HCV RNA prevalence study, six out of eight studies performed cross-sectional studies, one study adopted a retrospective cohort, and one study employed a cohort study (Table 1). Out of the ten studies conducted for the HCV genotype study, five utilized cross-sectional study designs, three conducted a retrospective cohort approach, and two employed a cohort study methodology (Table 1). All included reports covered a study period ranging from 1991 to 2018, with the majority of studies conducted after 2010.

In terms of participant characteristics in the HCV seroprevalence estimate, nine studies focused on low-risk groups, including the general population, blood donors, and individuals who did not respond to HBV vaccines, encompassing a total of 60,648 participants. Eleven studies targeted high-risk groups, such as PWID, PLHIV, patients who received blood transfusions, and individuals undergoing hemodialysis, with a total of 57,101 participants. Seven studies focused on liver disease patients, specifically hepatocellular carcinoma (HCC), liver cirrhosis, and acute/chronic hepatitis, including 586 patients. Finally, six studies targeted refugees/immigrants, comprising a total of 21,218 individuals (Table 1).

In the HCV RNA prevalence study, two studies focused on low-risk groups, including blood donors and individuals who did not respond to HBV vaccines, with a total of 236 subjects. One study targeted a high-risk group of PLHIV, involving 21,777 individuals. Two studies focused on liver disease patients, including one study on HCC and one study on other liver diseases, encompassing 190 patients. Additionally, three studies specifically

targeted refugees/immigrants, comprising a total of 2,815 individuals (Table 1).

In the study on HCV genotypes, three studies examined low-risk groups, which included the general population and blood donors, with a combined sample size of 363 subjects. Another three studies focused on high-risk groups such as PWID and PLHIV, involving 911 individuals. Two studies concentrated on patients with liver disease, encompassing a total of 552 patients. Furthermore, two studies specifically targeted refugees, with a total of 19 participants (Table 1).

Seroprevalence of HCV antibodies

Seroprevalence in the low-risk group

Low-risk populations were addressed in 9 studies and the total number of subjects screened was 606,498 of which 6,851 subjects were positive. Five studies focused on the general population, three on blood donors, and one on HBV vaccine non-responders. The seroprevalence ranged between 0 and 11.55% and the pooled seroprevalence of HCV was estimated at 2.18% (95%CI: 1.09-3.58). However, significant heterogeneity was found in this group (I^2=99.8%, p=0) (Fig~~ure~~ 2). A sensitivity analysis indicated that the prevalence of HCV antibodies increased to 2.41% (95% CI: 0.51%-5.4%) after excluding 3 unpublished reports~~papers (add citations)~~ (n~~N~~=72,680~~xxxx~~). However, this difference was not statistically significant (the result not shown) ~~(p??)~~.

Figure 2: Forest plot of the pooled HCV seroprevalence in Myanmar, using random effect

Legend: This figure shows the funnel plot of 33 studies and the pooled HCV seroprevalence in sub-groups: low-risk group, high-risk group, liver disease patients, and refugees/immigrants. The diamond block represents for pooled prevalence and the rectangular block represents prevalence for each study.

~~**Figure 2:** Forest plot of the pooled HCV seroprevalence in Myanmar, using random effect~~

Seroprevalence in the high-risk group

Individuals in the high-risk group were investigated in eleven studies: five on PWID, three on PLHIV, two on

Formatted: Font: 16 pt
Formatted: Font: 14 pt
Formatted: Not Highlight
Formatted: Not Highlight
Formatted: Not Highlight
Formatted: Not Highlight
Formatted: Not Highlight
Formatted: Not Highlight
Formatted: Font color: Black
Formatted: Font: Italic
Formatted: Font: 14 pt

patients who received blood transfusion, and one on hemodialysis patients. A total of 57,101 high-risk individuals were studied, and 7,930 were HCV ~~antibodies positive~~antibodies positive. Seroprevalence varied from 6.51% to 79.19%, and the pooled seroprevalence was 37.07% (95%CI: 22.85-52.49). Heterogeneity was also significant in this group (I^2=99.89%, p=0) (Fig~~ure~~ 2).

Seroprevalence in the liver disease patients

Formatted: Font: 14 pt

Liver disease patients were investigated in seven studies: four on hepatocellular carcinoma patients and three on other liver disease patients. Out of a total of 586 patients, 174 were HCV antibodies positive. Seroprevalence varied from 20.00% to 48.28%, and the pooled seroprevalence was 33.84% (95% CI: 25.29-42.93). Heterogeneity was also significant in this group (I^2=65.13%, p=0.0086) (Fig~~ure~~ 2).

Seroprevalence in the immigrants/refugees

Formatted: Font: 14 pt

Myanmar citizen immigrants/refugees who are living outside the country were investigated in six studies. Among a total of 21,218 individuals, 598 were HCV antibodies positive. Seroprevalence varied from 0 to 9.80% and the pooled seroprevalence was 2.52% (95%CI: 1.52-3.73). Heterogeneity was also significant in this group (I^2=83.24%, p=<0.0001) (Fig~~ure~~ 2).

Seroprevalence by study subjects

Formatted: Font: 14 pt

The highest HCV antibodies positive rate was found in PWID (58.06%, 95% CI: 56.94–59.18), individuals receiving blood transfusion (51.53%, 95% CI: 34.55–68.52), and liver disease patients (28.80%, 95% CI: 25.19-32.42). The seroprevalence of HCV among the general population was 2.82% (95% CI: 2.46–3.18, I^2 = 96.57%, $p < 0.0001$), and that among blood donors was 0.68% (95% CI: 0.66-0.70, I^2=99.92%, p=<0.00001) (Table 2).

Table 2: HCV seroprevalence in sub-groups

Categories	Number of studies	Total population	Pooled seroprevalence	95% CI	I^2 index (%)	Cochran Q test p-value
Study subjects						
General population	5	7,989	2.82	2.46-3.18	96.57	<0.00001
Blood donors	3	598,486	0.68	0.66-0.70	99.92	<0.00001
HBV vaccine non-responders	1	23	0.00	0.00-0.00	NA	NA
PWID	5	7,308	58.06	56.94-59.18	97.49	<0.00001
PLHIV	3	49,649	7.36	7.13-7.59	96.12	<0.00001
Patients who received blood transfusion	2	33	51.53	34.55-68.52	NA	NA
Haemodialysis patients	1	111	15.32	8.62-22.02	NA	NA
Liver disease patients*	7	586	28.80	25.19-32.42	63.87	0.01
Refugees/immigrants	6	21,218	2.72	2.50-2.94	79.70	0.0002
Data collection period[†]						
2000-2005	1	154,161	2.60	2.52-2.70	NA	NA
2006-2010	1	65,237	0.95	0.90-1.03	NA	NA

2011-2015	2	384,635	0.51	0.48-0.53	99.99	<0.00001

HBV, hepatitis B virus; *PWID*, people who inject drugs; *PLHIV*, people living with human immunodeficiency virus; *NA*, not applicable.

* Patients with liver diseases including hepatocellular carcinoma; [†] Analyses performed considering only low-risk groups (blood donors and general population).

Seroprevalence by the study period of data collection

Formatted: Font: 14 pt

Only low-risk groups were considered for HCV seroprevalence estimate by the study period to see the pure trend of its prevalence. One unpublished study was conducted between 2000-2005, resulting in a seroprevalence of 2.60% (95%CI: 2.52-2.70). The seroprevalence decreased to 0.95% (95%CI: 0.90-1.03, one study[31]) during 2006-2010 and to 0.51% (95%CI: 0.48-0.53, I^2=99.99%, p=<0.00001) during 2011-2015 (two studies including one unpublished study[10]) (Table 2). This decreasing trend was significant (p for difference=0.03).

HCV RNA prevalence

Formatted: Font: 16 pt

Eight studies[8, 22, 28, 30, 32, 35] measured HCV RNA, and the positive rate ranged from 0.37% in refugees to 25.45% in liver disease patients. The pooled prevalence of HCV RNA was estimated at 1.40% (95%CI: 0.08-3.71, I^2=0%, p=0.3390) in low-risk group, 5.25% (95%CI: 4.96-5.55) in high-risk group, 24.96% (95%CI: 18.91-31.51, I^2=0%, p=0.0115) in patients with liver diseases and 0.84% (95%CI: 0.19-1.84, I^2=73.65%, p=0.0225) in refugees/immigrants (Fig~~ure~~ 3).

Figure 3: Forest plot of the pooled HCV RNA prevalence in Myanmar, using random effect

Formatted: Indent: Left: 0", First line: 0"

Legend: This figure shows the funnel plot of 7 studies and the pooled HCV RNA prevalence in sub-groups: low-risk group, liver disease patients, and refugees/immigrants. The diamond block represents for pooled prevalence and the rectangular block represents prevalence for each study.

Formatted: Font: Italic

~~**Figure 3:** Forest plot of the pooled HCV RNA prevalence in Myanmar, using random effect~~

HCV genotypes

Formatted: Font: 16 pt

The genotyping of the HCV was performed in 10 studies[8, 18, 21, 23, 28, 32, 35, 40, 41, 46]. Overall, HCV genotype 3 was most predominant (48%) followed by genotypes 1 (28%), 6 (23%), 2 (1%), and 4 (0.27%). By sub-group analysis, genotype 3 is predominant in all sub-groups of low-risk, high-risk, liver disease patients and refugees/immigrants (43%, 48%, 52%, and 53% respectively). The HCV genotype distribution pattern was

similar~~ame~~ between low-risk groups and refugees/immigrants: genotype 3 (43% vs. 53%) followed by 6 (36% vs. 37%) and 1 (21% vs. 11%). The HCV genotype distribution was the similar~~ame~~ in high-risk groups and liver disease patients: genotype 3 (48% vs 52%) followed by 1 (31% vs. 28%) and 6 (21% vs. 18%). HCV genotype 2 was exclusively found in low-risk and liver disease patients (1% and 2% respectively) and only one case of genotype 4 was reported in a high-risk group (PLHIV) (Fig~~ure~~ 4). The analysis of HCV genotype distribution by region showed that genotype 6 is predominant in the northern part of Myanmar whilst genotype 3 is predominant in the west and southern parts of Myanmar (Fig~~ure~~ 5).

Figure 4: Distribution of HCV genotypes in Myanmar

Legend: This figure shows the HCV genotype distribution overall and by group of population in Myanmar. Each color represents each genotype: light blue for Genotype 1, orange for Genotype 2, dark blue for Genotype 3, and light green for Genotype 6.

Formatted: Font: Italic

Figure 5: HCV genotypes distribution by study place in Myanmar (N=1,294)

Legend: This figure shows the analysis of HCV genotype distribution by region in Myanmar. Each pie chart represents each study place. Each color inside the pie chart represents each genotype: light blue for Genotype 1, orange for Genotype 2, dark blue for Genotype 3, and light green for Genotype 6.

Formatted: Font: Italic

~~**Figure 4:** Distribution of HCV genotypes in Myanmar~~

~~**Figure 5:** Distribution of HCV genotypes by study place in Myanmar (N=1,294)~~

Publication bias

Formatted: Font: 16 pt

Using the graphical assessment of the funnel plot, the publication bias was found in HCV seroprevalence studies, but there was no publication bias in HCV RNA prevalence studies. In the Egger tests for funnel plot asymmetry, the p-values were 0.0001 and 0.8392 respectively (Fig~~ure~~ 6a and 6b).

~~**Figure 2: Forest plot of the pooled HCV seroprevalence in Myanmar, using random effect**~~

~~This figure shows the funnel plot of 33 studies and the pooled HCV seroprevalence in sub-groups: low-risk group, high-risk group, liver disease patients, and refugees/immigrants. The diamond block represents for pooled prevalence and the rectangular block represents prevalence for each study.~~

Figure 6: Funnel plot of HCV prevalence a) HCV antibodies and b) HCV RNA

Figures 6a and 6b show the forest plot for the assessment of publication bias among the recruited studies for estimating pooled HCV antibodies and HCV RNA prevalence. The dotted line represents the resultant pooled prevalence, and each black circle represents the prevalence of each study. The prevalence was plotted against the sample size.

~~**Figure 6:** Funnel plot of HCV prevalence a) HCV antibodies b) HCV RNA~~

Discussion

Formatted: Font: 18 pt

This systematic review revealed Myanmar has the intermediate endemicity of HCV seroprevalence among low-risk groups including the general population. The analysis of HCV seroprevalence by sub-groups offered a more comprehensive understanding of the disease burden within each group in Myanmar. ~~Analyzing HCV seroprevalence by sub-groups provided a broader understanding of the disease burden in each group in Myanmar.~~ The disease spectrum differs by the characteristic of each group, and it is encountered to consider adopting effective countermeasures against HCV in Myanmar.

This meta-analysis revealed publication bias in HCV seroprevalence studies but not in HCV RNA prevalence studies. The presence of publication bias indicates a likelihood of overestimating seroprevalence rates due to the selective publication of studies with higher rates. Therefore, the pooled prevalence was not determined as a whole,

and the participants were categorized into four groups: low-risk groups, high-risk groups, liver disease patients, and immigrants/refugees computed the pooled prevalence in each group using a random effect. This meta-analysis included the Burmese immigrants or refugees residing in other countries and it is believed that this group of the population reflects the disease burden of HCV among the general population in Myanmar.

HCV seroprevalence in low-risk groups and immigrants/refugees were 2.18% and 2.52% respectively. Despite the sensitivity analysis showing a slight increase in HCV antibody prevalence after ~~adjusting for~~ removing the unpublished reports in low-risk groups, the change was not statistically significant. However, caution should be~~,~~ exercised~~underscoring the need for~~ when~~cautious~~ interpret~~ationing of~~ing the pooled prevalence estimates. A significantly high level of heterogeneity was noticed in both groups (I^2: 99.8% vs. 83.24%). Considering the prevalence of 2.18% in the general population, 1.18 million individuals were exposed to HCV infection across the country as of 2023 Myanmar's population data [47]. In Myanmar, a nationwide study on the prevalence of HBV and HCV was conducted in 18 study sites in 2015 and reported that the prevalence of HBV and HCV was 6.5% and 2.7% respectively [10]. The pooled HCV seroprevalence among low-risk groups and immigrants/refugees in this meta-analysis is consistent with a 2015 nationwide study. The studies conducted from 2000 through 2017 among the abovementioned population groups likely do not reflect the current epidemiological situation of HCV among the general population in Myanmar.~~As the studies among the abovementioned groups of the population were conducted from 2000 through 2017, it is supposed not to reflect the current epidemiological situation of HCV among the general population in Myanmar.~~

The pooled HCV seroprevalence of 37.07% was found in high-risk groups which comprised of frequent blood recipients (Thalassemia etc.), hemodialysis patients, PWID, and PLHIV, all of whom were highly prone to HCV infection. A significant high level of heterogeneity was observed in this group with reported I^2 at 99.8%. The reported studies were conducted from 1996 through 2018. The high HCV seroprevalence rates among PWID, ranging from 40% to 79%, highlight the critical importance of implementing targeted interventions although harm reduction strategies that have been introduced since 2005-2006 in Myanmar. Moreover, the HIV/HCV coinfection rate ranged from 5% to 22.8% and the risk was high in the PWID population of Myanmar (Odds Ratio: 20.1, 95% CI: 13.7 to 29.5) [48, 49] which highlights the interplay between these two infections and the need for integrated prevention and treatment approaches in PWID.

The pooled HCV seroprevalence among liver disease patients was calculated to be 33.84% with a reported medium heterogeneity of 65.13%. All the studies on liver disease patients were conducted between 1991 and 1998 and there

Formatted: Not Highlight

Formatted: Not Highlight

Formatted: Not Highlight

Formatted: Not Highlight

Formatted: Not Highlight

Formatted: Not Highlight

Formatted: Highlight

was no report or studies after 2002. Most of the studies used convenient sampling methods for participant recruitment and the under or overestimation of HCV seroprevalence among this group cannot be ruled out. After two decades, the attribution of HBV and HCV etiology to liver disease including HCC might be changed and it needs to be evaluated again.

The decreasing trend of HCV seroprevalence among the low-risk group was found by a period of data collection. It can be explained by the installation of blood donor screening in 2000, the promotion of infection control including disposable syringe usage in health care facilities in the early 2000 and raising the awareness of viral hepatitis in 2011 with local and international non-governmental organizations' involvement in viral hepatitis screening and health education.

HCV RNA positive rate was supposed to be low in low-risk and immigrants/refugee groups (1.4% vs 0.84%) assuming that approximately 50% of them were naturally resolved and the number of viremic cases was estimated at 0.7 million individuals in Myanmar. The high HCV RNA-positive rate was found in liver disease patients (24.96%) but its attribution to hepatocellular carcinoma is unknown.

There were no incidence studies on HCV infection in Myanmar and only a few of the included prevalence studies were conducted mostly in an urban area with representative samples of the target population and there were no studies or reports on HCV disease burden after the 2015 nationwide study. Furthermore, the coronavirus disease 2019 (COVID-19) pandemic together with the unstable political situation in 2021 delayed or interrupted the healthcare system in Myanmar which is threatening the infectious diseases control, countermeasures, and the road to elimination of viral hepatitis in Myanmar. A well-structured basic epidemiological study to examine the disease burden of HCV in Myanmar is recommended.

In Myanmar, the predominant HCV genotype was 3 (48%) followed by 1 (28%) and 6 (23%) overall. This information is significant in guiding HCV treatment strategies, as genotypes can impact disease progress, treatment response, and duration. For instance, genotype 3 is associated with a higher risk of developing severe liver injuries, such as cirrhosis and hepatocellular carcinoma [50]. The obvious geographical difference in genotype distribution was found across the country. In the northern region of Myanmar, which shares a border with China's Yunnan province, a high prevalence of HCV genotype 6 was observed in the PWID population. Likewise, PWID in Yunnan province, HCV genotype 6 was found to be the predominant genotype. This finding suggests that there may be transmission of HCV among PWID populations occurring through the border regions between Myanmar and Yunnan province [51]. However, HCV genotype 3 was predominant in the southern part of Myanmar, and the

southern east of Myanmar is bordered by Thailand where genotype 3 is reportedly predominant [52]. HCV genotype 3 was also predominant in the western part of Myanmar, which shares borders with India and Bangladesh. These neighboring countries also have a high prevalence of genotype 3 [53, 54]. Understanding the genotype distribution and its geographical difference revealed the potential route of transmission from which effective prevention and control strategies should be developed. The genotypic diversity observed in Myanmar serves as a valuable contribution to the overall understanding of HCV genotypes in the Southeast Asia region.

This study has several limitations. The significant heterogeneity among the included studies may introduce variations in the estimated prevalence rates. This heterogeneity can be attributed to differences in sampling methods, individual characteristics of the study population, involvement of small sample sizes in some studies, and sampling biases in each recruited study so that the over or underestimation of the prevalence cannot be excluded. Most of the included studies did not provide the study setting clearly so that the urban and rural areas cannot be categorized, but most studies were assumed to be conducted in urban settings so the study in rural settings is limited in Myanmar and the actual disease burden among rural population is unknown. Moreover, the study on HCV among not only key populations such as men who have sex with men (MSM), female sex workers (FSW), and people in prisons but also the health care professionals (HCP) and pregnant women are still unknown.

In conclusion, this systematic review and meta-analysis indicated that Myanmar has intermediate endemicity of HCV seroprevalence in the low-risk group with a decreasing trend over the past 20 years due to measures such as blood donor screening, infection control practices, and increased awareness through education programs. However, the findings highlight the need for further epidemiological studies to understand the actual disease burden and implement effective countermeasures, including screening, linkage to care, and accessibility to treatment, to achieve the WHO's goal of HCV elimination by 2030.

List of abbreviations

HCV: Hepatitis C virus; DAA: Direct-acting antivirus; RNA: Ribonucleic acid; WHO: World Health Organization; PRISMA: Preferred Reporting Items for Systematic Reviews and Meta-Analysis; PROSPERO: International Prospective Register of Systematic Reviews; INPLASY: International Platform of Registered Systematic Review and Meta-analysis Protocols; CI: Confidence Interval; MMA: Myanmar Medical Association; MLF: Myanmar Liver Foundation; MSF: Medecins Sans Frontieres; NHCP: National Hepatitis Control Program; SVR: Sustained virological response; PLHIV: People living with human immunodeficiency virus; PWID: People who inject drugs;

HBV: Hepatitis B Virus HCC: Hepatocellular carcinoma; HIV: Human immunodeficiency virus; MSM: Men having sex with men; FSW: Female sex workers; HCP: Health care professional.

Declarations

Ethical approval and consent to participate

Not applicable.

Consent for publication

Not applicable.

~~Availability of data and materials~~

~~The dataset used and analysed in this study is available from the corresponding author upon reasonable request.~~

Competing interests

The authors declare no conflict of interest.

Role of fFunding sources

This work was supported by ~~Japan's Ministry of Health, Labour and Welfare (MHLW) Policy Research for Hepatitis Measures Program Grant Number JPMH22HC1001 and~~ Japan Society for the Promotion of Science (JSPS) Core-to-Core Program (Grant number: JPJSCCB20240009). ~~This work was supported by Japan's Ministry of Health, Labour and Welfare (MHLW) Policy Research for Hepatitis Measures Program Grant Number JPMH22HC1001.~~ The funders had no role in study design, data collection and analysis, decision to publish, or preparation of the manuscript.

Availability of data ~~and materials~~

Formatted: Font: 18 pt

Formatted: Font: 16 pt

Formatted: Font: 16 pt

Formatted: Font: 16 pt

Formatted: Font: 16 pt

Formatted: Font: 18 pt

Formatted: Font: 18 pt

Formatted: Highlight

Formatted: Font: 18 pt

The dataset used and analysed in this study is available from the corresponding author upon reasonable request.

Acknowledgment

We would send heartfelt gratitude to all the authors of the original papers which were used in this systematic review and meta-analysis. We would like to thank the personnel of the Department of Epidemiology, Infectious Disease Control, and Prevention, Graduate School of Biomedical and Health Sciences, Hiroshima University, for their valuable support.

Authors’ contributions

Formatted: Font: 18 pt

ZP, KK, and JT conceptualized the study. ZP, KK, and SO contributed to developing the study design and data acquisition. ZP, KK, SO, TA, and JT analysed and interpreted the data. ZP, KK, and SO drafted the manuscript. AS, UM, GA, CC, TA, and JT contributed to the intellectual content of the manuscript. All authors read and approved the final manuscript.

Acknowledgment

We would send heartfelt gratitude to all the authors of the original papers which were used in this systematic review and meta-analysis. We would like to thank the personnel of the Department of Epidemiology, Infectious Disease Control, and Prevention, Graduate School of Biomedical and Health Sciences, Hiroshima University, for their valuable support.

Formatted: Font: 18 pt

References

1. HCV Fact Sheet. World Health Organization (WHO). 2024 April 9. [Cited 2024 June 7]. Available from: https://www.who.int/news-room/fact-sheets/detail/hepatitis-c.

2. Global Hepatitis Report. World Health Organization (WHO); 2017.

3. Polaris Public Access, Regional Dashboard. [Cited 2023 June 28]. Available from https://cdafound.org/polaris-regions-compare/.

4. Yee WL, Bowring A, Draper B, O'Keefe D, Htay H, Myint KT, et al. Patients' access to and acceptance of community-based hepatitis C testing and treatment in Myanmar: A mixed-method study. PLOS Glob Public Health. 2023;3(6):e0000902. Epub 2023/06/16. doi: 10.1371/journal.pgph.0000902. PubMed PMID: 37327249; PubMed Central PMCID: PMCPMC10275420. following competing interests: Margaret Hellard has received investigator-initiated grant funding from Gilead Sciences and Abbvie for unrelated work. Alisa Pedrana has received investigator-initiated grant funding from Gilead Sciences, MSD and Abbvie and speaker fees from Gilead Sciences for unrelated work. Win Lei Yee has received a Gilead Sciences Fellowship for related work. Yi Yi Sein has received non-financial support from Mylan. There is no competing interest for all other authors.

5. Myanmar national simplified treatment guidelines for hepatitis C infection. Ministry of Health and Sports Myanmar. 2nd edn, 2019. [Cited 2024 June 7]. Available from: https://mohs.gov.mm/page/3212. - 2nd edn CJAfhm.

6. Draper BL, Pedrana A, Howell J, Yee WL, Htay H, Aung KS, et al. Decentralized, Community-Based Hepatitis C Point-of-Care Testing and Direct-Acting Antiviral Treatment for People Who Inject Drugs and the General Population in Myanmar: Protocol for a Feasibility Study. JMIR Res Protoc. 2020;9(7):e16863. Epub 2020/07/17. doi: 10.2196/16863. PubMed PMID: 32673260; PubMed Central PMCID: PMCPMC7388045.

7. Draper BL, Yee WL, Shilton S, Bowring A, Htay H, Nwe N, et al. Feasibility of decentralised, task-shifted hepatitis C testing and treatment services in urban Myanmar: implications for scale-up. BMJ Open. 2022;12(5):e059639. Epub 2022/05/04. doi: 10.1136/bmjopen-2021-059639. PubMed PMID: 35504640; PubMed Central PMCID: PMCPMC9066562.

8. Swe TM, Johnson DC, Mar HT, Thit P, Homan T, Chu CM, et al. Epidemiological characteristics and real-world treatment outcomes of hepatitis C among HIV/HCV co-infected patients in Myanmar: A prospective cohort study. Health Sci Rep. 2023;6(2):e1119. Epub 2023/02/24. doi: 10.1002/hsr2.1119. PubMed PMID: 36819986; PubMed Central PMCID: PMCPMC9938359.

9. Thu A, Thaw A, Oo KM, Lwin AA, Lwin O, Myint AZ, et al. Prevalence and associated risks of hepatitis B and C infections among Injecting Drug Users (IDU) attending the registered Drug Treatment Centers in Yangon, Mandalay, Myitkyina, Bamaw and Moekaung in 2007. Myanmar Health Sciences Research Journal. 2008; 20(3): 159-164.

10. Lwin AA, Aye KS, Htun MM, Kyaw YY, Zaw KK, Aung TT, et al. Sero-prevalence of Hepatitis B and C Viral Infections in Myanmar: National and Regional Survey in 2015. Myanmar Health Sciences Research Journal. 2017; 29(3): 167-175.

11. Spearman CW, Dusheiko GM, Hellard M, Sonderup M. Hepatitis C. Lancet. 2019;394(10207):1451-66. Epub 2019/10/22. doi: 10.1016/s0140-6736(19)32320-7. PubMed PMID: 31631857.

Formatted: Font: 18 pt

Formatted: Superscript

12. Andrade C. The Inconvenient Truth About Convenience and Purposive Samples. Indian J Psychol Med. 2021;43(1):86-8. Epub 2021/08/06. doi: 10.1177/0253717620977000. PubMed PMID: 34349313; PubMed Central PMCID: PMCPMC8295573.

13. Petruzziello A, Marigliano S, Loquercio G, Cacciapuoti C. Hepatitis C virus (HCV) genotypes distribution: an epidemiological up-date in Europe. Infect Agent Cancer. 2016;11:53. Epub 2016/10/19. doi: 10.1186/s13027-016-0099-0. PubMed PMID: 27752280; PubMed Central PMCID: PMCPMC5062817.

14. Page MJ, McKenzie JE, Bossuyt PM, Boutron I, Hoffmann TC, Mulrow CD, et al. The PRISMA 2020 statement: an updated guideline for reporting systematic reviews. Bmj. 2021;372:n71. Epub 2021/03/31. doi: 10.1136/bmj.n71. PubMed PMID: 33782057; PubMed Central PMCID: PMCPMC8005924. at http://www.icmje.org/conflicts-of-interest/ and declare: EL is head of research for the BMJ; MJP is an editorial board member for PLOS Medicine; ACT is an associate editor and MJP, TL, EMW, and DM are editorial board members for the Journal of Clinical Epidemiology; DM and LAS were editors in chief, LS, JMT, and ACT are associate editors, and JG is an editorial board member for Systematic Reviews. None of these authors were involved in the peer review process or decision to publish. TCH has received personal fees from Elsevier outside the submitted work. EMW has received personal fees from the American Journal for Public Health, for which he is the editor for systematic reviews. VW is editor in chief of the Campbell Collaboration, which produces systematic reviews, and co-convenor of the Campbell and Cochrane equity methods group. DM is chair of the EQUATOR Network, IB is adjunct director of the French EQUATOR Centre and TCH is co-director of the Australasian EQUATOR Centre, which advocates for the use of reporting guidelines to improve the quality of reporting in research articles. JMT received salary from Evidence Partners, creator of DistillerSR software for systematic reviews; Evidence Partners was not involved in the design or outcomes of the statement, and the views expressed solely represent those of the author.

15. Munn Z, Moola S, Lisy K, Riitano D, Tufanaru C. Methodological guidance for systematic reviews of observational epidemiological studies reporting prevalence and cumulative incidence data. Int J Evid Based Healthc. 2015;13(3):147-53. Epub 2015/09/01. doi: 10.1097/xeb.0000000000000054. PubMed PMID: 26317388.

16. Higgins JP, Thompson SG, Deeks JJ, Altman DG. Measuring inconsistency in meta-analyses. Bmj. 2003;327(7414):557-60. Epub 2003/09/06. doi: 10.1136/bmj.327.7414.557. PubMed PMID: 12958120; PubMed Central PMCID: PMCPMC192859.

17. Hunter JP, Saratzis A, Sutton AJ, Boucher RH, Sayers RD, Bown MJ. In meta-analyses of proportion studies, funnel plots were found to be an inaccurate method of assessing publication bias. J Clin Epidemiol. 2014;67(8):897-903. Epub 2014/05/06. doi: 10.1016/j.jclinepi.2014.03.003. PubMed PMID: 24794697.

18. Mellor J, Walsh EA, Prescott LE, Jarvis LM, Davidson F, Yap PL, et al. Survey of type 6 group variants of hepatitis C virus in Southeast Asia by using a core-based genotyping assay. J Clin Microbiol. 1996;34(2):417-23. Epub 1996/02/01. doi: 10.1128/jcm.34.2.417-423.1996. PubMed PMID: 8789027; PubMed Central PMCID: PMCPMC228809.

19. Kyi KP, Aye M, Oo KM, Htun MM, Oo SS, Lwin KO, et al. Prevalence of Hepatitis C in Healthy Population and Patients with Liver Ailments in Myanmar. Regional Health Forum. 2002; 6(1): 1-6.

20. Taketa K, Okada S, Win N, Hlaing NK, Wind KM. Evaluation of tumor markers for the detection of hepatocellular carcinoma in Yangon General Hospital, Myanmar. Acta Med Okayama. 2002;56(6):317-20. Epub 2003/04/11. doi: 10.18926/amo/31689. PubMed PMID: 12685861.

21. Lwin AA, Shinji T, Khin M, Win N, Obika M, Okada S, et al. Hepatitis C virus genotype distribution in Myanmar: Predominance of genotype 6 and existence of new genotype 6 subtype. HEPATOLOGY RESEARCH. 2007;37(5):337-45. doi: 10.1111/j.1872-034X.2007.00053.x. PubMed PMID: WOS:000245693200005.

22. Htun MM, Kyi KP, Oo KM, Khin M, Tun WM, Oo SS, et al. Presence of hepatitis C virus RNA by nested polymerase chain reaction (RT-PCR) in plasma-derived hepatitis B vaccine non-responders. Myanmar Health Sciences Research Journal. 2010; 22(2): 101-104.

23. Naing C, Sitt T, Aung AT, Aung K. Sustained Virologic Response to a Dual Peginterferon alfa-2a and Ribavirin in Treating Chronic hepatitis C Infection: A Retrospective Cohort Study. Medicine (Baltimore). 2015;94(30):e1234. Epub 2015/07/30. doi: 10.1097/md.0000000000001234. PubMed PMID: 26222859; PubMed Central PMCID: PMCPMC4554122.

24. Nyunt KT, Htoo KPP, Hnin MT, Kyaw PM, Mon EK, Thu ISL, et al. Hepatitis B and Hepatitis C infection among teaching staff and non-teaching from University of Medical Technology, Mandalay. Myanmar Medical Journal. 2019; 61(4): 35-43.

25. Kyi KP, Win KM, Aye M, Htwe YY, Oo KM, Aung T, et al. Prevalence of hepatitis B and C infections in hepatocellular carcinoma cases in Myanmar. Myanmar Health Sciences Research Journal. 1998; 10(1): 1-5.

26. Okada S, Taketa K, Ishikawa T, Koji T, Swe T, Win N, et al. High prevalence of hepatitis C in patients with thalassemia and patients with liver diseases in Myanmar (Burma). ACTA MEDICA OKAYAMA. 2000;54(3):137-8. PubMed PMID: WOS:000087965700007.

27. Win N, Okada S, Win KM, Taketa K, Akiyama T, Aye KS, et al. Hemoglobinopathies, Iron Overload and Chronic Viral Hepatitis in Patients with Hepatocellular Carcinoma in Myanmar. Asian Pac J Cancer Prev. 2000;1(2):127-30. Epub 2003/04/30. PubMed PMID: 12718679.

28. Nakai K, Win KM, Oo SS, Arakawa Y, Abe K. Molecular characteristic-based epidemiology of hepatitis B, C, and E viruses and GB virus C/hepatitis G virus in Myanmar. JOURNAL OF CLINICAL MICROBIOLOGY. 2001;39(4):1536-9. doi: 10.1128/JCM.39.4.1536-1539.2001. PubMed PMID: WOS:000167946500055.

29. Denburg A, Rashid M, Brophy J, Curtis T, Malloy P, Audley J, et al. Initial health screening results for Karen refugees: a retrospective review. Can Commun Dis Rep. 2007;33(13):16-22. Epub 2007/12/29. PubMed PMID: 18161207.

30. Chaves NJ, Gibney KB, Leder K, O'Brien DP, Marshall C, Biggs BA. Screening practices for infectious diseases among Burmese refugees in Australia. Emerg Infect Dis. 2009;15(11):1769-72. Epub 2009/11/07. doi: 10.3201/eid1511.090777. PubMed PMID: 19891864; PubMed Central PMCID: PMCPMC2857247.

31. Khin M, Oo SS, Oo KM, Shimono K, Koide N, Okada S. Prevalence and Factors Associated with Hepatitis C Virus Infection among Myanmar Blood Donors. ACTA MEDICA OKAYAMA. 2010;64(5):317-21. PubMed PMID: WOS:000283563300007.

32. Akkarathamrongsin S, Praianantathavorn K, Hacharoen N, Theamboonlers A, Tangkijvanich P, Poovorawan Y. Seroprevalence and Genotype of Hepatitis C Virus among Immigrant Workers from Cambodia and Myanmar in Thailand. INTERVIROLOGY. 2011;54(1):10-6. doi: 10.1159/000318884. PubMed PMID: WOS:000282170300002.

33. Zhou YH, Liu FL, Yao ZH, Duo L, Li H, Sun Y, et al. Comparison of HIV-, HBV-, HCV- and Co-Infection Prevalence between Chinese and Burmese Intravenous Drug Users of the China-Myanmar Border Region. PLOS ONE. 2011;6(1). doi: 10.1371/journal.pone.0016349. PubMed PMID: WOS:000286522300038.

34. Paxton GA, Sangster KJ, Maxwell EL, McBride CR, Drewe RH. Post-arrival health screening in Karen refugees in Australia. PLoS One. 2012;7(5):e38194. Epub 2012/06/14. doi: 10.1371/journal.pone.0038194. PubMed PMID: 22693599; PubMed Central PMCID: PMCPMC3364970.

35. Mixson-Hayden T, Lee D, Ganova-Raeva L, Drobeniuc J, Stauffer WM, Teshale E, et al. Hepatitis B virus and hepatitis C virus infections in United States-bound refugees from Asia and Africa. Am J Trop Med Hyg. 2014;90(6):1014-20. Epub 2014/04/16. doi: 10.4269/ajtmh.14-0068. PubMed PMID: 24732462; PubMed Central PMCID: PMCPMC4047722.

36. Lwin AA, Htwe KM, Mon NCN, Maw KT, Hlaing MM, Thwin KT, et al. Hepatitis B and C Viral Infections among Hemodialysis Patients Attending 500 Bedded Yangon Specialty Hospital, Myanmar. Myanmar Health Sciences Research Journal. 2018; 30 (3): 213-217.

37. Aye NS, Oo MM, Harries AD, Mon MM, Hone S, Oo HN, et al. HIV, HBV and HCV in people who inject drugs and are placed on methadone maintenance therapy, Yangon, Myanmar. PUBLIC HEALTH ACTION. 2018;8(4):202-10. doi: 10.5588/pha.18.0050. PubMed PMID: WOS:000457790500010.

38. Ngo CC, Maidment C, Atkins L, Eagar S, Smith MM. Blood screen findings in a 2-year cohort of newly arrived refugees to Sydney, Australia. Public Health Res Pract. 2018;28(1). Epub 2018/03/28. doi: 10.17061/phrp2811804. PubMed PMID: 29582037.

39. Juon HS, Ha E, Kim F, Trang A, Pan J, Blanchard J. Prevalence of Viral Hepatitis in Foreign-Born Populations in the Baltimore-Washington Metropolitan Area, 2009-2015. JOURNAL OF COMMUNITY HEALTH. 2019;44(2):203-7. doi: 10.1007/s10900-018-0573-2. PubMed PMID: WOS:000460944900002.

40. Ye M, Chen X, Wang Y, Duo L, Zhang C, Zheng YT. Identification of a New HCV Subtype 6xg Among Injection Drug Users in Kachin, Myanmar. Front Microbiol. 2019;10:814. Epub 2019/05/07. doi: 10.3389/fmicb.2019.00814. PubMed PMID: 31057519; PubMed Central PMCID: PMCPMC6482298.

41. Chen M, Ma Y, Chen H, Dai J, Luo H, Jia M, et al. Complete genome sequencing and evolutionary analysis of HCV subtype 6xg from IDUs in Yunnan, China. PLoS One. 2019;14(5):e0217010. Epub 2019/05/17. doi: 10.1371/journal.pone.0217010. PubMed PMID: 31095618; PubMed Central PMCID: PMCPMC6522032.

42. Myanmar Integrated Biological and Behavioural Surveillance Survey and Population Size Estimates among People Who Inject Drugs (PWID) 2017-2018. National AIDS Programme (NAP), Myanmar Ministry of Health and Sports, Myanmar; 2019.

43. Win TS, Maw SY, Kyaw YY, Maw YY. Serodetection of Hepatitis C Virus Infection among HIV Patients in Mingalardon Specialist Hospital. Myanmar Health Sciences Research Journal, 2020; 32(1):19-24.

44. Kyaw NTT, Satyanarayana S, Harries AD, Kumar AMV, Kyaw KWY, Phyo KH, et al. Increased All-cause Mortality in People With HIV and Comorbidities: Hepatitis B and C Virus Seropositivity and Hyperglycemia in Myanmar, 2005-2017. Open Forum Infect Dis. 2023;10(1):ofac672. Epub 2023/01/13. doi: 10.1093/ofid/ofac672. PubMed PMID: 36632421; PubMed Central PMCID: PMCPMC9830546.

45. Urban K, Payton C, Mamo B, Volkman H, Giorgio K, Kennedy L, et al. Hepatitis C Screening and Antibody Prevalence Among Newly Arrived Refugees to the United States, 2010-2017. J Immigr Minor

Health. 2023:1-8. Epub 2023/03/31. doi: 10.1007/s10903-023-01471-8. PubMed PMID: 36995524; PubMed Central PMCID: PMCPMC10062256.

46. Shinji T, Kyaw YY, Gokan K, Tanaka Y, Ochi K, Kusano N, et al. Analysis of HCV genotypes from blood donors shows three new HCV type 6 subgroups exist in Myanmar. ACTA MEDICA OKAYAMA. 2004;58(3):135-42. PubMed PMID: WOS:000222273300004.

47. Worldometers. Myanmar Population. 2023. [Cited 2023 June 28]. Available from: https://www.worldometers.info/world-population/myanmar-population/.

48. Ousley J, Nesbitt R, Kyaw NTT, Bermudez E, Soe KP, Anicete R, et al. Increased hepatitis C virus co-infection and injection drug use in HIV-infected fishermen in Myanmar. BMC INFECTIOUS DISEASES. 2018;18. doi: 10.1186/s12879-018-3558-y. PubMed PMID: WOS:000453239800001.

49. Zaw SK, Tun ST, Thida A, Aung TK, Maung W, Shwe M, et al. Prevalence of hepatitis C and B virus among patients infected with HIV: a cross-sectional analysis of a large HIV care programme in Myanmar. Trop Doct. 2013;43(3):113-5. Epub 2013/06/27. doi: 10.1177/0049475513493416. PubMed PMID: 23800421.

50. Gondeau C, Pageaux GP, Larrey D. Hepatitis C virus infection: Are there still specific problems with genotype 3? World J Gastroenterol. 2015;21(42):12101-13. Epub 2015/11/18. doi: 10.3748/wjg.v21.i42.12101. PubMed PMID: 26576095; PubMed Central PMCID: PMCPMC4641128.

51. Zhang Z, Yao Y, Wu W, Feng R, Wu Z, Cun W, et al. Hepatitis C virus genotype diversity among intravenous drug users in Yunnan Province, Southwestern China. PLoS One. 2013;8(12):e82598. Epub 2013/12/21. doi: 10.1371/journal.pone.0082598. PubMed PMID: 24358211; PubMed Central PMCID: PMCPMC3866230.

52. Wasitthankasem R, Vongpunsawad S, Siripon N, Suya C, Chulothok P, Chaiear K, et al. Genotypic Distribution of Hepatitis C Virus in Thailand and Southeast Asia. PLOS ONE. 2015;10(5). doi: 10.1371/journal.pone.0126764. PubMed PMID: WOS:000354542500147.

53. Das BR, Kundu B, Khandapkar R, Sahni S. Geographical distribution of hepatitis C virus genotypes in India. Indian J Pathol Microbiol. 2002;45(3):323-8. Epub 2003/06/06. PubMed PMID: 12785176.

54. Islam MS, Miah MR, Roy PK, Rahman O, Siddique AB, Chowdhury J, et al. Genotypes of hepatitis C virus infection in Bangladeshi population. Mymensingh Med J. 2015;24(1):143-51. Epub 2015/03/03. PubMed PMID: 25725681.

~~Figure Legends~~

Formatted: Font: 18 pt

~~Figure 1: PRISMA showing a selection of articles for meta-analysis of HCV in Myanmar~~

This figure shows the selection process of the articles for the estimation of HCV prevalence in Myanmar. Each arrow indicates the step-by-step process for recruiting the articles and the detailed reason for the exclusion of articles were described with their respective exact number. This figure was built following the guideline for 2020 Preferred Reporting Items for Systematic Reviews and Meta-Analysis (PRISMA).

Figure 2: Forest plot of the pooled HCV seroprevalence in Myanmar, using random effect

This figure shows the funnel plot of 33 studies and the pooled HCV seroprevalence in sub-groups: low-risk group, high-risk group, liver disease patients, and refugees/immigrants. The diamond block represents for pooled prevalence and the rectangular block represents prevalence for each study.

Figure 3: Forest plot of the pooled HCV RNA prevalence in Myanmar, using random effect

This figure shows the funnel plot of 7 studies and the pooled HCV RNA prevalence in sub-groups: low-risk group, liver disease patients, and refugees/immigrants. The diamond block represents for pooled prevalence and the rectangular block represents prevalence for each study.

Figure 4: Distribution of HCV genotypes in Myanmar

This figure shows the HCV genotype distribution overall and by group of population in Myanmar. Each color represents each genotype: light blue for Genotype 1, orange for Genotype 2, dark blue for Genotype 3, and light green for Genotype 6.

Figure 5: HCV genotypes distribution by study place in Myanmar (N=1,294)

This figure shows the analysis of HCV genotype distribution by region in Myanmar. Each pie chart represents each study place. Each color inside the pie chart represents each genotype: light

~~blue for Genotype 1, orange for Genotype 2, dark blue for Genotype 3, and light green for Genotype 6.~~

~~Figure 6: Funnel plot of HCV prevalence a) HCV antibodies and b) HCV RNA~~

~~Figures 6a and 6b show the forest plot for the assessment of publication bias among the recruited studies for estimating pooled HCV antibodies and HCV RNA prevalence. The dotted line represents the resultant pooled prevalence, and each black circle represents the prevalence of each study. The prevalence was plotted against the sample size.~~

Supporting information ~~captions~~

S1 Appendix. PRISMA checklist

S2~~1~~ Table. Database search

S3~~2~~ Table. Quality appraisal of included studies using the Joanna Briggs Institute checklist for prevalence studies

S4 Checklist. Inclusivity in global research questionnaire

Response to Reviewer Comments

We wish to express our appreciation to the editor, and reviewers for their additional comments on our paper. We revised our paper based on the editor and reviewer's comment as below.

Response to Reviewer-1

Comment-1: The study was not registered in a prospective systematic review register such as PROSPERO. Have authors confirmed that there are no similar ongoing studies? If so, it should be clearly stated.

Response-1: Thank you so much for your comment. We acknowledge that we did not register the protocol in a systematic review registry. But, before the review, we searched databases such as PubMed, Web of Science, and Scopus for published studies on this topic and found none in PROSPERO, INPLASY, etc. We have clarified this in the manuscript (Page 4, Line 94-98) as below.

"Though we did not prospectively register our systematic review in the registry database such as International Prospective Register of Systematic Reviews (PROSPERO), International Platform of Registered Systematic Review and Meta-analysis Protocols (INPLASY) etc, there was no ongoing similar studies in those databases. Before the review, we searched databases such as PubMed, Web of Science, and Scopus for published studies on this topic and found none."

Comment-2: Authors should indicate which are 'studies' and 'reports' from the cited references in Table 1.

Response-2: We appreciate very much for your valuable comments. We added the symbol "^ in the studies issued from the same report" in the Table 1 and mentioned in the footnote.

Comment-3. Authors should consider performing a sensitivity analysis excluding unpublish data in groups where they occur.

Response-3: Thank you for your comment and important suggestion. We performed a sensitivity analysis by excluding unpublished data. All unpublished data were in low-risk groups only. In the initial analysis, the prevalence of HCV in low-risk groups was 2.18% [1.09% - 3.58%]. After excluding unpublished data, the prevalence increased to 2.41% [0.51%; 5.40%] in the low-risk group. This difference is not statistically significant (please see below). We have included the results and discussion of the sensitivity analysis in the manuscript, but we decided not to include the calculation result figure.

(Result: Page 12, Line 218-220)
"A sensitivity analysis indicated that the prevalence of HCV antibodies increased to 2.41% (95% CI: 0.51%-5.4%) after excluding 3 unpublished reports (n=72,680). However, this difference was not statistically significant (the result not shown)."

(Discussion: Page 17, Line 322-325)
"Despite the sensitivity analysis showing a slight increase in HCV antibody prevalence after removing the unpublished reports in low-risk groups, the change was not statistically significant. However, caution should be exercised when interpreting the pooled prevalence estimates."

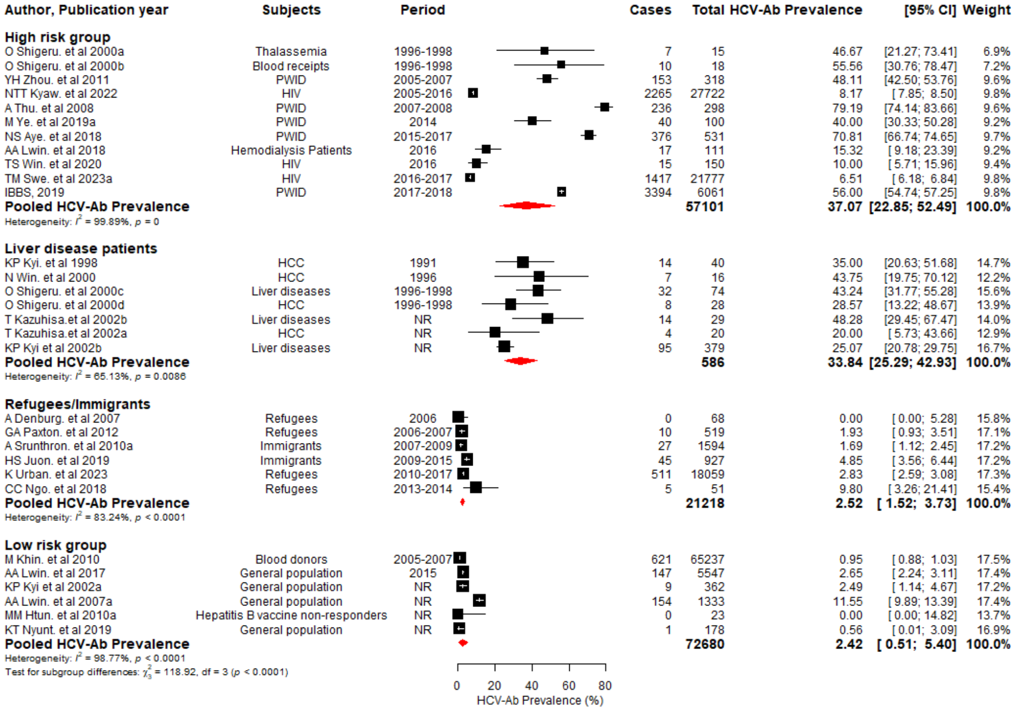

Comment-4: Line 272: How are the genotype distribution 'same' based on the proportion values provided in the sentence? Maybe 'similar'? Please clarify.

Response-4: Thank you so much for your clarification. It was our mistake, and we corrected as "similar". We have revised in the result section accordingly.

Response to Reviewer-2

Comment-1: The abstract should be rewritten stating the background, objective of the study. In the Methods, how data was extracted, and the persons involved. Also, the conclusion should reflect the answer to the main objective of the work.

Response-1: Thank you very much for your comments, we revised the abstract according to your suggestion. As we had provided the information of person involved in the main manuscript, please let us not include in the abstract.

Comment-2: In the Background section; The first paragraph should include what is HCV, before giving the prevalence. Also, Paragraph 2 is not necessary. Paragraph 3 and 4 should be rewritten, including more references, and stating clearly the importance of the work before stating the objectives.

Response-2: We appreciate very much for your valuable comments. Based on your suggestions, we deleted paragraph 2, added more references supporting the facts in background and clarified and justified the study aims based on our research title respectively.

Comment-3: In the Material and methods section: All the sentences starting with To, should be rephrased. I have highlighted in the manuscript.

Response-3: Thank you very much for your intensive review. Based on your suggestion, we revised all the statements started with "To" appropriately. We appreciate very much for your valuable comments.

Comment-4: In the results section; I have highlighted in the manuscript, Some sentences should be rephrased, starting appropriately.

Response-4: Thank you very much for your valuable suggestion, we have rephrased them accordingly.

Comment-5: In the Discussion section, Same comments as the results section.

Response-5: Thank you very much for your insightful suggestion, we rephrased and revised the statement as you commented details in the manuscript.

Comment-6: Conclusion is Ok.

Response-6: Thank you very much for your comment, we appreciate.

Comment-7: References: All the references should be rearranged and harmonised.

Response-7: Thank you very much for your comment and all the references were also harmonized according to the journal's requirement.

Response to the editorial office

Comment-1. Please ensure that your manuscript meets PLOS ONE's style requirements, including those for file naming. The PLOS ONE style templates can be found at https://journals.plos.org/plosone/s/file?id=wjVg/PLOSOne_formatting_sample_main_body.pdf and https://journals.plos.org/plosone/s/file?id=ba62/PLOSOne_formatting_sample_title_authors_affiliations.pdf

Response-1: Thank you very much for your comments, we formatted the manuscript based on the instruction.

Comment-2. We suggest you thoroughly copyedit your manuscript for language usage, spelling, and grammar. If you do not know anyone who can help you do this, you may wish to consider employing a professional scientific editing service.
The American Journal Experts (AJE) (https://www.aje.com/) is one such service that has extensive experience helping authors meet PLOS guidelines and can provide language editing, translation, manuscript formatting, and figure formatting to ensure your manuscript meets our submission guidelines. Please note that having the manuscript copyedited by AJE or any other editing services does not guarantee selection for peer review or acceptance for publication.
Upon resubmission, please provide the following:
The name of the colleague or the details of the professional service that edited your manuscript
*A copy of your manuscript showing your changes by either highlighting them or using track changes (uploaded as a *supporting information* file)*
*A clean copy of the edited manuscript (uploaded as the new *manuscript* file)*

Response-2: Thank you very much for your comments, ZP and KK, the first and coauthor of this manuscript did the intensive edition, and we provided the revised version in both clean and with tract change.

Comment-3. Please include a complete copy of PLOS' questionnaire on inclusivity in global research in your revised manuscript. Our policy for research in this area aims to improve transparency in the reporting of research performed outside of researchers' own country or community. The policy applies to researchers who have travelled to a different country to conduct research, research with Indigenous populations or their lands, and research on cultural artefacts. The questionnaire can also be requested at the journal's discretion for any other submissions, even if these conditions are not met. Please find more information on the policy and a link to download a blank copy of the questionnaire here: https://journals.plos.org/plosone/s/best-practices-in-research-reporting. Please upload a completed version of your questionnaire as Supporting Information when you resubmit your manuscript.

Response-3: Thank you very much for your comments, we provided the checklist for the Inclusivity in global research questionnaire as Supporting Information and mentioned in the method section with sub-heading (Page 6, Line 150-152).

"Inclusivity in global research: Additional information regarding the ethical, cultural, and scientific considerations specific to inclusivity in global research is included in the Supporting Information (S4 Checklist)."

Comment-4. Thank you for stating the following financial disclosure:
"This work was supported by Japan's Ministry of Health, Labour and Welfare (MHLW) Policy Research for Hepatitis Measures Program Grant Number JPMH22HC1001."
Please state what role the funders took in the study. If the funders had no role, please state: "The funders had no role in study design, data collection and analysis, decision to publish, or preparation of the manuscript."
If this statement is not correct you must amend it as needed.
Please include this amended Role of Funder statement in your cover letter; we will change the online submission form on your behalf.

Response-4: Thank you very much for your comments, we added this funder role statement in the manuscript and in the cover letter. Using this opportunity, we would like to change the funding source as "This work was supported by Japan Society for the Promotion of Science (JSPS) Core-to-Core Program (Grant number: JPJSCCB20240009). The funders had no role in study design, data collection and analysis, decision to publish, or preparation of the manuscript".

Comment-5. We note that Figure 5 in your submission contain [map/satellite] images which may be copyrighted. All PLOS content is published under the Creative Commons Attribution License (CC BY 4.0), which means that the manuscript, images, and Supporting Information files will be freely available online, and any third party is permitted to access, download, copy, distribute, and use these materials in any way, even commercially, with proper attribution. For these reasons, we cannot publish previously copyrighted maps or satellite images created using proprietary data, such as Google software (Google Maps, Street View, and Earth). For more information, see our copyright guidelines: http://journals.plos.org/plosone/s/licenses-and-copyright.

We require you to either (1) present written permission from the copyright holder to publish these figures specifically under the CC BY 4.0 license, or (2) remove the figures from your submission:
a. You may seek permission from the original copyright holder of Figure 5 to publish the content specifically under the CC BY 4.0 license.
We recommend that you contact the original copyright holder with the Content Permission Form (http://journals.plos.org/plosone/s/file?id=7c09/content-permission-form.pdf) and the following text:
"I request permission for the open-access journal PLOS ONE to publish XXX under the Creative Commons Attribution License (CCAL) CC BY 4.0 (http://creativecommons.org/licenses/by/4.0/). Please be aware that this license allows unrestricted use and distribution, even commercially, by third parties. Please reply and provide explicit written permission to publish XXX under a CC BY license and complete the attached form."
Please upload the completed Content Permission Form or other proof of granted permissions as an "Other" file with your submission.
In the figure caption of the copyrighted figure, please include the following text: "Reprinted from [ref] under a CC BY license, with permission from [name of publisher], original copyright [original copyright year]."
b. If you are unable to obtain permission from the original copyright holder to publish these figures under the CC BY 4.0 license or if the copyright holder's requirements are incompatible with the CC BY 4.0 license, please either i) remove the figure or ii) supply a replacement figure that complies with the CC BY 4.0 license. Please check copyright information on all replacement figures and update the figure caption with source information. If applicable, please specify in the figure caption text when a figure is similar but not identical to the original image and is therefore for illustrative purposes only.
The following resources for replacing copyrighted map figures may be helpful:
USGS National Map Viewer (public domain): http://viewer.nationalmap.gov/viewer/
The Gateway to Astronaut Photography of Earth (public domain): http://eol.jsc.nasa.gov/sseop/clickmap/
Maps at the CIA (public domain): https://www.cia.gov/library/publications/the-world-factbook/index.html and https://www.cia.gov/library/publications/cia-maps-publications/index.html
NASA Earth Observatory (public domain): http://earthobservatory.nasa.gov/
Landsat: http://landsat.visibleearth.nasa.gov/
USGS EROS (Earth Resources Observatory and Science (EROS) Center) (public domain): http://eros.usgs.gov/#
Natural Earth (public domain): http://www.naturalearthdata.com/

Response-5: Thank you very much for your comments. In fact, we created the map by ourselves using the free online based creation at http://n.freemap.jp. Therefore, no copyright issue to use the map from that abovementioned free webpage. But, for the printing of the colored map what we created, the copyright belongs to us.
