# Supplementary material for: Intermediate hepatitis C virus (HCV) endemicity and its genotype distribution in Myanmar: A systematic review and meta-analysis
Source: PLoS One. 2024 Sep 19;19(9):e0307872. doi: 10.1371/journal.pone.0307872 (PMC11412534; doi:10.1371/journal.pone.0307872)
Supplement: Attachment — Submitted filename: Response to Reviewers Final.pdf [file pone.0307872.s008.pdf]

Response to Reviewers' Comments

We wish to express our appreciation to the editor, and reviewer for their additional comments on our paper. We revised our paper based on the editor and reviewer's comment as below.

Response to Reviewer-2

Comment-1: The abstract should be segmented with the different headings: Background, Material and Methods, Results and conclusion.

Response-1: Thank you very much for your comments, we added subheading in the abstract.

Comment-2: The keywords should be arranged alphabetically.

Response-2: We appreciate very much for your comments. Based on your suggestions, we rearranged them.

Comment-3: The references should be checked, completed and harmonised following the journal guidelines.

Response-3: Thank you very much for your intensive review. Based on your suggestion, we checked all the references and revised reference number 1 to 5 and 47 based on the journal guidelines.

Response to the Editorial office

Comment-1. Please review your reference list to ensure that it is complete and correct. If you have cited papers that have been retracted, please include the rationale for doing so in the manuscript text, or remove these references and replace them with relevant current references. Any changes to the reference list should be mentioned in the rebuttal letter that accompanies your revised manuscript. If you need to cite a retracted article, indicate the article's retracted status in the References list and also include a citation and full reference for the retraction notice.

Response-1: Thank you for your detailed review. We have carefully examined our reference list and confirm that our manuscript does not cite any retracted papers. Based on your suggestions, we have checked all the references and revised the reference no. 1-5, 9, 19, 22, 36, and 47 according to the journal guidelines. Specifically, reference no. 9, 19, 22, and 36 were in an online article format, and we have provided the corresponding URL link in all the revised references.
