# Supplementary material for: Intermediate hepatitis C virus (HCV) endemicity and its genotype distribution in Myanmar: A systematic review and meta-analysis
Source: PLoS One. 2024 Sep 19;19(9):e0307872. doi: 10.1371/journal.pone.0307872 (PMC11412534; doi:10.1371/journal.pone.0307872)
Supplement: Peer review history

# Peer review: Decision Letter 0

**DOI:** 10.1371/journal.pone.0307872.r001

**Authors:** Yury E Khudyakov

2 Jun 2024

PONE-D-24-10345Intermediate hepatitis C virus (HCV) endemicity and its genotype distribution in Myanmar: A systematic review and meta-analysisPLOS ONE

Dear Dr. Tanaka,

Thank you for submitting your manuscript to PLOS ONE. After careful consideration, we feel that it has merit but does not fully meet PLOS ONE’s publication criteria as it currently stands. Therefore, we invite you to submit a revised version of the manuscript that addresses the points raised during the review process.

==============================

Your manuscript was reviewed by two experts in the field. Both identified important problems in your submission. Please review the attached comments and provide point-by-point responses.

==============================

Please submit your revised manuscript by Jul 17 2024 11:59PM. If you will need more time than this to complete your revisions, please reply to this message or contact the journal office at plosone@plos.org. When you're ready to submit your revision, log on to https://www.editorialmanager.com/pone/ and select the 'Submissions Needing Revision' folder to locate your manuscript file.

Please include the following items when submitting your revised manuscript:

- A rebuttal letter that responds to each point raised by the academic editor and reviewer(s). You should upload this letter as a separate file labeled 'Response to Reviewers'.
- A marked-up copy of your manuscript that highlights changes made to the original version. You should upload this as a separate file labeled 'Revised Manuscript with Track Changes'.
- An unmarked version of your revised paper without tracked changes. You should upload this as a separate file labeled 'Manuscript'.

If you would like to make changes to your financial disclosure, please include your updated statement in your cover letter. Guidelines for resubmitting your figure files are available below the reviewer comments at the end of this letter.

If applicable, we recommend that you deposit your laboratory protocols in protocols.io to enhance the reproducibility of your results. Protocols.io assigns your protocol its own identifier (DOI) so that it can be cited independently in the future. For instructions see: https://journals.plos.org/plosone/s/submission-guidelines#loc-laboratory-protocols. Additionally, PLOS ONE offers an option for publishing peer-reviewed Lab Protocol articles, which describe protocols hosted on protocols.io. Read more information on sharing protocols at https://plos.org/protocols?utm_medium=editorial-email&utm_source=authorletters&utm_campaign=protocols.

We look forward to receiving your revised manuscript.

Kind regards,

Yury E Khudyakov, PhD

Academic Editor

PLOS ONE

Journal requirements:

When submitting your revision, we need you to address these additional requirements.

1. Please ensure that your manuscript meets PLOS ONE's style requirements, including those for file naming. The PLOS ONE style templates can be found at

https://journals.plos.org/plosone/s/file?id=wjVg/PLOSOne_formatting_sample_main_body.pdf and

https://journals.plos.org/plosone/s/file?id=ba62/PLOSOne_formatting_sample_title_authors_affiliations.pdf

2. We suggest you thoroughly copyedit your manuscript for language usage, spelling, and grammar. If you do not know anyone who can help you do this, you may wish to consider employing a professional scientific editing service.

The American Journal Experts (AJE) (https://www.aje.com/) is one such service that has extensive experience helping authors meet PLOS guidelines and can provide language editing, translation, manuscript formatting, and figure formatting to ensure your manuscript meets our submission guidelines. Please note that having the manuscript copyedited by AJE or any other editing services does not guarantee selection for peer review or acceptance for publication.

Upon resubmission, please provide the following:

The name of the colleague or the details of the professional service that edited your manuscript

A copy of your manuscript showing your changes by either highlighting them or using track changes (uploaded as a *supporting information* file)

A clean copy of the edited manuscript (uploaded as the new *manuscript* file)

3. Please include a complete copy of PLOS’ questionnaire on inclusivity in global research in your revised manuscript. Our policy for research in this area aims to improve transparency in the reporting of research performed outside of researchers’ own country or community. The policy applies to researchers who have travelled to a different country to conduct research, research with Indigenous populations or their lands, and research on cultural artefacts. The questionnaire can also be requested at the journal’s discretion for any other submissions, even if these conditions are not met.  Please find more information on the policy and a link to download a blank copy of the questionnaire here: https://journals.plos.org/plosone/s/best-practices-in-research-reporting. Please upload a completed version of your questionnaire as Supporting Information when you resubmit your manuscript.

4. Thank you for stating the following financial disclosure:

“This work was supported by Japan’s Ministry of Health, Labour and Welfare (MHLW) Policy Research for Hepatitis Measures Program Grant Number JPMH22HC1001.”

Please state what role the funders took in the study.  If the funders had no role, please state: "The funders had no role in study design, data collection and analysis, decision to publish, or preparation of the manuscript."

If this statement is not correct you must amend it as needed.

Please include this amended Role of Funder statement in your cover letter; we will change the online submission form on your behalf.

5. We note that Figure 5 in your submission contain [map/satellite] images which may be copyrighted. All PLOS content is published under the Creative Commons Attribution License (CC BY 4.0), which means that the manuscript, images, and Supporting Information files will be freely available online, and any third party is permitted to access, download, copy, distribute, and use these materials in any way, even commercially, with proper attribution. For these reasons, we cannot publish previously copyrighted maps or satellite images created using proprietary data, such as Google software (Google Maps, Street View, and Earth). For more information, see our copyright guidelines: http://journals.plos.org/plosone/s/licenses-and-copyright.

We require you to either (1) present written permission from the copyright holder to publish these figures specifically under the CC BY 4.0 license, or (2) remove the figures from your submission:

a. You may seek permission from the original copyright holder of Figure 5 to publish the content specifically under the CC BY 4.0 license. 

We recommend that you contact the original copyright holder with the Content Permission Form (http://journals.plos.org/plosone/s/file?id=7c09/content-permission-form.pdf) and the following text:

“I request permission for the open-access journal PLOS ONE to publish XXX under the Creative Commons Attribution License (CCAL) CC BY 4.0 (http://creativecommons.org/licenses/by/4.0/). Please be aware that this license allows unrestricted use and distribution, even commercially, by third parties. Please reply and provide explicit written permission to publish XXX under a CC BY license and complete the attached form.”

Please upload the completed Content Permission Form or other proof of granted permissions as an "Other" file with your submission.

In the figure caption of the copyrighted figure, please include the following text: “Reprinted from [ref] under a CC BY license, with permission from [name of publisher], original copyright [original copyright year].”

b. If you are unable to obtain permission from the original copyright holder to publish these figures under the CC BY 4.0 license or if the copyright holder’s requirements are incompatible with the CC BY 4.0 license, please either i) remove the figure or ii) supply a replacement figure that complies with the CC BY 4.0 license. Please check copyright information on all replacement figures and update the figure caption with source information. If applicable, please specify in the figure caption text when a figure is similar but not identical to the original image and is therefore for illustrative purposes only.

The following resources for replacing copyrighted map figures may be helpful:

USGS National Map Viewer (public domain): http://viewer.nationalmap.gov/viewer/

The Gateway to Astronaut Photography of Earth (public domain): http://eol.jsc.nasa.gov/sseop/clickmap/

Maps at the CIA (public domain): https://www.cia.gov/library/publications/the-world-factbook/index.html and https://www.cia.gov/library/publications/cia-maps-publications/index.html

NASA Earth Observatory (public domain): http://earthobservatory.nasa.gov/

Landsat: http://landsat.visibleearth.nasa.gov/

USGS EROS (Earth Resources Observatory and Science (EROS) Center) (public domain): http://eros.usgs.gov/#

Natural Earth (public domain): http://www.naturalearthdata.com/

[Note: HTML markup is below. Please do not edit.]

Reviewers' comments:

Reviewer's Responses to Questions

**Comments to the Author**

1. Is the manuscript technically sound, and do the data support the conclusions?

The manuscript must describe a technically sound piece of scientific research with data that supports the conclusions. Experiments must have been conducted rigorously, with appropriate controls, replication, and sample sizes. The conclusions must be drawn appropriately based on the data presented. 

Reviewer #1: Yes

Reviewer #2: Yes

**********

2. Has the statistical analysis been performed appropriately and rigorously? 

Reviewer #1: Yes

Reviewer #2: Yes

**********

3. Have the authors made all data underlying the findings in their manuscript fully available?

The PLOS Data policy requires authors to make all data underlying the findings described in their manuscript fully available without restriction, with rare exception (please refer to the Data Availability Statement in the manuscript PDF file). The data should be provided as part of the manuscript or its supporting information, or deposited to a public repository. For example, in addition to summary statistics, the data points behind means, medians and variance measures should be available. If there are restrictions on publicly sharing data—e.g. participant privacy or use of data from a third party—those must be specified.

Reviewer #1: Yes

Reviewer #2: Yes

**********

4. Is the manuscript presented in an intelligible fashion and written in standard English?

PLOS ONE does not copyedit accepted manuscripts, so the language in submitted articles must be clear, correct, and unambiguous. Any typographical or grammatical errors should be corrected at revision, so please note any specific errors here.

Reviewer #1: Yes

Reviewer #2: Yes

**********

5. Review Comments to the Author

Please use the space provided to explain your answers to the questions above. You may also include additional comments for the author, including concerns about dual publication, research ethics, or publication ethics. (Please upload your review as an attachment if it exceeds 20,000 characters)

Reviewer #1: Authors made a good attempt at analyzing the prevalence of HCV and the distribution of its genotypes across several populations in Myanmar. The study is properly conducted and the analyses are appropriate. Nonetheless, below are a few points to address/clarify.

1. The study was not registered in a prospective systematic review register such as PROSPERO. Have authors confirmed that there are no similar ongoing studies? If so, it should be clearly stated.

2. Authors should indicate which are ‘studies’ and ‘reports’ from the cited references in Table 1.

3. Authors should consider performing a sensitivity analysis excluding unpublish data in groups where they occur.

4. Line 272: How are the genotype distribution ‘same’ based on the proportion values provided in the sentence? Maybe ‘similar’? Please clarify.

Reviewer #2: The abstract should be rewritten stating the a background, objective of the study. In the Methods, how data wass extracted and the persons involved. Also, the conclusion should reflect the answer to the main objective of the work.

In the Background section; The first paragraph should include what is HCV, before giving the prevalence. Also, Paragraph 2 is not necessary. Paragraph 3 and 4 should be rewritten, including more references and stating clearly the importance of the work before stating the objectives.

In the Material and methods section: All the sentences starting with To, should be rephrased. I have highlighted in the manuscript.

In the results section; I have highlighted in the manuscript, Some sentences should be rephrased, starting appropriately.

In the Discussion section, Same comments as the results section.

Conclusion is Ok.

References: All the references should be rearranged and harmonised.

**********

6. PLOS authors have the option to publish the peer review history of their article (what does this mean?). If published, this will include your full peer review and any attached files.

If you choose “no”, your identity will remain anonymous but your review may still be made public.

**Do you want your identity to be public for this peer review?** For information about this choice, including consent withdrawal, please see our Privacy Policy.

Reviewer #1: No

Reviewer #2: **Yes: **Zeuko'o Menkem Elisabeth

**********

[NOTE: If reviewer comments were submitted as an attachment file, they will be attached to this email and accessible via the submission site. Please log into your account, locate the manuscript record, and check for the action link "View Attachments". If this link does not appear, there are no attachment files.]

While revising your submission, please upload your figure files to the Preflight Analysis and Conversion Engine (PACE) digital diagnostic tool, https://pacev2.apexcovantage.com/. PACE helps ensure that figures meet PLOS requirements. To use PACE, you must first register as a user. Registration is free. Then, login and navigate to the UPLOAD tab, where you will find detailed instructions on how to use the tool. If you encounter any issues or have any questions when using PACE, please email PLOS at figures@plos.org. Please note that Supporting Information files do not need this step.

Attachment Submitted filename: PONE-D-24-10345 Revised Dr Zeukoo.pdf

# Author comment: Author response to Decision Letter 0

**DOI:** 10.1371/journal.pone.0307872.r002

17 Jun 2024

Response to Reviewer Comments

We wish to express our appreciation to the editor, and reviewers for their additional comments on our paper. We revised our paper based on the editor and reviewer’s comment as below.

Response to Reviewer-1

Comment-1: The study was not registered in a prospective systematic review register such as PROSPERO. Have authors confirmed that there are no similar ongoing studies? If so, it should be clearly stated.

Response-1: Thank you so much for your comment. We acknowledge that we did not register the protocol in a systematic review registry. But, before the review, we searched databases such as PubMed, Web of Science, and Scopus for published studies on this topic and found none in PROSPERO, INPLASY, etc. We have clarified this in the manuscript (Page 4, Line 94-98) as below. 

“Though we did not prospectively register our systematic review in the registry database such as International Prospective Register of Systematic Reviews (PROSPERO), International Platform of Registered Systematic Review and Meta-analysis Protocols (INPLASY) etc, there was no ongoing similar studies in those databases. Before the review, we searched databases such as PubMed, Web of Science, and Scopus for published studies on this topic and found none.”

Comment-2: Authors should indicate which are ‘studies’ and ‘reports’ from the cited references in Table 1.

Response-2: We appreciate very much for your valuable comments. We added the symbol “^ in the studies issued from the same report” in the Table 1 and mentioned in the footnote.

Comment-3. Authors should consider performing a sensitivity analysis excluding unpublish data in groups where they occur.

Response-3: Thank you for your comment and important suggestion. We performed a sensitivity analysis by excluding unpublished data. All unpublished data were in low-risk groups only. In the initial analysis, the prevalence of HCV in low-risk groups was 2.18% [1.09% - 3.58%]. After excluding unpublished data, the prevalence increased to 2.41% [ 0.51%; 5.40%] in the low-risk group. This difference is not statistically significant (please see below). We have included the results and discussion of the sensitivity analysis in the manuscript, but we decided not to include the calculation result figure. 

(Result: Page 12, Line 218-220)

“A sensitivity analysis indicated that the prevalence of HCV antibodies increased to 2.41% (95% CI: 0.51%-5.4%) after excluding 3 unpublished reports (n=72,680). However, this difference was not statistically significant (the result not shown).”

(Discussion: Page 17, Line 322-325)

“Despite the sensitivity analysis showing a slight increase in HCV antibody prevalence after removing the unpublished reports in low-risk groups, the change was not statistically significant. However, caution should be exercised when interpreting the pooled prevalence estimates.”

Comment-4: Line 272: How are the genotype distribution ‘same’ based on the proportion values provided in the sentence? Maybe ‘similar’? Please clarify.

Response-4: Thank you so much for your clarification. It was our mistake, and we corrected as “similar”. We have revised in the result section accordingly.

Response to Reviewer-2

Comment-1: The abstract should be rewritten stating the background, objective of the study. In the Methods, how data was extracted, and the persons involved. Also, the conclusion should reflect the answer to the main objective of the work.

Response-1: Thank you very much for your comments, we revised the abstract according to your suggestion. As we had provided the information of person involved in the main manuscript, please let us not include in the abstract. 

Comment-2: In the Background section; The first paragraph should include what is HCV, before giving the prevalence. Also, Paragraph 2 is not necessary. Paragraph 3 and 4 should be rewritten, including more references, and stating clearly the importance of the work before stating the objectives.

Response-2: We appreciate very much for your valuable comments. Based on your suggestions, we deleted paragraph 2, added more references supporting the facts in background and clarified and justified the study aims based on our research title respectively. 

Comment-3: In the Material and methods section: All the sentences starting with To, should be rephrased. I have highlighted in the manuscript.

Response-3: Thank you very much for your intensive review. Based on your suggestion, we revised all the statements started with “To” appropriately. We appreciate very much for your valuable comments. 

Comment-4: In the results section; I have highlighted in the manuscript, Some sentences should be rephrased, starting appropriately.

Response-4: Thank you very much for your valuable suggestion, we have rephrased them accordingly. 

Comment-5: In the Discussion section, Same comments as the results section.

Response-5: Thank you very much for your insightful suggestion, we rephrased and revised the statement as you commented details in the manuscript. 

Comment-6: Conclusion is Ok.

Response-6: Thank you very much for your comment, we appreciate. 

Comment-7: References: All the references should be rearranged and harmonised.

Response-7: Thank you very much for your comment and all the references were also harmonized according to the journal’s requirement. 

Response to the editorial office

Comment-1. Please ensure that your manuscript meets PLOS ONE's style requirements, including those for file naming. The PLOS ONE style templates can be found at

https://journals.plos.org/plosone/s/file?id=wjVg/PLOSOne_formatting_sample_main_body.pdf and

https://journals.plos.org/plosone/s/file?id=ba62/PLOSOne_formatting_sample_title_authors_affiliations.pdf

Response-1: Thank you very much for your comments, we formatted the manuscript based on the instruction. 

Comment-2. We suggest you thoroughly copyedit your manuscript for language usage, spelling, and grammar. If you do not know anyone who can help you do this, you may wish to consider employing a professional scientific editing service.

The American Journal Experts (AJE) (https://www.aje.com/) is one such service that has extensive experience helping authors meet PLOS guidelines and can provide language editing, translation, manuscript formatting, and figure formatting to ensure your manuscript meets our submission guidelines. Please note that having the manuscript copyedited by AJE or any other editing services does not guarantee selection for peer review or acceptance for publication.

Upon resubmission, please provide the following:

The name of the colleague or the details of the professional service that edited your manuscript

A copy of your manuscript showing your changes by either highlighting them or using track changes (uploaded as a *supporting information* file)

A clean copy of the edited manuscript (uploaded as the new *manuscript* file)

Response-2: Thank you very much for your comments, ZP and KK, the first and coauthor of this manuscript did the intensive edition, and we provided the revised version in both clean and with tract change. 

Comment-3. Please include a complete copy of PLOS’ questionnaire on inclusivity in global research in your revised manuscript. Our policy for research in this area aims to improve transparency in the reporting of research performed outside of researchers’ own country or community. The policy applies to researchers who have travelled to a different country to conduct research, research with Indigenous populations or their lands, and research on cultural artefacts. The questionnaire can also be requested at the journal’s discretion for any other submissions, even if these conditions are not met. Please find more information on the policy and a link to download a blank copy of the questionnaire here: https://journals.plos.org/plosone/s/best-practices-in-research-reporting. Please upload a completed version of your questionnaire as Supporting Information when you resubmit your manuscript.

Response-3: Thank you very much for your comments, we provided the checklist for the Inclusivity in global research questionnaire as Supporting Information and mentioned in the method section with sub-heading (Page 6, Line 150-152).

“Inclusivity in global research: Additional information regarding the ethical, cultural, and scientific considerations specific to inclusivity in global research is included in the Supporting Information (S4 Checklist).”

Comment-4. Thank you for stating the following financial disclosure:

“This work was supported by Japan’s Ministry of Health, Labour and Welfare (MHLW) Policy Research for Hepatitis Measures Program Grant Number JPMH22HC1001.”

Please state what role the funders took in the study. If the funders had no role, please state: "The funders had no role in study design, data collection and analysis, decision to publish, or preparation of the manuscript."

If this statement is not correct you must amend it as needed.

Please include this amended Role of Funder statement in your cover letter; we will change the online submission form on your behalf.

Response-4: Thank you very much for your comments, we added this funder role statement in the manuscript and in the cover letter. Using this opportunity, we would like to change the funding source as “This work was supported by Japan Society for the Promotion of Science (JSPS) Core-to-Core Program (Grant number: JPJSCCB20240009). The funders had no role in study design, data collection and analysis, decision to publish, or preparation of the manuscript”. 

Comment-5. We note that Figure 5 in your submission contain [map/satellite] images which may be copyrighted. All PLOS content is published under the Creative Commons Attribution License (CC BY 4.0), which means that the manuscript, images, and Supporting Information files will be freely available online, and any third party is permitted to access, download, copy, distribute, and use these materials in any way, even commercially, with proper attribution. For these reasons, we cannot publish previously copyrighted maps or satellite images created using proprietary data, such as Google software (Google Maps, Street View, and Earth). For more information, see our copyright guidelines: http://journals.plos.org/plosone/s/licenses-and-copyright.

We require you to either (1) present written permission from the copyright holder to publish these figures specifically under the CC BY 4.0 license, or (2) remove the figures from your submission:

a. You may seek permission from the original copyright holder of Figure 5 to publish the content specifically under the CC BY 4.0 license. 

We recommend that you contact the original copyright holder with the Content Permission Form (http://journals.plos.org/plosone/s/file?id=7c09/content-permission-form.pdf) and the following text:

“I request permission for the open-access journal PLOS ONE to publish XXX under the Creative Commons Attribution License (CCAL) CC BY 4.0 (http://creativecommons.org/licenses/by/4.0/). Please be aware that this license allows unrestricted use and distribution, even commercially, by third parties. Please reply and provide explicit written permission to publish XXX under a CC BY license and complete the attached form.”

Please upload the completed Content Permission Form or other proof of granted permissions as an "Other" file with your submission.

In the figure caption of the copyrighted figure, please include the following text: “Reprinted from [ref] under a CC BY license, with permission from [name of publisher], original copyright [original copyright year].”

b. If you are unable to obtain permission from the original copyright holder to publish these figures under the CC BY 4.0 license or if the copyright holder’s requirements are incompatible with the CC BY 4.0 license, please either i) remove the figure or ii) supply a replacement figure that complies with the CC BY 4.0 license. Please check copyright information on all replacement figures and update the figure caption with source information. If applicable, please specify in the figure caption text when a figure is similar but not identical to the original image and is therefore for illustrative purposes only.

The following resources for replacing copyrighted map figures may be helpful:

USGS National Map Viewer (public domain): http://viewer.nationalmap.gov/viewer/

The Gateway to Astronaut Photography of Earth (public domain): http://eol.jsc.nasa.gov/sseop/clickmap/

Maps at the CIA (public domain): https://www.cia.gov/library/publications/the-world-factbook/index.html and https://www.cia.gov/library/publications/cia-maps-publications/index.html

NASA Earth Observatory (public domain): http://earthobservatory.nasa.gov/

Landsat: http://landsat.visibleearth.nasa.gov/

USGS EROS (Earth Resources Observatory and Science (EROS) Center) (public domain): http://eros.usgs.gov/#

Natural Earth (public domain): http://www.naturalearthdata.com/

 Response-5: Thank you very much for your comments. In fact, we created the map by ourselves using the free online based creation at http://n.freemap.jp. Therefore, no copyright issue to use the map from that abovementioned free webpage. But, for the printing of the colored map what we created, the copyright belongs to us.

Attachment Submitted filename: 240617 Response to ReviewersV2.pdf

# Peer review: Decision Letter 1

**DOI:** 10.1371/journal.pone.0307872.r003

**Authors:** Yury E Khudyakov

10 Jul 2024

PONE-D-24-10345R1Intermediate hepatitis C virus (HCV) endemicity and its genotype distribution in Myanmar: A systematic review and meta-analysisPLOS ONE

Dear Dr. Tanaka,

Thank you for submitting your manuscript to PLOS ONE. After careful consideration, we feel that it has merit but does not fully meet PLOS ONE’s publication criteria as it currently stands. Therefore, we invite you to submit a revised version of the manuscript that addresses the points raised during the review process.

==============================

Your revised manuscript was reviewed by the original reviewers. One reviewer identified some problems still remaining in your paper. Please consider the attached comments and provide point-by-point responses.  

==============================

Please submit your revised manuscript by Aug 24 2024 11:59PM. If you will need more time than this to complete your revisions, please reply to this message or contact the journal office at plosone@plos.org. When you're ready to submit your revision, log on to https://www.editorialmanager.com/pone/ and select the 'Submissions Needing Revision' folder to locate your manuscript file.

Please include the following items when submitting your revised manuscript:

- A rebuttal letter that responds to each point raised by the academic editor and reviewer(s). You should upload this letter as a separate file labeled 'Response to Reviewers'.
- A marked-up copy of your manuscript that highlights changes made to the original version. You should upload this as a separate file labeled 'Revised Manuscript with Track Changes'.
- An unmarked version of your revised paper without tracked changes. You should upload this as a separate file labeled 'Manuscript'.

If you would like to make changes to your financial disclosure, please include your updated statement in your cover letter. Guidelines for resubmitting your figure files are available below the reviewer comments at the end of this letter.

If applicable, we recommend that you deposit your laboratory protocols in protocols.io to enhance the reproducibility of your results. Protocols.io assigns your protocol its own identifier (DOI) so that it can be cited independently in the future. For instructions see: https://journals.plos.org/plosone/s/submission-guidelines#loc-laboratory-protocols. Additionally, PLOS ONE offers an option for publishing peer-reviewed Lab Protocol articles, which describe protocols hosted on protocols.io. Read more information on sharing protocols at https://plos.org/protocols?utm_medium=editorial-email&utm_source=authorletters&utm_campaign=protocols.

We look forward to receiving your revised manuscript.

Kind regards,

Yury E Khudyakov, PhD

Academic Editor

PLOS ONE

Journal Requirements:

Please review your reference list to ensure that it is complete and correct. If you have cited papers that have been retracted, please include the rationale for doing so in the manuscript text, or remove these references and replace them with relevant current references. Any changes to the reference list should be mentioned in the rebuttal letter that accompanies your revised manuscript. If you need to cite a retracted article, indicate the article’s retracted status in the References list and also include a citation and full reference for the retraction notice.

[Note: HTML markup is below. Please do not edit.]

Reviewers' comments:

Reviewer's Responses to Questions

**Comments to the Author**

1. If the authors have adequately addressed your comments raised in a previous round of review and you feel that this manuscript is now acceptable for publication, you may indicate that here to bypass the “Comments to the Author” section, enter your conflict of interest statement in the “Confidential to Editor” section, and submit your "Accept" recommendation.

Reviewer #2: All comments have been addressed

**********

2. Is the manuscript technically sound, and do the data support the conclusions?

The manuscript must describe a technically sound piece of scientific research with data that supports the conclusions. Experiments must have been conducted rigorously, with appropriate controls, replication, and sample sizes. The conclusions must be drawn appropriately based on the data presented. 

Reviewer #2: Yes

**********

3. Has the statistical analysis been performed appropriately and rigorously? 

Reviewer #2: Yes

**********

4. Have the authors made all data underlying the findings in their manuscript fully available?

The PLOS Data policy requires authors to make all data underlying the findings described in their manuscript fully available without restriction, with rare exception (please refer to the Data Availability Statement in the manuscript PDF file). The data should be provided as part of the manuscript or its supporting information, or deposited to a public repository. For example, in addition to summary statistics, the data points behind means, medians and variance measures should be available. If there are restrictions on publicly sharing data—e.g. participant privacy or use of data from a third party—those must be specified.

Reviewer #2: Yes

**********

5. Is the manuscript presented in an intelligible fashion and written in standard English?

PLOS ONE does not copyedit accepted manuscripts, so the language in submitted articles must be clear, correct, and unambiguous. Any typographical or grammatical errors should be corrected at revision, so please note any specific errors here.

Reviewer #2: Yes

**********

6. Review Comments to the Author

Please use the space provided to explain your answers to the questions above. You may also include additional comments for the author, including concerns about dual publication, research ethics, or publication ethics. (Please upload your review as an attachment if it exceeds 20,000 characters)

Reviewer #2: The abstract should be segmented with the different headings: Background, Material and Methods, Results and conclusion. The keywords should be arranged alphabetically. The references should be checked, completed and harmonised following the journal guidelines.

**********

7. PLOS authors have the option to publish the peer review history of their article (what does this mean?). If published, this will include your full peer review and any attached files.

If you choose “no”, your identity will remain anonymous but your review may still be made public.

**Do you want your identity to be public for this peer review?** For information about this choice, including consent withdrawal, please see our Privacy Policy.

Reviewer #2: **Yes: **Zeuko'o Menkem Elisabeth

**********

[NOTE: If reviewer comments were submitted as an attachment file, they will be attached to this email and accessible via the submission site. Please log into your account, locate the manuscript record, and check for the action link "View Attachments". If this link does not appear, there are no attachment files.]

While revising your submission, please upload your figure files to the Preflight Analysis and Conversion Engine (PACE) digital diagnostic tool, https://pacev2.apexcovantage.com/. PACE helps ensure that figures meet PLOS requirements. To use PACE, you must first register as a user. Registration is free. Then, login and navigate to the UPLOAD tab, where you will find detailed instructions on how to use the tool. If you encounter any issues or have any questions when using PACE, please email PLOS at figures@plos.org. Please note that Supporting Information files do not need this step.

Attachment Submitted filename: PONE-D-24-10345_R1 Revised DrZeukoo.pdf

# Author comment: Author response to Decision Letter 1

**DOI:** 10.1371/journal.pone.0307872.r004

12 Jul 2024

Response to Reviewers’ Comments

We wish to express our appreciation to the editor, and reviewer for their additional comments on our paper. We revised our paper based on the editor and reviewer’s comment as below.

Response to Reviewer-2

Comment-1: The abstract should be segmented with the different headings: Background, Material and Methods, Results and conclusion.

Response-1: Thank you very much for your comments, we added subheading in the abstract. 

Comment-2: The keywords should be arranged alphabetically. 

Response-2: We appreciate very much for your comments. Based on your suggestions, we rearranged them.

Comment-3: The references should be checked, completed and harmonised following the journal guidelines. 

Response-3: Thank you very much for your intensive review. Based on your suggestion, we checked all the references and revised reference number 1 to 5 and 47 based on the journal guidelines.

Response to the Editorial office

Comment-1. Please review your reference list to ensure that it is complete and correct. If you have cited papers that have been retracted, please include the rationale for doing so in the manuscript text, or remove these references and replace them with relevant current references. Any changes to the reference list should be mentioned in the rebuttal letter that accompanies your revised manuscript. If you need to cite a retracted article, indicate the article’s retracted status in the References list and also include a citation and full reference for the retraction notice. 

Response-1: Thank you for your detailed review. We have carefully examined our reference list and confirm that our manuscript does not cite any retracted papers. Based on your suggestions, we have checked all the references and revised the reference no. 1-5, 9, 19, 22, 36, and 47 according to the journal guidelines. Specifically, reference no. 9, 19, 22, and 36 were in an online article format, and we have provided the corresponding URL link in all the revised references.

Attachment Submitted filename: Response to Reviewers Final.pdf

# Editor report: Decision Letter 2

**DOI:** 10.1371/journal.pone.0307872.r005

**Authors:** Yury E Khudyakov

15 Jul 2024

Intermediate hepatitis C virus (HCV) endemicity and its genotype distribution in Myanmar: A systematic review and meta-analysis

PONE-D-24-10345R2

Dear Dr. Tanaka,

We’re pleased to inform you that your manuscript has been judged scientifically suitable for publication and will be formally accepted for publication once it meets all outstanding technical requirements.

Within one week, you’ll receive an e-mail detailing the required amendments. When these have been addressed, you’ll receive a formal acceptance letter and your manuscript will be scheduled for publication.

An invoice will be generated when your article is formally accepted. Please note, if your institution has a publishing partnership with PLOS and your article meets the relevant criteria, all or part of your publication costs will be covered. Please make sure your user information is up-to-date by logging into Editorial Manager at Editorial Manager® and clicking the ‘Update My Information' link at the top of the page. If you have any questions relating to publication charges, please contact our Author Billing department directly at authorbilling@plos.org.

If your institution or institutions have a press office, please notify them about your upcoming paper to help maximize its impact. If they’ll be preparing press materials, please inform our press team as soon as possible -- no later than 48 hours after receiving the formal acceptance. Your manuscript will remain under strict press embargo until 2 pm Eastern Time on the date of publication. For more information, please contact onepress@plos.org.

Kind regards,

Yury E Khudyakov, PhD

Academic Editor

PLOS ONE

Additional Editor Comments (optional):

Reviewers' comments:

# Editor report: Acceptance letter

**DOI:** 10.1371/journal.pone.0307872.r006

**Authors:** Yury E Khudyakov

2 Aug 2024

PONE-D-24-10345R2 

PLOS ONE

Dear Dr. Tanaka, 

I'm pleased to inform you that your manuscript has been deemed suitable for publication in PLOS ONE. Congratulations! Your manuscript is now being handed over to our production team.

At this stage, our production department will prepare your paper for publication. This includes ensuring the following:

* All references, tables, and figures are properly cited

* All relevant supporting information is included in the manuscript submission,

* There are no issues that prevent the paper from being properly typeset

If revisions are needed, the production department will contact you directly to resolve them. If no revisions are needed, you will receive an email when the publication date has been set. At this time, we do not offer pre-publication proofs to authors during production of the accepted work. Please keep in mind that we are working through a large volume of accepted articles, so please give us a few weeks to review your paper and let you know the next and final steps. 

Lastly, if your institution or institutions have a press office, please let them know about your upcoming paper now to help maximize its impact. If they'll be preparing press materials, please inform our press team within the next 48 hours. Your manuscript will remain under strict press embargo until 2 pm Eastern Time on the date of publication. For more information, please contact onepress@plos.org.

If we can help with anything else, please email us at customercare@plos.org.

Thank you for submitting your work to PLOS ONE and supporting open access. 

Kind regards, 

PLOS ONE Editorial Office Staff

on behalf of

Dr. Yury E Khudyakov 

Academic Editor

PLOS ONE
